# Laburnicotides A–F: Acyclic *N*-Acetyl Oligopeptides from the Nematode-Cyst-Associated Fungus *Laburnicola nematophila*

**DOI:** 10.1021/acsomega.4c02719

**Published:** 2024-05-06

**Authors:** Caren Holzenkamp, Jan-Peer Wennrich, Jackson M. Muema, Samad Ashrafi, Wolfgang Maier, Marc Stadler, Sherif S. Ebada

**Affiliations:** † Department of Microbial Drugs, Helmholtz Centre for Infection Research (HZI) and German Centre for Infection Research, Inhoffenstrasse 7, Braunschweig 38124, Germany; ‡ Institute of Microbiology, Technische Universität Braunschweig, Spielmannstraße 7, Braunschweig 38106, Germany; § Compound Profiling and Screening (COPS), Helmholtz Centre for Infection Research (HZI), Inhoffenstrasse 7, Braunschweig 38124, Germany; ⊥ Institute for Epidemiology and Pathogen Diagnostics, Julius Kühn Institute (JKI)−Federal Research Center for Cultivated Plants, Messeweg 11-12, Braunschweig 38104, Germany; ∇ Institute for Crop and Soil Science, Julius Kühn Institute (JKI)−Federal Research Centre for Cultivated Plants, Bundesallee 58, Braunschweig 38116, Germany; ¶ Department of Pharmacognosy, Faculty of Pharmacy, Ain Shams University, Cairo 11566, Egypt

## Abstract

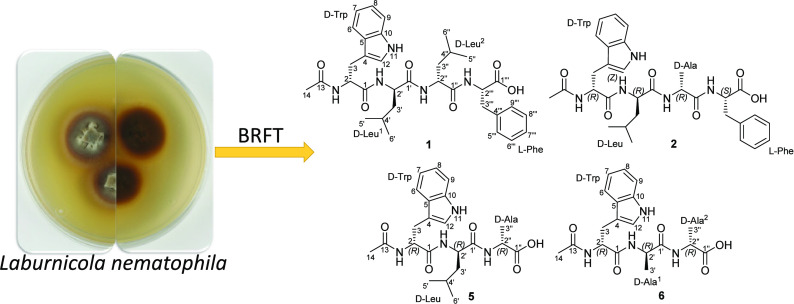

Nematode-associated fungi revealed the potential to produce a broad spectrum of chemical scaffolds. In this study, a mycelial extract of *Laburnicola nematophila*, a fungal strain derived from the cereal cyst nematode *Heterodera filipjevi*, was chemically explored and afforded six unprecedentedly reported acylic *N*-acetyl oligopeptides, laburnicotides A–F (**1**–**6**). Structure elucidation of the isolated compounds was established based on comprehensive 1D and 2D NMR spectroscopic analyses together with the acquired HR-ESI-MS spectrometric data. The absolute configuration of amino acid residues in **1**–**6** was established by performing advanced Marfey’s derivatization method. All isolated compounds were assessed for their cytotoxic, antimicrobial, antiviral, and nematicidal activities with no potential activity observed.

## Introduction

Fungal secondary metabolites exhibit remarkable structural diversity and find extensive applications in medicine, food industry, and agriculture.^1,2^ Chemically, these metabolites belong to different classes including fatty acids, terpenoids, alkaloids, polyketides, or peptides.^3,4^ Nitrogenous metabolites such as peptides and alkaloids are generally able to exert diverse physiological functions, making them valuable candidates for success stories in developing drugs, functional foods, or agrochemicals.^5,6^ In the medical field, more than 80 peptides have gained approval as pharmaceuticals targeting diseases like diabetes, cancer, osteoporosis, multiple sclerosis, or HIV infections.^7^ Due to their diverse activities together with high environmental compatibility and nontoxicity, they have recently sparked increasing interest in the agricultural sector.^5,8^ Eighteen peptides have been commercialized for use as plant protection agents, with more under development.^5^ They can exhibit various modes of action including immune induction, antimicrobial effects,^9^ or as plant growth regulators,^5^ nematicides,^9,10^ insecticides,^11^ and herbicides.^12^ During the course of our research targeting bioactive secondary metabolites of potential anti-infective and/or nematicidal activities, we explored the recently described cyst-associated fungus *Laburnicola nematophila* (*L. nematophila*)^13^ derived from the plant parasitic nematode *Heterodera filipjevi* (*H. filipjevi*) to ultimately develop a biocontrol agent.

Within the Ascomycetes, *L. nematophila* belongs to the class Dothideomycetes and the order Pleosporales.^13,14^ The genus *Laburnicola* was first reported by Wanasinghe et al. on different plant families.^15^ A few representatives of *Laburnicola* have been reported to exhibit also endophytic interactions with their host plants.^13^ The nematode parasitic fungus *L. nematophila* and other recently reported fungi *Polyphilus sieberi* and *Polydomus karssenii* have been studied for their production of secondary metabolites, positioning these nematode-associated fungi as a compelling subject for investigation.^16−19^ Herein, we report the chemical and biological characterization of six undescribed acyclic *N*-acetyl oligopeptides (**1**–**6**) from two different strains of *L. nematophila* (20AD and K01).
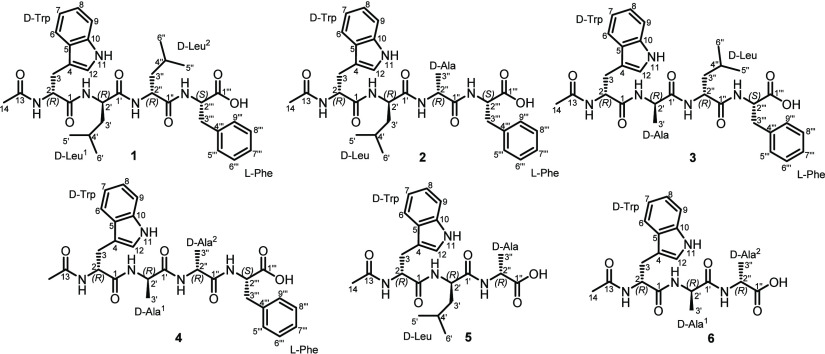


## Results and Discussion

### Structure Elucidation of Compounds **1**–**6**

**Figure 1:**
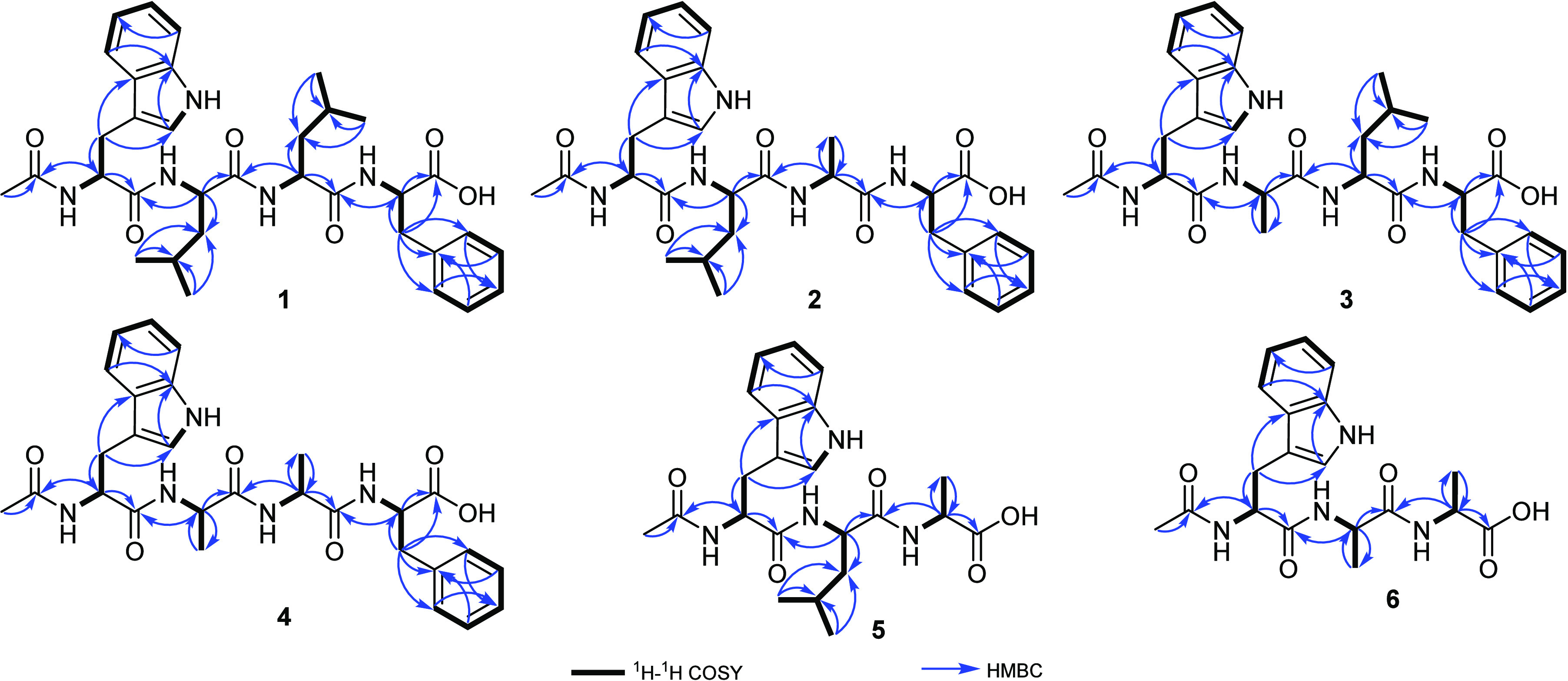
Key ^1^H–^1^H COSY and HMBC correlations of **1**–**6**.

**Figure 2:**
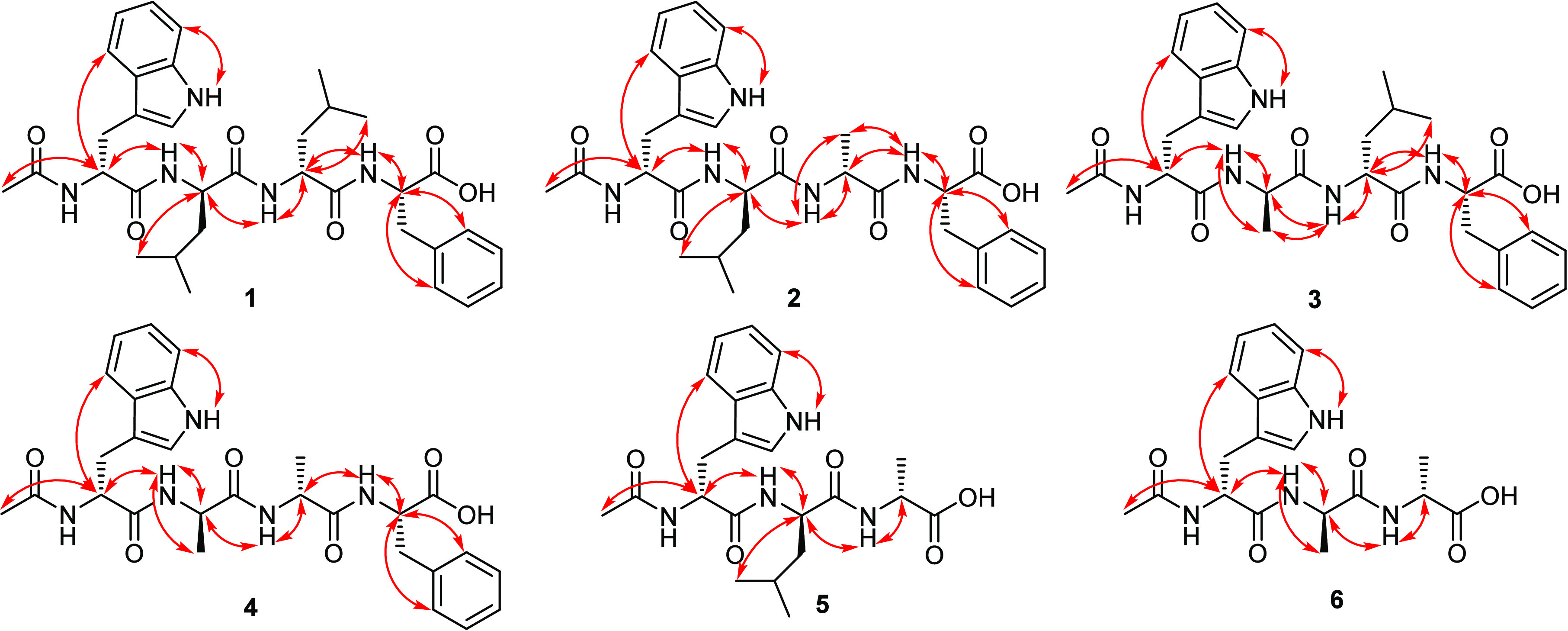
Key ROESY correlations of compounds **1**–**6**.

Compound **1** was isolated as a pale brown, amorphous solid. Its molecular formula was established as C_34_H_45_N_5_O_6_ based on HR-ESI-MS that revealed a protonated molecular ion peak at *m*/*z* 620.3439 [M + H]^+^ (calculated 620.3443) and a sodium adduct at *m*/*z* 642.3258 [M + Na]^+^ (calculated 642.3262) indicating 15 degrees of unsaturation. The ^1^H NMR and ^1^H–^1^H COSY spectra of **1** in DMSO-*d*_6_ (Table 1, Figure 1, and Figure S5) revealed characteristic features of a peptide including the presence of four exchangeable amide NH signals (δ_H_ 7.44–8.21) together with four amino acid α-proton signals (δ_H_ 4.00–4.55) and four β-methylene groups (δ_H_ 2.85/3.10, 2.86/3.04, 1.25, 1.42) suggesting a peptide skeleton with four amino acid residues for **1**. In addition, the ^1^H–^1^H COSY spectrum of **1** (Figure 1 and Figure S5) revealed key correlations of a 1,2-disubstituted aromatic ring through a spin system starting at δ_H_ 7.58 (d, *J* = 7.9 Hz) extending to δ_H_ 6.95 (td, *J* = 7.8, 1.8 Hz), δ_H_ 7.03 (ddd, *J* = 8.0, 7.8, 1.2 Hz), and δ_H_ 7.31 (d, *J* = 8.0 Hz) in addition to a key correlation between a deshielded NH signal at δ_H_ 11.05 and an olefinic proton at δ_H_ 7.04 (d, *J* = 2.0 Hz) suggesting the presence of an indole group indicating a tryptophan (Trp) residue. The ^1^H–^1^H COSY spectrum of **1** (Figure 1 and Figure S5) unraveled the presence of a monosubstituted aromatic ring by featuring a spin system among three proton signals with a total integration index of five at δ_H_ 7.10–7.15 suggesting the presence of a phenylalanine (Phe) residue in **1**. Apart from Trp and Phe residues, the ^1^H–^1^H COSY spectrum of **1** (Figure 1 and Figure S5) disclosed the presence of two different spin systems each extending from an α-proton to a β-methylene then to a γ-proton and ending by two doublet methyl groups (δ_1_/δ_2_) suggesting the presence of two leucine (Leu^1^/Leu^2^) residues. The ^13^C NMR spectral data of **1** (Table 1) revealed the presence of five different carbonyl carbons (δ_C_ 172.6, 171.6, 171.4, 170.3, and 169.0). The latter carbonyl carbon revealed key HMBC correlations (Figure 1) to a singlet methyl group at δ_H_ 1.74 and a proton signal at δ_H_ 4.55 (td, *J* = 8.4, 5.5 Hz, H-2) ascribed to the αH of Trp residue by its HMBC correlation to C-4 (δ_C_ 110.0). These results indicated that compound **1** is an acyclic oligopeptide comprising four amino acids with one *N*-acetyl terminus directly connected to the Trp residue. The HMBC spectrum of **1** (Figure 1) also revealed a key correlation from an α-proton at δ_H_ 4.22 (q, *J* = 6.4 Hz, H-2‴) to an unprotonated sp^2^ carbon at δ_C_ 139.5 (C-4‴) and a carboxyl carbon at δ_C_ 172.6 (C-1‴) indicating its presence as a COOH terminus on the Phe residue. Further confirmation to the amino acid sequence of **1** was provided by the HMBC spectrum (Figure 1) revealing key correlations from H-2 to C-13 (δ_C_ 169.0) and C-1 (δ_C_ 171.4), while the latter and C-1’ (δ_C_ 171.6) revealed key correlation to H-2’ at δ_H_ 4.28 (q, *J* = 7.9 Hz) in the Leu^1^ residue. In addition, H-2” at δ_H_ 4.19 (td, *J* = 8.6, 5.5 Hz) revealed key HMBC correlations to C-1’ and C-1” (δ_C_ 170.3) that in turn and alongside with C-1‴ (δ_C_ 172.6) were correlated to H-2‴ at δ_H_ 4.00 (q, *J* = 6.4 Hz). The amino acid sequence in **1** was further confirmed by the ROESY spectrum (Figure 2 and Figure S8) that revealed key ROE correlations from H-2 to H_3_-14 (δ_H_ 1.74, s), NH-Trp (δ_H_ 8.18, d, *J* = 8.2 Hz), and NH-Leu^1^ (δ_H_ 8.21, br s), whereas H-2” revealed key ROE correlations to NH-Leu^2^ (δ_H_ 7.80, br s) and NH-Phe (δ_H_ 7.44, d, *J* = 7.1 Hz). According to the obtained results, compound **1** was elucidated as an acyclic *N*-acetyl tetrapeptide of Trp-Leu^1^-Leu^2^-Phe. To determine the absolute configuration of amino acid residues in **1**, advanced Marfey’s method was performed.^20^ The comparison of retention times (Figures S53 and S54) between the authentic amino acid standards and the hydrolysate of **1** assigned the absolute configuration of both leucine residues as the d-configuration, while phenylalanine turned out to be of the l-configuration. To determine the absolute configuration of Trp, it was chemically converted to aspartic acid^21^ and then subjected to Marfey’s method that confirmed it to be of d-configuration.^20^ Based on the aforementioned results, compound **1** was unambiguously determined to be a previously undescribed acyclic *N*-acetyl tetrapeptide Ac-d-Trp-d-Leu^1^-d-Leu^2^-l-Phe, and it was given the trivial name laburnicotide A.

**Table 1:** ^1^H and ^13^C NMR Data of **1** and **2**

	**1**		**2**		
position	δ_C_,^a^ type	δ_H_^a^ (multi, *J* [Hz])	δ_C_,^b^ type	δ_H_^b^ (multi, *J* [Hz])	δ_H_^a^ (multi, *J* [Hz])
Trp					
1	171.4, CO		174.3, CO		
2	53.3, CH	4.55 td (8.4, 5.5)	55.6, CH	4.69 td (7.9, 6.0)	4.55 td (8.2, 5.9)
3	27.6, CH_2_	α 2.85 dd (14.7, 8.4)	28.6, CH_2_	α 3.10 dd (14.8, 7.9)	α 2.85 dd (14.8, 8.5)
		β 3.10 dd (14.7, 5.5)		β 3.28 dd (14.8, 6.0)	β 3.12 dd (14.7, 5.8)
4	110.0, C		110.9, C		
5	127.4, C		128.9, C		
6	118.4, CH	7.58 d (7.9)	119.4, CH	7.61 d (7.9, 1.1)	7.58 d (7.9)
7	118.0, CH	6.95 td (7.8, 1.8)	119.8, CH	7.01 td (8.0, 1.0)	6.95 t (7.4)
8	120.6, CH	7.03 ddd (8.0, 7.8, 1.2)	122.4, CH	7.08 td (8.1, 1.0)	7.03 t (7.5)
9	111.4, CH	7.31 d (8.0)	112.3, CH	7.32 d (8.1)	7.31 d (8.1)
10	136.1, C		138.0, C		
11 (NH)	-	11.05 s	-	-	11.04 s
12	123.4, CH	7.04 d (2.0)	124.5, CH	7.09 s	7.04 d (2.6)
NH	-	8.18 d (8.2)	-	-	8.08 d (8.0)
13	169.0, CO		173.3, CO		
14	22.5, CH_3_	1.74 s	22.5, CH_3_	1.91 s	1.77 s
Leu^1^/Leu					
1′	171.6, CO		174.2, CO		
2′	51.18, CH	4.28 q (7.9)	53.2, CH	4.33 dd (9.5, 5.5)	4.27 q (7.8)
3′	40.4, CH_2_	1.42 t (7.6)	41.6, CH_2_	1.47 dt (9.1, 5.3)	1.42 t (7.3)
4′	24.1, CH	1.53 td (13.4, 6.7)	25.7, CH	1.52 m	1.54 m
5′	21.6, CH_3_	0.80 d (6.5)	21.8, CH_3_	0.84 d (6.4)	0.81 d (6.7)
6′	23.1, CH_3_	0.84 d (6.5)	23.5, CH_3_	0.87 d (6.4)	0.84 d (6.7)
NH	-	8.21 br s	-	-	8.09 d (7.0)
Leu^2^/Ala					
1″	170.3, CO		174.0, CO		
2″	51.22, CH	4.19 td (8.6, 5.5)	50.2, CH	4.29 q (7.2)	4.17 p (7.0)
3″	41.3, CH_2_	1.25 m	18.2, CH_3_	1.17 d (7.2)	1.01 d (7.0)
4″	23.9, CH	1.38 dd (14.6, 7.6)			
5″	21.7, CH_3_	0.73 d (6.5)			
6″	23.0, CH_3_	0.76 d (6.5)			
NH	-	7.80 br s	-	-	7.77 d (7.4)
Phe					
1‴	172.6, CO		175.8, CO		
2‴	55.6, CH	4.00 q (6.4)	55.9, CH	4.58 dd (8.0, 4.8)	3.99 q (6.3, 1H)
3‴	37.7, CH_2_	α 2.86 overlapped	38.7, CH_2_	α 3.01 dd (13.8, 8.0)	α 2.86 dd (13.3, 8.0)
		β 3.04 dd (13.3, 4.9)		β 3.22 dd (13.8, 4.8)	β 3.05 dd (13.3, 4.9)
4‴	139.5, C		138.7, C		
5‴, 9‴	129.5, CH	7.11 m	130.6, CH	7.21 m	7.11 d (7.6)
6‴, 8‴	127.5, CH	7.15 m	129.3, CH	7.24 m	7.16 t (7.5)
7‴	125.4, CH	7.10 m	127.6, CH	7.17 tt (7.0, 1.7)	7.09 t (7.3)
NH	-	7.44 d (7.1)	-	-	7.43 d (7.0)

a Measured in DMSO-*d*_6_ at 175 MHz for ^13^C and 700 MHz for ^1^H.

b Measured in methanol-*d*_4_ at 175 MHz for ^13^C and 700 MHz for ^1^H.

Compound **2** was purified as a pale-brown, amorphous solid. The HR-ESI-MS spectrum revealed a protonated molecular ion peak at *m*/*z* 578.2971 [M + H]^+^ (calculated 578.2973) and a sodium adduct at *m*/*z* 600.2790 [M + Na]^+^ (calculated 600.2793) determining the molecular formula of **2** as C_31_H_39_N_5_O_6_ indicating 15 degrees of unsaturation equal to those of **1**, despite having a lesser molecular formula by the C_3_H_6_ moiety reflecting the difference of 42 Da in their molecular masses. The ^1^H and ^13^C NMR spectral data of **2** (Table 1) revealed features similar to those in **1** suggesting its common nature of being an acylic *N*-acetyl oligopeptide. A careful interpretation of ^1^H NMR, ^1^H–^1^H COSY, and ROESY spectral data of **2** (Table 1 and Figures 1 and 2) in methanol-*d*_4_ and DMSO-*d*_6_ suggested the presence of four amino acid residues, namely, Trp, Phe, and Leu as in **1** while the fourth was identified as an alanine (Ala) residue by revealing a characteristic spin system extending from an α-proton signal at δ_H_ 4.29 (q, *J* = 7.2 Hz, H-2″) to a doublet methyl group at δ_H_ 1.17 (d, *J* = 7.2 Hz, H_3_-3″). The substitution of one Leu in **1** by Ala in **2** accounted for the difference of C_3_H_6_ between their molecular formulas. To determine the amino acid sequence in **2**, its HMBC spectrum was acquired (Figure 1), and it revealed key correlations from H-2 at δ_H_ 4.69 (td, *J* = 7.9, 6.0 Hz) assigned as αH-Trp to four carbon atoms ascribed to C-1 (δ_C_ 174.3), C-3 (δ_C_ 28.6), C-4 (δ_C_ 110.9), and C-13 (δ_C_ 173.3), whereas the latter revealed key correlation to a singlet methyl at δ_H_ 1.91 (s, H_3_-14; δ_C_ 22.5) indicating its presence as a *N*-acetyl terminus connected to the Trp residue. In addition, the HMBC spectrum of **2** (Figure 1) disclosed key correlations from H-2′ (δ_H_ 4.33, dd, *J* = 9.5, 5.5 Hz, αH-Leu) to C-1 and C-1′ (δ_C_ 174.2). The HMBC spectrum revealed key correlations from C-1′ and C-1″ (δ_C_ 174.0) to H-2″. In addition, H-2‴ (δ_H_ 4.58, dd, *J* = 8.0, 4.8 Hz, αH-Phe) revealed key HMBC correlations to C-1″ and a terminal carboxylic acid carbon at δ_C_ 175.8 (C-1‴). Furthermore, the amino acid sequence in **2** was confirmed by its ROESY spectrum in DMSO-*d*_6_ (Figure 2 and Figure S18) that revealed key ROE correlations comparable to those revealed in **1**, especially those correlations from H_3_-14 to NH-Trp at δ_H_ 8.08 (d, *J* = 8.0 Hz) that in turn exhibited key ROE correlations together with NH-Leu at δ_H_ 8.09 (d, *J* = 7.0 Hz) to H-2 and H-2′. Then, H-2′ and H-2″ revealed key ROE correlation to NH-Ala (δ_H_ 7.77, d, *J* = 7.4 Hz) and NH-Phe (δ_H_ 7.43, d, *J* = 7.0 Hz), respectively. Based on these results, compound **2** was established as Ac-Trp-Leu-Ala-Phe. The absolute configuration of amino acid residues other than Trp in **2** was unambiguously recognized using Marfey’s method.^20^ By comparing the retention times obtained with authentic amino acid standards and those from the hydrolysate of **2** (Figures S53–S55), we determined the absolute configuration of Leu and Ala as a d-configuration, while Phe was found to be present in an l-configuration. As in **1**, the absolute configuration of the Trp residue in **2** was determined by a chemical derivatization to aspartic acid,^21^ and the obtained results revealed its presence in a d-configuration. In conclusion, compound **2** was unambiguously determined to be a previously undescribed acyclic tetrapeptide of Ac-d-Trp-d-Leu-d-Ala-l-Phe and was trivially named laburnicotide B.

Compound **3** was obtained as a pale-brown, amorphous solid. The HR-ESI-MS spectrum of **3** revealed two molecular ion peaks assigned as a protonated molecule and a sodium adduct at *m*/*z* 578.2970 [M + H]^+^ (calculated 578.2973) and 600.2789 [M + Na]^+^ (calculated 600.2793), respectively. Accordingly, its molecular formula was established as C_31_H_39_N_5_O_6_ identical to that of laburnicotide B (**2**) and indicating 15 degrees of unsaturation. Despite their identical molecular formulas, compounds **2** and **3** revealed different retention times on their analytical and preparative HPLC separations (see Supporting Information Figures S9/S10–S19/S20) suggesting being different compounds. A comparison of the ^1^H NMR spectral data of **2** and **3** in DMSO-*d*_6_ (Tables 1 and 2) revealed a close coherence of those values assigned to Trp and Phe residues, while the ^1^H NMR data of Leu and Ala residues disclosed clear differences suggesting a different sequence compared to **2**. This assumption was further confirmed by the HMBC spectrum (Figure 1) that revealed common key correlations from H-2 at δ_H_ 4.53 (td, *J* = 8.7, 4.9 Hz, αH-Trp) to four different carbon atoms, namely, C-1 (δ_C_ 171.3), C-3 (δ_C_ 27.5), C-4 (δ_C_ 110.1), and C-13 (δ_C_ 169.1), while the latter revealed key correlation to a singlet methyl at δ_H_ 1.75 (s, H_3_-14; δ_C_ 22.3) indicating its presence as a *N*-acetyl terminus connected to the Trp residue similar to **1** and **2**. In addition, the HMBC spectrum revealed key correlations from a proton signal at δ_H_ 3.98 (q, *J* = 5.6 Hz, αH-Phe) to one carbonyl carbon at δ_C_ 170.4 (C-1″) and one terminal carboxylic acid carbon at δ_C_ 172.4 (C-1‴). Based on the obtained results, compound **3** was elucidated as an acyclic Ac-Trp-Ala-Leu-Phe. The absolute configuration of Ala-Leu-Phe residues in **3** was determined using Marfey’s method.^20^ The obtained results (Figures S53 and S55) unambiguously determined the absolute configurations of amino acid residues as d-Ala-d-Leu-l-Phe. Based on the common biosynthetic origin and due to the limited amount obtained that hindered performing chemical derivatization of Trp, its absolute configuration was deduced to be a d-configuration similar to **1** and **2**. Hence, compound **3** was identified as the previously undescribed acyclic tetrapeptide Ac-d-Trp-d-Ala-d-Leu-l-Phe named laburnicotide C.

**Table 2:** ^1^H and ^13^C NMR Data of **3** and **4**

	**3**		**4**	
position	δ_C_,^a^ type	δ_H_^a^ (multi, *J* [Hz])	δ_C_,^a^ type	δ_H_^a^ (multi, *J* [Hz])
Trp				
1	171.3, CO		171.3, CO	
2	53.0, CH	4.53 td (8.7, 4.9)	53.2, CH	4.53 td (8.6, 5.1)
3	27.5, CH_2_	α 2.87 dd (14.8, 8.7)	27.7, CH_2_	α 2.88 dd (overlapped)
		β 3.09 dd (14.8, 4.9)		β 3.11 dd (14.8, 4.9)
4	110.1, C		110.1, C	
5	127.3, C		127.4, C	
6	118.2, CH	7.60 d (8.1)	118.5, CH	7.60 dd (7.9, 1.2)
7	117.9, CH	6.95 t (7.5)	118.1, CH	6.95 ddd, (7.9, 6.9, 1.0)
8	120.5, CH	7.04 td (8.0, 1.0)	120.7, CH	7.04 ddd (8.1, 6.9, 1.2)
9	111.1, CH	7.31 d (8.1)	111.3, CH	7.31 dt (8.1, 0.9)
10	136.0, C		136.0, C	
11 (NH)	-	10.93 s	-	10.91 s
12	123.3, CH	7.08 d (2.2)	123.5, CH	7.09 d (2.0)
NH	-	8.09 dd (8.2, 3.4)	-	8.07 d (8.2)
13	169.1, CO		169.1, CO	
14	22.3, CH_3_	1.75 s	22.6, CH_3_	1.76 s
Ala/Ala^1^				
1′	171.6, CO		171.5, CO	
2′	48.0, CH	4.26 p (7.4)	48.1, CH	4.26 p (7.1)
3′	17.9, CH_3_	1.13 d (8.1)	18.2, CH_3_	1.14 d (7.1)
NH	-	8.14 d (7.4)	-	8.14 d (7.5)
Leu/Ala^2^				
1″	170.4, CO		170.6, CO	
2″	51.0, CH	4.18 q (8.3)	48.2, CH	4.20 p (7.1)
3″	40.8, CH_2_	1.30 dd (9.2, 5.6)	18.5, CH_3_	1.04 d (7.1)
4″	23.8, CH	1.44 m		
5″	21.3, CH_3_	0.76 d (6.6)		
6″	22.8, CH_3_	0.78 d (6.6)		
NH	-	7.86 d (7.9)	-	7.89 d (7.8)
Phe				
1‴	172.4, CO		172.4, CO	
2‴	55.1, CH	3.98 q (5.6)	55.1, CH	4.03 t (6.1)
3‴	37.3, CH_2_	α 2.88 overlapped	37.5, CH_2_	α 2.87 overlapped
		β 3.03 dd (13.3, 5.1)		β 3.05 dd (13.3, 4.9)
4‴	139.1, C		139.1, C	
5‴, 9‴	129.3, CH	7.10 m	129.6, CH	7.11 m
6‴, 8‴	127.3, CH	7.14 m	127.6, CH	7.16 dd (8.1, 6.8)
7‴	125.2, CH	7.09 m	125.5, CH	7.10 m
NH	-	7.36 d (7.3)	-	7.47 d (6.1)

a Measured in DMSO-*d*_6_ at 175 MHz for ^13^C and 700 MHz for ^1^H.

Compound **4** was isolated as a pale brown, amorphous solid. HR-ESI-MS established its molecular formula as C_28_H_33_N_5_O_6_ indicating 15 degrees of unsaturation. By comparing the molecular formulas of **2**/**3** to **4**, the difference was equal to 42 Da interpreted by the loss of the C_3_H_6_ moiety from the molecular formulas of **2**/**3**. The ^1^H NMR, ^1^H–^1^H COSY, and ROESY spectral data of **4** (Table 2 and Figures 1 and 2) in DMSO-*d*_6_ suggested the presence of four amino acid residues including Trp and Phe. In addition, two alanine residues (Ala^1^/Ala^2^) were identified in **4** confirmed by its ^1^H–^1^H COSY spectrum revealing two different spin systems extending from two pentet proton signals (δ_H_ 4.26/4.20, p, *J* = 7.1 Hz, αH-Ala^1^/Ala^2^) to two doublet methyl groups (δ_H_ 1.14/1.04, d, *J* = 7.1 Hz, βH_3_–Ala^1^/Ala^2^) and two exchangeable NH protons at δ_H_ 8.14 (d, *J* = 7.5 Hz, NH-Ala^1^) and δ_H_ 7.89 (d, *J* = 7.8 Hz, NH-Ala^2^), respectively. To determine the amino acid sequence in **4**, its HMBC spectrum (Figure 1) revealed key correlations from H-2 at δ_H_ 4.53 (td, *J* = 8.6, 5.1 Hz, αH-Trp) to four carbon atoms at δ_C_ 171.3 (C-1), 27.7 (C-3), 110.1 (C-4), and 169.1 (C-13). The latter revealed key HMBC correlation to a singlet methyl at δ_H_ 1.76 (s, H_3_-14; δ_C_ 22.6) indicating its presence as a *N*-acetyl terminus connected to the Trp residue. The HMBC spectrum of **4** (Figure 1) revealed key correlations from a proton signal at δ_H_ 4.03 (t, *J* = 6.1 Hz, H-2‴) assigned as αH-Phe to four carbon atoms at δ_C_ 170.6 (C-1″), 37.5 (C-3‴), 139.1 (C-4‴), and a terminal carboxylic acid at δ_C_ 172.4 (C-1‴). As for compounds **1**–**3**, the absolute configuration of amino acid residues in **4** was determined by chemical derivatization of Trp and Marfey’s method for other amino acid residues.^20,21^ The comparison of retention times disclosed by the authentic amino acid standards and the hydrolysate of **4** (Figures S54, S55, and S57) unambiguously determined the absolute configuration of amino acid residues as an l-configuration for Phe and a d-configuration for Trp, Ala^1^, and Ala^2^. Based on the obtained results, compound **4** was identified as a previously undescribed acyclic tetrapeptide Ac-d-Trp-d-Ala^1^-d-Ala^2^-l-Phe that was named laburnicotide D.

Compound **5** was purified as a white, amorphous solid. The molecular formula was established as C_22_H_30_N_4_O_5_ indicating 10 degrees of unsaturation using HR-ESI-MS that revealed a protonated molecular ion peak and a sodium adduct at *m*/*z* 431.2288 [M + H]^+^ (calculated 431.2289) and 453.2107 [M + Na]^+^ (calculated 453.2108), respectively. The ^1^H NMR, ^1^H–^1^H COSY, and HMBC spectral data of **5** (Table 3 and Figure 1) revealed the presence of three amino acid residues recognized as Trp, Leu, and Ala, while the comparable peaks of Phe in laburnicotides A–D (**1**–**4**) disappeared. These results suggested a tripeptide nature for **5** excluding Phe that explained its lesser five degrees of unsaturation compared to **1**–**4**. The HMBC spectrum of **5** (Figure 1) revealed key correlations from H-2 at δ_H_ 4.55 (ddd, *J* = 9.8, 8.3, 4.4 Hz, αH-Trp) to four carbon atoms ascribed to C-1 (δ_C_ 171.7), C-3 (δ_C_ 27.6), C-4 (δ_C_ 110.4), and C-13 (δ_C_ 168.8). The latter (C-13) revealed a key correlation to a singlet methyl at δ_H_ 1.74 (s, H_3_-14; δ_C_ 22.3) indicating its presence as a *N*-acetyl terminus connected to the Trp residue. In addition, the HMBC spectrum (Figure 1) unraveled key correlations from H-2″ (δ_H_ 3.62, m) assigned as an α-proton of Ala to C-1′ (δ_C_ 170.2) and a terminal carboxylic acid carbon at δ_C_ 173.5 (C-1″), whereas H-2′ (δ_H_ 4.18, td, *J* = 9.3, 5.3 Hz) assigned as an α-proton of Leu revealed key correlations to C-1 and C-1′. The obtained results suggested the chemical structure of **5** as an acyclic *N*-acetyl tripeptide Ac-Trp-Leu-Ala. The absolute configuration of Leu and Ala residues was determined by Marfey’s method.^20^ By comparing the retention times revealed by authentic amino acid standards to those exhibited by the hydrolysate of **5** (Figures S53–S56), the absolute configuration of both Leu and Ala residues was determined as a d-configuration. Based on the common biosynthetic origin and due to the shortage of the isolated amounts, compound **5** was deduced to include d-Trp in its structure similar to compounds (**1**, **2**, and **4**). Accordingly, compound **5** was identified as a previously undescribed acyclic tripeptide Ac-d-Trp-d-Leu-d-Ala and was named as laburnicotide E.

**Table 3:** ^1^H and ^13^C NMR Data of **5** and **6**

	**5**		**6**		
position	δ_C_,^a^ type	δ_H_^a^ (multi, *J* [Hz])	δ_C_,^b^ type	δ_H_^b^ (multi, *J* [Hz])	δ_H_^a^ (multi, *J* [Hz])
Trp					
1	171.7, CO		173.8, CO		
2	53.1, CH	4.55 ddd (9.8, 8.3, 4.4)	55.6, CH	4.69 td (8.0, 5.7)	4.54 ddd (10.0, 8.4, 4.3)
3	27.6, CH_2_	α 2.87 dd (14.9, 10.0)	28.9, CH_2_	α 3.12 dd (14.8, 8.0)	α 2.88 dd (14.9, 10.0)
		β 3.22 dd (14.9, 4.3)		β 3.31 dd (14.8, 5.7)	β 3.23 dd (14.9, 4.3)
4	110.4, C		111.0, C		
5	127.3, C		128.9, C		
6	118.6, CH	7.69 d (7.9)	119.4, CH	7.62 d (7.8)	7.70 d (7.8)
7	117.8, CH	6.96 ddd (7.9, 6.9, 1.0)	119.8, CH	7.00 td (7.4, 1.0)	6.96 t (7.4)
8	120.5, CH	7.04 ddd (8.1, 6.9, 1.2)	122.4, CH	7.07 ddd (8.1, 6.9, 1.1)	7.04 t (7.5)
9	110.9, CH	7.30 dt (8.1, 1.0)	112.2, CH	7.31 d (8.1)	7.30 d (8.1)
10	135.9, C		138.0, C		
11 (NH)	-	10.77 s	-	-	10.77 d (2.3)
12	123.4, CH	7.15 d (2.1)	124.6, CH	7.12 s	7.15 d (2.3)
NH	-	8.06 d (8.0)	-	-	8.04 d (8.4)
13	168.8, CO		173.2, CO		
14	22.3, CH_3_	1.74 s	22.5, CH_3_	1.90 s	1.74 s
Leu/Ala^1^					
1′	170.2, CO		173.5, CO		
2′	51.2, CH	4.18 td (9.3, 5.3)	50.5, CH	4.36 p (7.1)	4.18 p (7.2)
3′	40.4, CH_2_	α 1.47 m	18.0, CH_3_	1.30 d (7.1)	1.22 d (7.2)
		β 1.52 m			
4′	23.9, CH	1.60 tdd (13.4, 9.4, 6.1)			
5′	21.2, CH_3_	0.81 d (6.5)			
6′	22.9, CH_3_	0.88 d (6.5)			
NH	-	8.34 d (8.1)	-	-	8.37 d (7.5)
Ala/Ala^2^					
1″	173.5, CO		178.9, CO		
2″	49.7, CH	3.62 m	51.5, CH	4.19 p (7.1)	3.57 p (6.8)
3″	18.9, CH_3_	1.15 d (6.8)	19.1, CH_3_	1.34 d (7.1)	1.14 d (6.8)
NH	-	7.59 d (5.8)	-	-	7.58 d (5.8)

a Measured in DMSO-*d*_6_ at 175 MHz for ^13^C and 700 MHz for ^1^H.

b Measured in methanol-*d*_4_ at 175 MHz for ^13^C and 700 MHz for ^1^H.

Compound **6** was obtained as a white, amorphous solid. HR-ESI-MS determined its molecular formula as C_19_H_24_N_4_O_5_ indicating 10 degrees of unsaturation equal to those in **5** while having a lower molar mass of 42 Da interpreted as a difference of C_3_H_6_ between their molecular formulas. The ^1^H NMR, ^1^H–^1^H COSY, and HMBC spectra of **6** (Table 3 and Figure 1) revealed characteristic signals and key correlations indicating the presence of three amino acid residues identified as Trp, Ala^1^, and Ala^2^. As in compounds **1**–**5**, the HMBC spectrum of **6** (Figure 1) revealed characteristic correlations from a proton signal at δ_H_ 4.69 (td, *J* = 8.0, 5.7 Hz, αH-Trp) to four carbon atoms at δ_C_ 173.8, 173.2, 111.0, and 28.9 assigned to C-1, C-13, C-4, and C-3, respectively, with C-13 revealing a key correlation to a singlet methyl group at δ_H_ 1.90 (s, H_3_-14; δ_C_ 22.5) indicating its presence as a *N*-acetyl terminus connected to the Trp residue.

The ^1^H–^1^H COSY spectrum of **6** (Figure 1 and Figure S45) disclosed the presence of two different spin systems extending from two α-proton atoms (δ_H_ 4.36/4.19, p, *J* = 7.1 Hz, H-2′/2″) to two β doublet methyl groups (δ_H_ 1.30/1.34 p, *J* = 7.1 Hz, H_3_-3′/3″) suggesting the presence of two alanine residues (Ala^1^/Ala^2^). The HMBC spectrum of **6** (Figure 1) revealed key correlation from H-2′, assigned as an α-proton of Ala^2^, to a carboxylic acid carbon at δ_C_ 178.9 (C-1″) indicating its attachment to a free carboxylic acid terminus. The absolute configuration of the three amino acid residues in **6** was determined using the chemical derivatization of Trp to aspartic acid and by Marfey’s method for Ala^1^/Ala^2^.^20,21^ By comparing the obtained retention times of authentic amino acid standards to those in the hydrolysate of **6** (Figures S56 and S57), their absolute configuration was unambiguously determined as a d-configuration for three of them. A literature search of **6** revealed a closely related but synthetic peptide, featuring l- rather than d-configured amino acids, used to mimic the carbohydrate antigens in adenocarcinoma cells for inducing an immune response that ultimately kills the breast cancer cells.^22^ According to the aforementioned results, compound **6** was identified as a previously undescribed acyclic tripeptide Ac-d-Trp-d-Ala^1^-d-Ala^2^ and was named laburnicotide F.

### Biological Activity of Compounds **1**–**6**

According to our main research aim to find out new anti-infective and/or biocontrol agents, all isolated compounds were subjected to cytotoxic, antimicrobial, antiviral, and nematicidal activity assays following the previously reported protocols.^16,19,23,24^ However, the obtained results (Tables S6–S8) revealed that none of the tested compounds revealed activity in any of the conducted assays. Despite that, these negative results encouraged us to exploit the reported literature discussing oligopeptides for finding related derivatives with potential bioactivities. Oligopeptides, by definition consisting of 2 up to 10 amino acids,^25^ possess a major advantage over long-chain ones of having small molecular sizes that leads to a complete intact absorption in the intestine and hence increasing their bioavailability.^25,26^ Thus, it makes them a favorable candidate for developing functional foods as well as medicines.^27^ Several oligopeptides have been reported to exhibit antioxidative^28^ and antimicrobial activities.^29^ Acetylated oligopeptides have demonstrated protective effects against iron-overload testicular damage.^25^ By 2020, approximately 15,000 oligopeptides have been identified from 2,200 different biological species.^30^ However, their specific functions remain insufficiently explored, underscoring the importance of further research to uncover possible activities. Medically important small peptides include the angiotensin-converting enzyme (ACE) inhibitors that are considered as essential elements in the treatment regimens of hypertension and other cardiovascular disorders.^27,31^ Interestingly and to the best of our knowledge, several acyclic *N*-acetyl oligopeptides that were reported as synthetic chemicals, some revealing structural resemblance to laburnicotides A–F, exhibited potent activities as selective endothelin-1 (ET-1) receptor antagonists.^32,33^ ET-1 is identified among a class of peptides with 10-fold superior activity compared to angiotensin II, an intrinsic vasoconstrictor with long-lasting pressor effects.^34^ The ET_A_ receptor subtype is widely distributed in the vascular smooth muscle of cardio- and cerebrovascular origins, which might give it an etiological role in hypertension and its complications.

## Experimental Section

### General Experimental Procedures

Optical rotation values were measured at 20 °C using an Anton Paar MCP-150 polarimeter (Anton-Paar Opto Tec GmbH, Seelze, Germany). UV–vis and ECD spectra were conducted using a Shimadzu UV–vis spectrophotometer UV-2450 (Shimadzu, Kyoto, Japan) and a Jasco J-815 spectropolarimeter (JASCO, Pfungstadt, Germany), respectively. The HPLC-DAD-MS analysis utilized an amaZon speed ETD ion-trap mass spectrometer (Bruker Daltonics, Bremen, Germany) operating on both positive and negative ionization modes. Solutions of crude extracts and isolated pure compounds were prepared at concentrations of 4.5 and 1 mg mL^–1^, respectively. The chromatographic separation was achieved using a Dionex UltiMate 3000 UHPLC system (Thermo Scientific, Inc., Waltman, MA, USA) connected to a C_18_ Acquity UPLC BEH column (50 × 2.1 mm, 1.7 μm by Waters, MA, USA). The analysis conditions included solvent A: deionized H_2_O + 0.1% formic acid and solvent B: acetonitrile (MeCN) + 0.1% formic acid, employing a gradient of 5% B initially for 0.5 min, then ascended to 100% B over 19.5 min, and held at 100% B for an additional 5 min, with a flow rate of 0.6 mL min^–1^ and UV–vis detection spanning 190–600 nm. For HR-ESI-MS, a maXis ESI-TOF mass spectrometer (Bruker Daltonics, Bremen, Germany) integrated with an Agilent 1200 Infinity Series HPLC-UV system (Agilent Technologies, Santa Clara, CA, USA) applying similar column and gradient conditions to ESI-MS was used. Additional settings included a scan range from 100 to 2500 *m*/*z*, a 2 Hz rate, a capillary voltage of 4500 V, and a drying temperature of 200 °C. Compound solutions in deuterated methanol-*d*_4_ or DMSO-*d*_6_ were analyzed using NMR spectroscopy on Bruker Avance III systems at 500 or 700 MHz, and the latter is equipped with a 5 mm TCI cryoprobe.

### Fungal Material

The fungal strains 20AD (DSM 112866) and K01 (DSM 112867) of *Laburnicola nematophila* were isolated from infected eggs of the cereal cyst nematode *H. filipjevi* found in the agricultural terrain of Yozgat, Turkey, as previously described by Knapp et al.^13^ Prior to submerged cultivation, the isolates were kept on YM6.3 agar (4 g of d-glucose, 10 g of malt extract, 4 g of yeast extract, and 20 g of agar in 1 L of deionized water, pH 6.3 before autoclaving) in the dark.

### Fermentation, Extraction, and Isolation

Seed cultures of the strains 20AD and K01 were prepared by inoculating 200 mL of the Q6/2 medium (d-glucose 2.5 g L^–1^, glycerol 10 g L^–1^, and cottonseed flour 5 g L^–1^, pH 7.2) into 500 mL Erlenmeyer flasks. Each flask received five mycelium sections, each with a 25 mm^2^, grown on YM6.3 agar. These cultures were incubated at 23 °C with a shaking speed of 140 rpm in darkness. Upon achieving adequate biomass, the cultures were homogenized using an Ultra-Turrax (T25 easy clean digital, IKA) with an S25 N-25F dispersing tool at 10,000 rpm for 10 s. The resulting homogenized culture was used to inoculate further cultivations in BRFT (K_2_HPO_4_ 0.5 g L^–1^, sodium tartrate 0.5 g L^–1^, and yeast extract 1 g L^–1^; 100 mL of this solution was mixed with 28 g of brown rice and then autoclaved). For strain 20AD (DSM 112866), 12 and 8 flasks containing BRFT media were inoculated with 6 mL of the seed culture and cultivated for 4 and 6 weeks at room temperature in darkness. Postincubation, 250 mL of acetone was added to halt the cultures, which were then mixed and extracted three times as described by Wennrich et al.^16^ The resultant *n*-heptane and methanol fractions were dried and subjected to HPLC-DAD-MS analysis. Similarly, 35 and 15 flasks were set up for strain K01 (DSM 112867), following identical protocols.

The strains *Laburnicola nematophila* 20AD (DSM 112866) and K01 (DSM 112867) were cultivated on solid-state BRFT, which resulted in total methanol extracts of 2.2 and 3.8 g, respectively. The extracts were prepurified utilizing a FlashPure ID silica cartridge on a Grace Reveleris X2 flash chromatography system. Resulting fractions were further processed with a Luna C_18_(2) column (250 × 50 or 21.2 mm, 10 or 5 μm, Phenomenex, Aschaffenburg, Germany) on a Gilson PLC 2250 or Büchi Pure C-850 FlashPrep system. The purification steps led to the isolation of **1** (2.2 mg, *t*_R_ = 36.6–37.6 min) from strain K01, while **5** (1.0 mg, *t*_R_ = 28.0–28.3 min), **6** (0.7 mg, *t*_R_ = 30.0–30.5 min), **2** (1.6 mg, *t*_R_ = 38.0–38.4 min), **3** (0.6 mg, *t*_R_ = 38.5–38.9 min), and **4** (1.8 mg, *t*_R_ = 40.9–41.3 min) were purified from strain 20AD. Detailed description of purification steps and separation conditions is provided in the Supporting Information file (Figures S51 and S52 and Tables S1–S5).

#### Laburnicotide A (**1**)

Pale brown amorphous solid; [α]_D_^20^ +3.8° (*c* 0.053, MeOH); UV/vis (MeOH): λ_max_ (log ε) = 283.0 (3.7), 236.5 (4.5), 217.0 (4.4) nm; NMR data (^1^H NMR: 700 MHz, ^13^C NMR: 175 MHz, DMSO-*d*_6_), see Table 1; HR-(+)ESI-MS: *m*/*z* 620.3439 [M + H]^+^ (calcd. 620.3443 for C_34_H_46_N_5_O_6_^+^), 642.3258 [M + Na]^+^ (calcd. 642.3262 for C_34_H_45_N_5_NaO_6_^+^); *t*_R_ = 9.62 min (LC-ESI-MS). C_34_H_45_N_5_O_6_ (619.34 g/mol).

#### Laburnicotide B (**2**)

Pale brown amorphous solid; [α]_D_^20^ +15.6° (*c* 0.015, MeOH); UV/vis (MeOH): λ_max_ (log ε) = 283.0 (3.8), 236 (4.6), 219 (4.5) nm; NMR data (^1^H NMR: 700 MHz, ^13^C NMR: 175 MHz, methanol-*d*_4_, DMSO-*d*_6_), see Table 1; HR-(+)ESI-MS: *m*/*z* 578.2971 [M + H]^+^ (calcd. 578.2973 for C_31_H_40_N_5_O_6_^+^), 600.2790 [M + Na]^+^ (calcd. 600.2793 for C_31_H_39_N_5_NaO_6_^+^); *t*_R_ = 8.19 min (LC-ESI-MS). C_31_H_39_N_5_O_6_ (577.22 g/mol).

#### Laburnicotide C (**3**)

Pale brown amorphous solid; [α]_D_^20^ +15.4° (*c* 0.01, MeOH); UV/vis (MeOH): λ_max_ (log ε) = 404.5 (3.9), 279.0 (4.3), 234.5 (4.7), 215.5 (4.7) nm; NMR data (^1^H NMR, 2D NMR: 700 MHz, DMSO-*d*_6_), see Table 2; HR-(+)ESI-MS: *m*/*z* 578.2970 [M + H]^+^ (calcd. 578.2973 for C_31_H_40_N_5_O_6_^+^), 600.2789 [M + Na]^+^ (calcd. 600.2793 for C_31_H_39_N_5_NaO_6_^+^); *t*_R_ = 8.45 min (LC-ESI-MS). C_31_H_39_N_5_O_6_ (577.22 g/mol).

#### Laburnicotide D (**4**)

Pale brown amorphous solid; [α]_D_^20^ +50° (*c* 0.025, MeOH); UV/vis (MeOH): λ_max_ (log ε) = 339 (3.7), 280 (4.1), 240.5 (4.2), 210 (4.2) nm; NMR data (^1^H: 700 MHz, ^13^C NMR: 175 MHz, DMSO-*d*_6_), see Table 2; HR-(+)ESI-MS: *m*/*z* 536.2507 [M + H]^+^ (calcd. 536.2504 for C_28_H_34_N_5_O_6_^+^), 558.2326 [M + Na]^+^ (calcd. 558.2323 for C_28_H_33_N_5_NaO_6_^+^); *t*_R_ = 6.83 min (LC-ESI-MS). C_28_H_33_N_5_O_6_ (535.23 g/mol).

#### Laburnicotide E (**5**)

White amorphous solid; [α]_D_^20^ +3° (*c* 0.003, MeOH); UV/vis (MeOH): λ_max_ (log ε) = 287 (3.9), 206 (3.7) nm; NMR data (^1^H NMR: 700 MHz, ^13^C NMR: 175 MHz, methanol-*d*_4_), see Table 3; HR-(+)ESI-MS: *m*/*z* 431.2288 [M + H]^+^ (calcd. 431.2289 for C_22_H_31_N_4_O_5_^+^), 453.2107 [M + Na]^+^ (calcd. 453.2108 for C_22_H_30_N_4_NaO_5_^+^); *t*_R_ = 5.99 min (LC-ESI-MS). C_22_H_30_N_4_O_5_ (431.12 g/mol).

#### Laburnicotide F (**6**)

White amorphous solid; [α]_D_^20^+23.1° (*c* 0.08, MeOH); UV/vis (MeOH): λ_max_ (log ε) = 284 (4.1), 224 (4.2) nm; NMR data (^1^H NMR: 700 MHz, ^13^C NMR: 175 MHz, DMSO-*d*_6_, methanol-*d*_4_, DMSO-*d*_6_), see Table 3; HR-(+)ESI-MS: *m*/*z* 389.1817 [M + H]^+^ (calcd. 389.1819 for C_19_H_25_N_4_O_5_^+^), 411.1637 [M + Na]^+^ (calcd. 411.1639 for C_19_H_24_N_4_NaO_5_^+^); *t*_R_ = 4.24 min (LC-ESI-MS). C_19_H_24_N_4_O_5_ (388.11 g/mol).

### Advanced Marfey’s Analysis of Compounds **1**–**6**

Marfey’s method was carried out following the protocol by Pérez-Bonilla et al.^35^ with minor adjustments. In detail, 0.1 mg amounts of each compound and of the authentic amino acids (d, l, or a dl mixture of alanine, aspartic acid, leucine, and phenylalanine) were hydrolyzed with 500 μL of 6 N HCL at 90 °C for 18 h. Subsequently, the samples were dried and subjected to 200 μL of Milli-Q water, 20 μL of 1 M NaHCO_3_, and 100 μL of FDAA (1% dissolved in acetone). The reaction was incubated for 40 min at 40 °C, dried, dissolved in 1 mL of MeOH, and subjected to LC-MS measurement using an amaZon speed ESI-MS. The observed retention times of authentic amino acids were used to determine the l- or d-configuration (alanine, l: 5.61 min, d: 6.40 min; aspartic acid, l: 4.73 min, d: 5.01 min; leucine, l: 8.07 min, d: 9.0 min; phenylalanine, l: 8.01 min, d: 8.81 min).

### Determination of Tryptophan Configuration

To determine the tryptophan configuration, the samples underwent conversion into aspartic acid as previously described.^21^ In brief, 0.1 mg of the compound was dissolved in 200 μL of acetonitrile. A mixture of 300 μL of CHCl_3_:H_2_O (1:2), a catalytic amount of RuCl_3_ × H_2_O, and 18 equiv of NaIO_4_ was added to the peptides. The reaction occurred over 60 h at room temperature. Afterward, it was filtered, dried, and then subjected to the advanced Marfey’s method as described above.

### Biological Evaluation

The biological activity of isolated compounds (**1**, **2**, **4**, and **5**) was assessed against different fungi, bacteria, cell lines, *Caenorhabditis elegans*, and Chikungunya virus*.* The evaluation of antimicrobial efficacy was conducted through a serial dilution method to determine the minimum inhibitory concentrations (MICs) of isolated compounds against various Gram-positive bacteria (*Bacillus subtilis* (*B. subtilis*), *Mycobacterium smegmatis* (*M. smegmatis*), and *Staphylococcus aureus*), Gram-negative bacteria (*Acinetobacter baumannii* (*A. baumannii*), *Chromobacterium violaceum*, *Escherichia coli*, and *Pseudomonas aeruginosa* (*P. aeruginosa*)), and five fungal species (*Candida albicans*, *Mucor hiemalis*, *Rhodotorula glutinis*, *Schizosaccharomyces pombe*, and *Wickerhamomyces anomalus*), utilizing established methodologies.^16,20,23,24^ Methanol served as the negative control with a volume of 20 μL, while the choice of positive controls was tailored to the specific organism under investigation. For bacterial strains, oxytetracycline, ciprofloxacin, kanamycin, and gentamicin were employed as references for *B. subtilis*, *A. baumannii*, *M. smegmatis*, and *P. aeruginosa*, respectively; nystatin was the standard for all fungi. The cytotoxicity of compounds was assessed on L929 mouse fibroblast cells and KB3.1 human endocervical adenocarcinoma cells using an MTT assay. Epothilone B acted as the positive control in these experiments. Additionally, the nematicidal potential was evaluated using *Caenorhabditis elegans* in a 48-well flat-bottom plate, with compounds tested at concentrations of 100, 50, and 10 μg mL^–1^. Samples were dissolved in methanol and added to the wells and then dried using a nitrogen stream. Subsequently, 300 μL of a suspension containing 1,000 nematodes/mL was added to the samples and incubated for 18 h at 23 °C and 150 rpm. Ivermectin, at a concentration of 1 μg mL^–1^, served as the positive control, and methanol was used as the negative control. This assay was executed according to the procedures outlined by Phutthacharoen et al.^23^

### Antiviral Assay

The 2 × 10^4^ Huh 7.5.1 cells were seeded in a 96-well plate and cultured overnight for adherence. The spent culture medium was aspirated and replaced with 50 μL of compound dilutions at 50 μM in Dulbecco’s modified Eagle medium (DMEM). A 0.5% DMSO solution served as a negative control and ribavirin as the positive control. Subsequently, 50 μL of a Chikungunya wildtype (CHIKV) virus suspension (multiplicity of infection (MOI) of 0.1) was added to the respective wells. The cells were then incubated for 72 h at standard culture conditions of 37 °C and 5% CO_2_. Percentage cell viability as a measure of the antiviral effect was determined using a CellTiter-Glo (Promega) assay following the manufacturer’s recommendations.

## Supporting Information Available

All HPLC-ESI-MS, HR-ESI-MS, 1D (^1^H/^13^C), and 2D (^1^H–^1^H COSY, HMBC, HSQC, and ROESY) spectra and chromatographic workup schemes and advanced Marfey’s analysis of compounds (**1**–**6**) (PDF)
