# Supplementary material for: Laburnicotides A–F: Acyclic N-Acetyl Oligopeptides from the Nematode-Cyst-Associated Fungus Laburnicola nematophila
Source: ACS Omega. 2024 May 6;9(19):21658–67. doi: 10.1021/acsomega.4c02719 (PMC11097168; doi:10.1021/acsomega.4c02719)
Supplement: Supplementary file 1 — ao4c02719_si_001.pdf [file ao4c02719_si_001.pdf]

Supporting Information for:

Laburnicotides A–F: Acyclic *N*-acetyl Oligopeptides from the Nematode-Cyst Associated Fungus *Laburnicola nematophila*

Caren Holzenkamp,[†,‡,a] Jan-Peer Wennrich,[†,‡,a] Jackson M. Muema,[§] Samad Ashrafi,[⊥,∇] Wolfgang Maier,[⊥] Marc Stadler,[†,‡*] and Sherif S. Ebada[†,¶*]

[†] Department of Microbial Drugs, Helmholtz Centre for Infection Research (HZI) and German Centre for Infection Research, Inhoffenstrasse 7, 38124 Braunschweig, Germany.

[‡] Institute of Microbiology, Technische Universität Braunschweig, Spielmannstraße 7, 38106 Braunschweig, Germany.

[§] Compound Profiling and Screening (COPS), Helmholtz Centre for Infection Research (HZI), Inhoffenstrasse 7, 38124 Braunschweig, Germany

[⊥] Institute for Epidemiology and Pathogen Diagonstics, Julius Kühn Institute (JKI) - Federal Research Center for Cultivated Plants, Messeweg 11-12, 38104 Braunschweig, Germany.

[∇] Institute for Crop and Soil Science, Julius Kühn Institute (JKI) – Federal Research Centre for Cultivated Plants, Bundesallee 58, 38116 Braunschweig, Germany.

[¶] Department of Pharmacognosy, Faculty of Pharmacy, Ain Shams University, Cairo 11566, Egypt.

[a] The authors equally contributed to this work.

[*] Correspondence:

E-mail: sherif.elsayed@helmholtz-hzi.de, sherif_elsayed@pharma.asu.edu.eg (S.S.E); Marc.Stadler@helmholtz-hzi.de (M.S.); Tel.: +49-531-6181-424, Fax: +49-531-6181-9499

Contents of Supporting Information

#	Contents	Page
**1**	Figure S1. LR-ESI-MS spectra of **1**.	**S4**
**2**	Figure S2. HR-ESI-MS spectra of **1**.	**S5**
**3**	Figure S3. ^{1}H NMR spectrum of **1** in DMSO-d_6 at 500 MHz.	**S6**
**4**	Figure S4. ^{13}C NMR spectrum of **1** in DMSO-d_6 at 125 MHz.	**S7**
**5**	Figure S5. ^{1}H–^{1}H COSY spectrum of **1** in DMSO-d_6 at 500 MHz.	**S8**
**6**	Figure S6. HMBC spectrum of **1** in DMSO-d_6 at 500 MHz.	**S9**
**7**	Figure S7. HSQC spectrum of **1** in DMSO-d_6 at 500 MHz.	**S10**
**8**	Figure S8. ROESY spectrum of **1** in DMSO-d_6 at 500 MHz.	**S11**
**9**	Figure S9. LR-ESI-MS spectra of **2**.	**S12**
**10**	Figure S10. HR-ESI-MS spectra of **2**.	**S13**
**11**	Figure S11. ^{1}H NMR spectrum of **2** in methanol-d_4 at 500 MHz.	**S14**
**12**	Figure S12. ^{13}C NMR spectrum of **2** in methanol-d_4 at 125 MHz.	**S15**
**13**	Figure S13. ^{1}H-^{1}H COSY spectrum of **2** in methanol-d_4 at 500 MHz.	**S16**
**14**	Figure S14. HMBC spectrum of **2** in methanol-d_4 at 500 MHz.	**S17**
**15**	Figure S15. HSQC spectrum of **2** in methanol-d_4 at 500 MHz.	**S18**
**16**	Figure S16. ROESY spectrum of **2** in methanol-d_4 at 500 MHz.	**S19**
**17**	Figure S17. ^{1}H NMR spectrum of **2** in DMSO-d_6 at 700 MHz.	**S20**
**18**	Figure S18. ROESY spectrum of **2** in DMSO-d_6 at 700 MHz.	**S21**
**19**	Figure S19. LR-ESI-MS spectra of **3**.	**S22**
**20**	Figure S20. HR-ESI-MS spectra of **3**.	**S23**
**21**	Figure S21. ^{1}H NMR spectrum of **3** in DMSO-d_6 at 700 MHz.	**S24**
**22**	Figure S22. ^{1}H-^{1}H COSY spectrum of **3** in DMSO-d_6 at 700 MHz.	**S25**
**23**	Figure S23. HMBC spectrum of **3** in DMSO-d_6 at 700 MHz.	**S26**
**24**	Figure S24. HSQC spectrum of **3** in DMSO-d_6 at 700 MHz.	**S27**
**25**	Figure S25. ROESY spectrum of **2** in DMSO-d_6 at 700 MHz.	**S28**
**26**	Figure S26. LR-ESI-MS spectra of **4**.	**S29**
**27**	Figure S27. HR-ESI-MS spectra of **4**.	**S30**
**28**	Figure S28. ^{1}H NMR spectrum of **4** in DMSO-d_6 at 700 MHz.	**S31**
**29**	Figure S29. ^{13}C NMR spectrum of **4** in DMSO-d_6 at 175 MHz.	**S32**
**30**	Figure S30. ^{1}H-^{1}H COSY spectrum of **4** in DMSO-d_6 at 500 MHz.	**S33**
**31**	Figure S31. HMBC spectrum of **4** in DMSO-d_6 at 500 MHz.	**S34**
**32**	Figure S32. HSQC spectrum of **4** in DMSO-d_6 at 500 MHz.	**S35**
**33**	Figure S33. ROESY spectrum of **4** in DMSO-d_6 at 700 MHz.	**S36**
**34**	Figure S34. LR-ESI-MS spectra of **5**.	**S37**
**35**	Figure S35. HR-ESI-MS spectra of **5**.	**S38**
**36**	Figure S36. ^{1}H NMR spectrum of **5** in DMSO-d_6 at 700 MHz.	**S39**
**37**	Figure S37. ^{1}H-^{1}H COSY spectrum of **5** DMSO-d_6 at 700 MHz.	**S40**
**38**	Figure S38. HMBC spectrum of **5** in DMSO-d_6 at 700 MHz.	**S41**
**39**	Figure S39. HSQC spectrum of **5** in DMSO-d_6 at 700 MHz.	**S42**
**40**	Figure S40. ROESY spectrum of **5** in DMSO-d_6 at 700 MHz.	**S43**
**41**	Figure S41. LR-ESI-MS spectra of **6**.	**S44**
**42**	Figure S42. HR-ESI-MS spectra of **6**.	**S45**
**43**	Figure S43. ^{1}H NMR spectrum of **6** in methanol-d_4 at 700 MHz.	**S46**
**44**	Figure S44. ^{13}C NMR spectrum of **6** in methanol-d_4 at 175 MHz.	**S47**
**45**	Figure S45. ^{1}H-^{1}H COSY spectrum of **6** in methanol-d_4 at 700 MHz.	**S48**
**46**	Figure S46. HMBC spectrum of **6** in methanol-d_4 at 700 MHz.	**S49**
**47**	Figure S47. HSQC spectrum of **6** in methanol-d_4 at 700 MHz.	**S50**
**48**	Figure S48. ROESY spectrum of **6** in methanol-d_4 at 700 MHz.	**S51**
**49**	Figure S49. ^{1}H NMR spectrum of **6** in DMSO-d_6 at 700 MHz.	**S52**
**50**	Figure S50. ROESY spectrum of **6** in DMSO-d_6 at 700 MHz.	**S53**
**51**	Figure S51. Overview of purification steps to isolate compound **1** from methanol extract of *Laburnicola nematophila* Strain 20AD cultivated on BRFT media.	**S54**
**52**	Table S1. Pre-fractionation with NP-MPLC separation parameters for *Laburnicola nematophila* Strain K01 BRFT methanol extract.	**S54**

**53**	Table S2. Preparative RP-HPLC separation parameters used to isolate compound **1**.	**S54**
**54**	Figure S52. Overview of purification steps to isolate compounds (**2**–**6**) from methanol extract of *Laburnicola nematophila* Strain K01 cultivated on BRFT media.	**S55**
**55**	Table S3. Pre-fractionation with NP-MPLC separation parameters for *Laburnicola nematophila* Strain K01 BRFT methanol extract.	**S55**
**56**	Table S4. Preparative RP-HPLC separation parameters used for F9+F10 to isolate compounds (**2**, **3** and **5**).	**S56**
**57**	Table S5. Preparative RP-HPLC separation parameters used for F11 to isolate **4** and **6**.	**S56**
**58**	Figure S53. LC-ESI-MS spectra of L-leucine, DL-leucine, compounds (**1**, **2**, **3** and **5**) + FDAA. Top-bottom: L-leucine + FDAA, DL-leucine + FDAA, (**1**, **2**, **3** and **5**) + FDAA. UV-chromatogram at 190–600 nm is shown. Bars are indicating identical MS-Peaks (blue D-leucine).	**S57**
**59**	Figure S54. LC-ESI-MS spectra of DL-phenylalanine, L-phenylalanine, compounds (**1**, **2**, **3** and **4**) + FDAA. Top-bottom: DL-phenylalanine + FDAA, L-phenylalanine + FDAA, (**1**, **2**, **3** and **4**) + FDAA. UV-chromatogram at 190–600 nm is shown. Bars are indicating identical MS-Peaks (blue L-phenylalanine).	**S57**
**60**	Figure S55. LC-ESI-MS spectra of L-alanine, DL-alanine, compounds (**2**, **3** and **4**) + FDAA. Top-bottom: L-alanine + FDAA, DL-alanine + FDAA, **2-4** + FDAA. UV-chromatogram at 190–600 nm is shown. Bars are indicating identical MS-Peaks (blue D-alanine).	**S58**
**61**	Figure S56. LC-ESI-MS spectra of L-alanine, DL-alanine, compounds (**5** and **6**) + FDAA. Top-bottom: L-alanine + FDAA, DL-alanine + FDAA, **5**/**6** + FDAA. UV-chromatogram at 190–600 nm is shown. Bars are indicating identical MS-Peaks (blue D-alanine).	**S58**
**62**	Figure S57. LC-ESI-MS spectra of DL-aspartic acid, L-aspartic acid, compounds (**2**, **4** and **6**) + FDAA. Top-bottom: DL-aspartic acid + FDAA, L-aspartic acid + FDAA, (**2**, **4** and **6**) + FDAA. UV-chromatogram at 190–600 nm and is shown. Bars are indicating identical MS-Peaks (blue L-phenylalanine).	**S59**
**63**	Table S6. Cytotoxicity (IC_{50} in µM) of **1**–**6**.	**S59**
**64**	Table S7. Antimicrobial activity (MIC) of **1**–**6**.	**S60**
**65**	Table S8. Nematicidal activity of **1**, **2**, **4** and **5**.	**S60**

Display Report

Analysis Info

		Acquisition Date	07.10.2023 01:03:42
Analysis Name	S:\PEOPLE\cho23_Caren Holzenkamp\NMR\MS-Data\purified fractions\MeOH-F9-F7-619\MyNe_03_03_06_MeOH_F9_F7_RC7_01_51474.d		
Method	51474.m	Operator	tti
Sample Name	MyNe_03_03_06_MeOH_F9_F7	Instrument	amaZon speed
Comment			

Acquisition Parameter

Ion Source Type	ESI	Ion Polarity	Positive	Alternating Ion Polarity	on
Mass Range Mode	UltraScan	Scan Begin	100 m/z	Scan End	2000 m/z
Accumulation Time	4000 µs	RF Level	100 %	Trap Drive	68.9
SPS Target Mass	1000 m/z	Averages	6 Spectra		

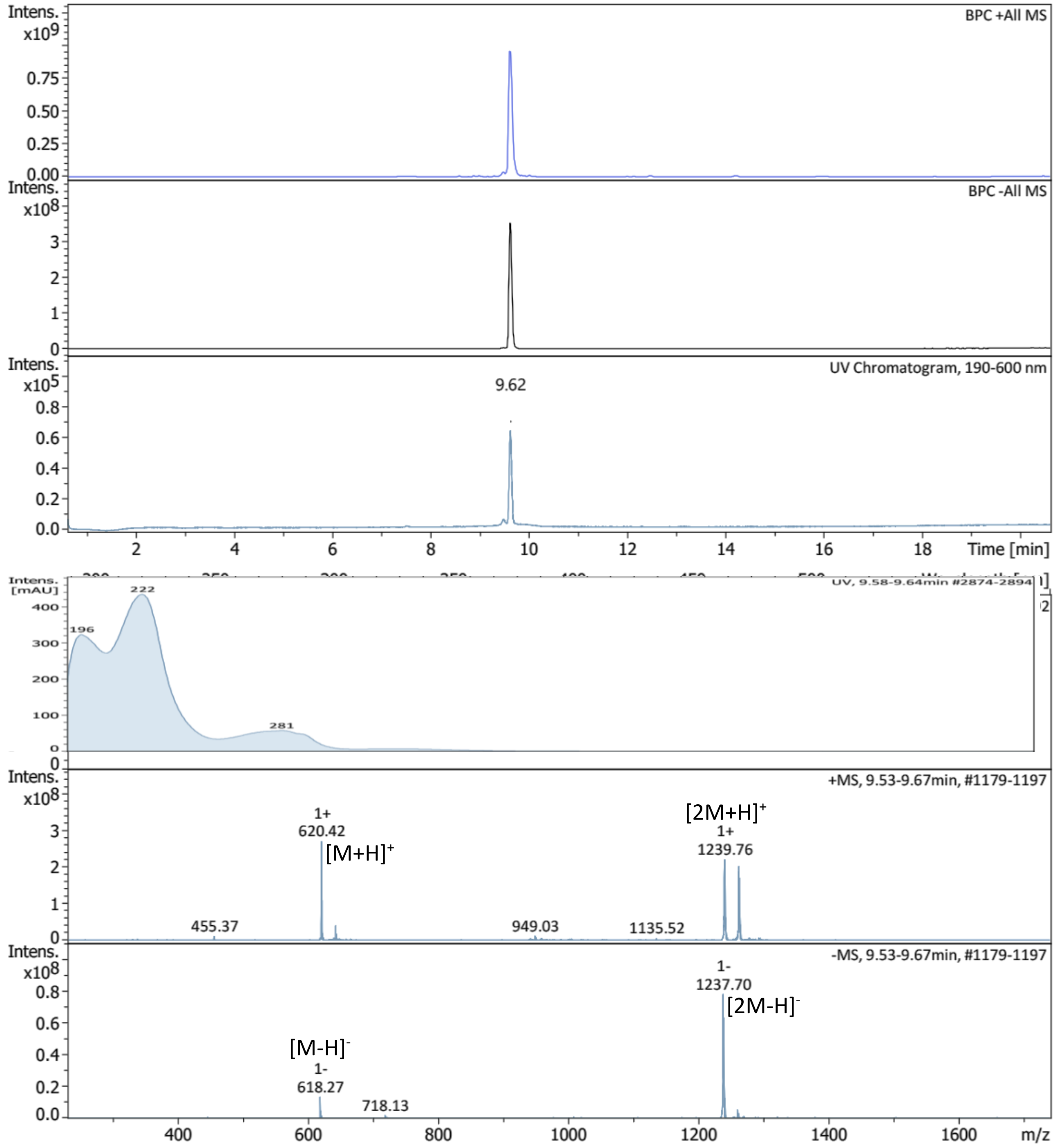

Bruker Compass DataAnalysis 6.1 printed: 03.03.2024 18:27:02 by: sel22 Page 1 of 1

Figure S1. LR-ESI-MS of **1**.

Display Report

Analysis Info

Analysis Name	S:\PEOPLE\cho23_Caren Holzenkamp\NMR\MS-Data\purified fractions\MeOH-F9-F7-619\MyNe-03-03-06-MeOH-F9-F7_P1-A-2_1_893.d	Acquisition Date	02.11.2023 22:39:21
Method	MWIS_BEH50mm_25min_ohnetims.m	Operator	Demo User
Sample Name	MyNe-03-03-06-MeOH-F9-F7	Instrument	timsTOF Pro 2
Comment			

Acquisition Parameter

Ion Polarity Positive

SPS Target Mass

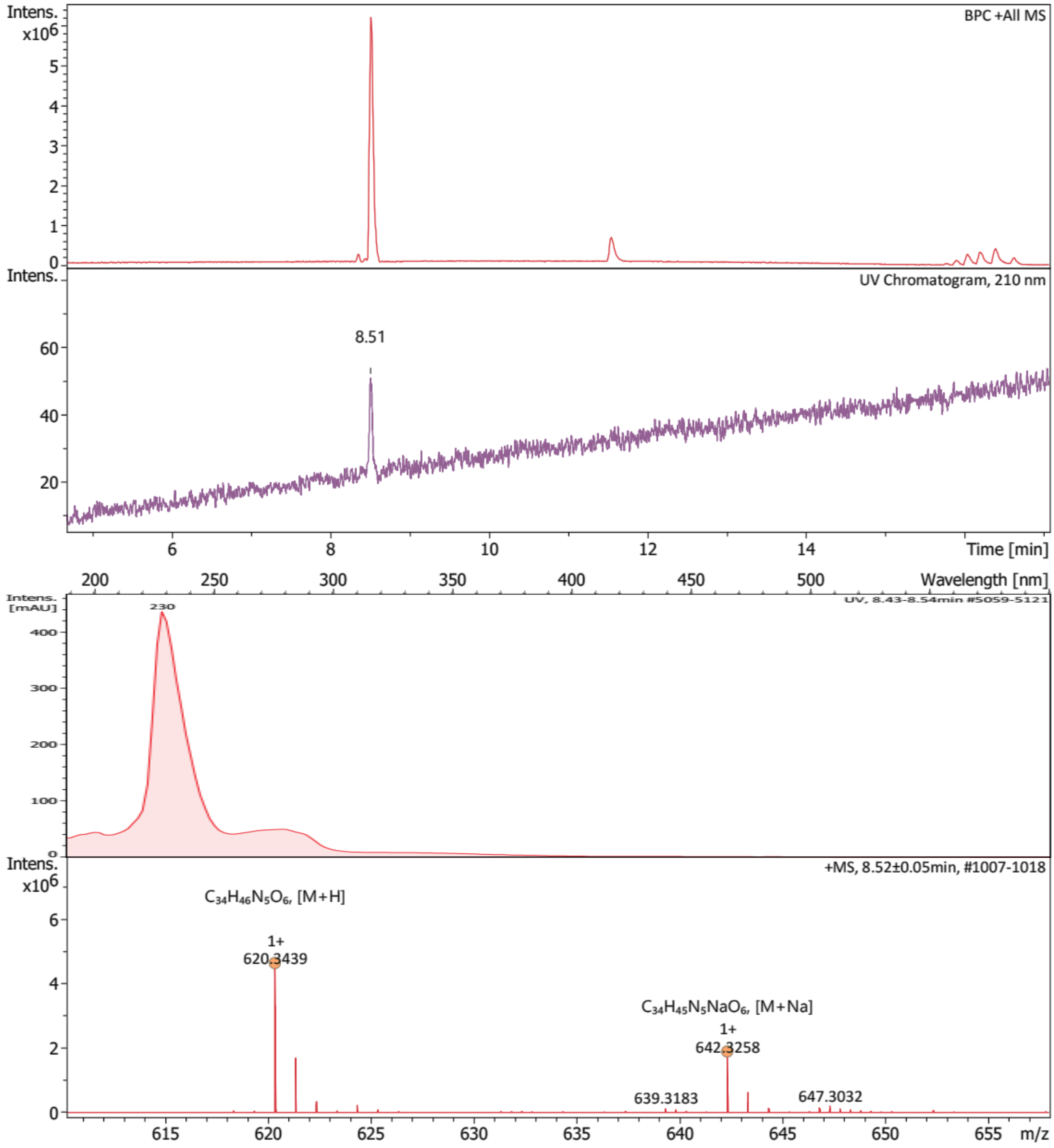

Bruker Compass DataAnalysis 6.1 printed: 06.11.2023 11:44:25 by: sel22 Page 1 of 1

Figure S2. HR-ESI-MS of **1**.

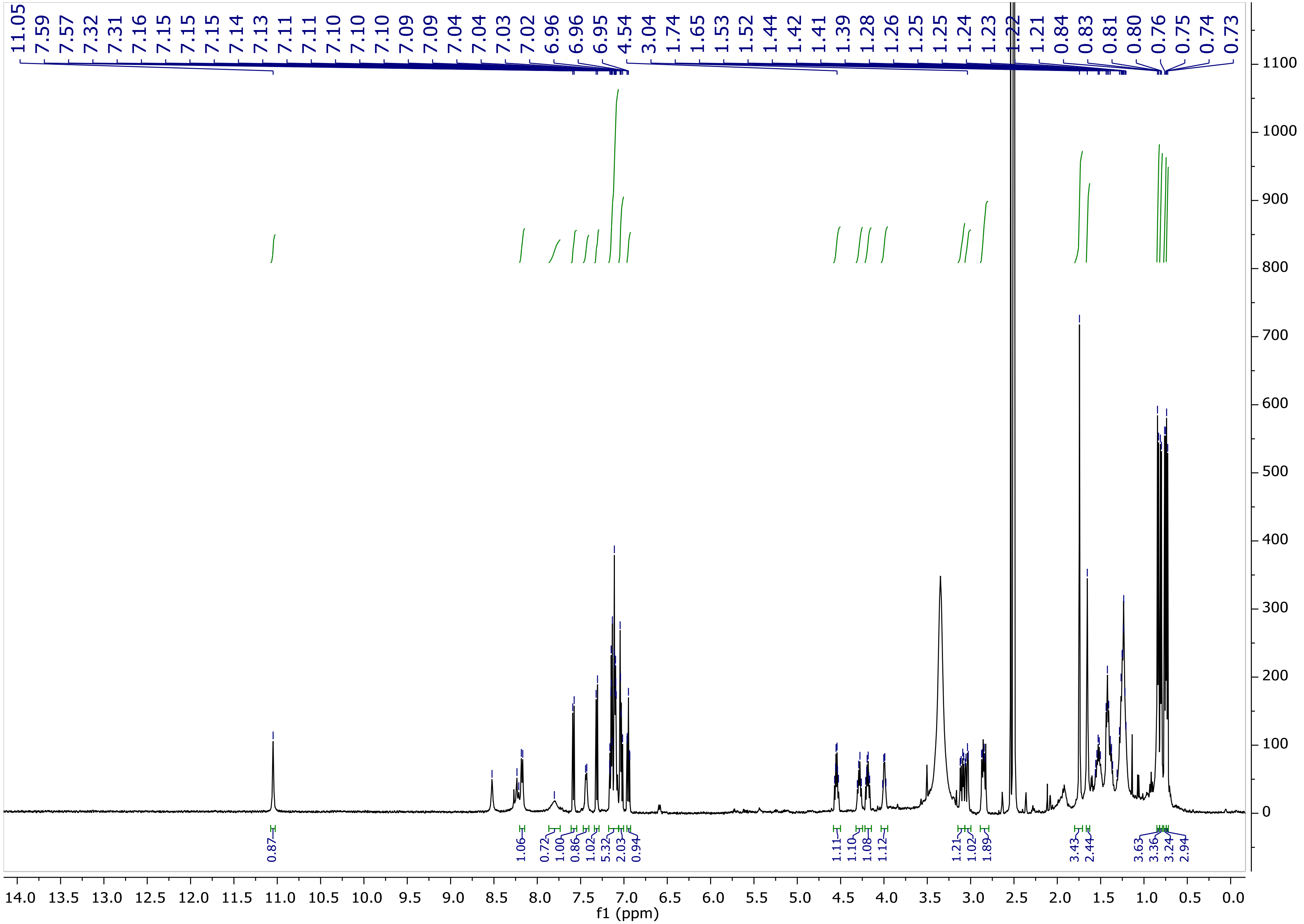

Figure S3. ^{1}H NMR spectrum of **1** in DMSO-d_6 at 500 MHz.

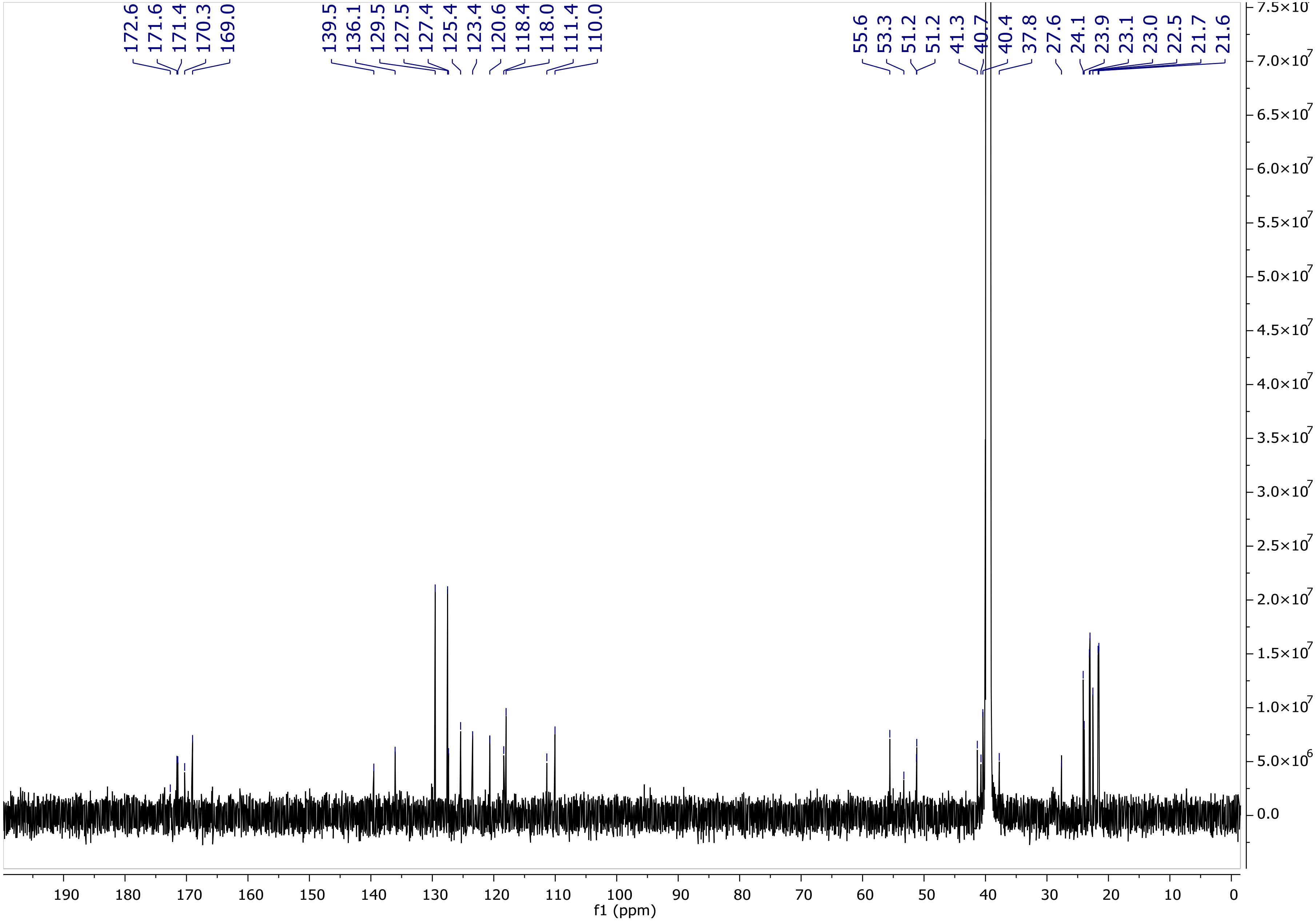

Figure S4. ^{13}C NMR spectrum of **1** in DMSO-d_6 at 125 MHz.

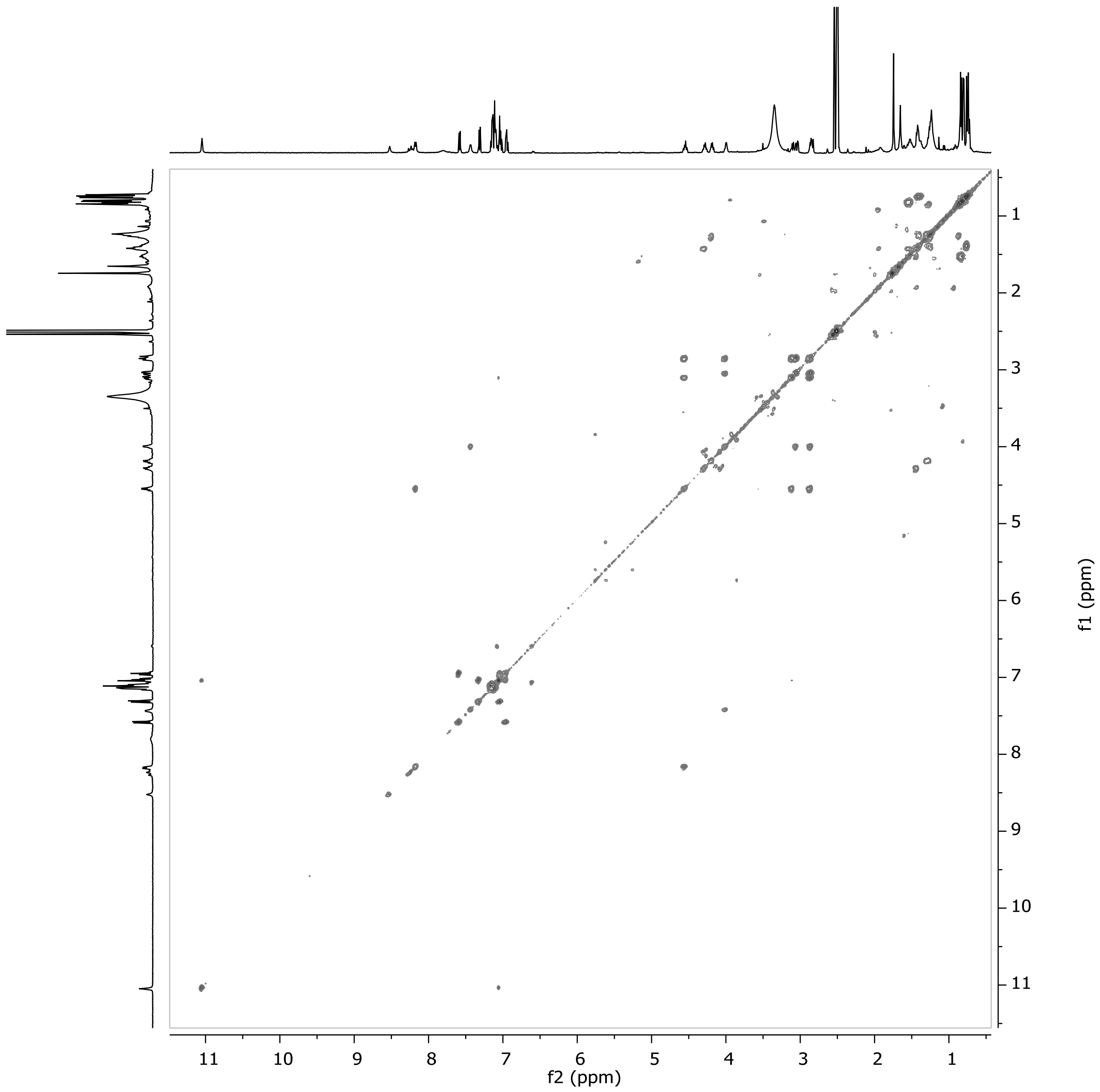

Figure S5. ^{1}H–^{1}H COSY spectrum of **1** in DMSO-d_6 at 500 MHz.

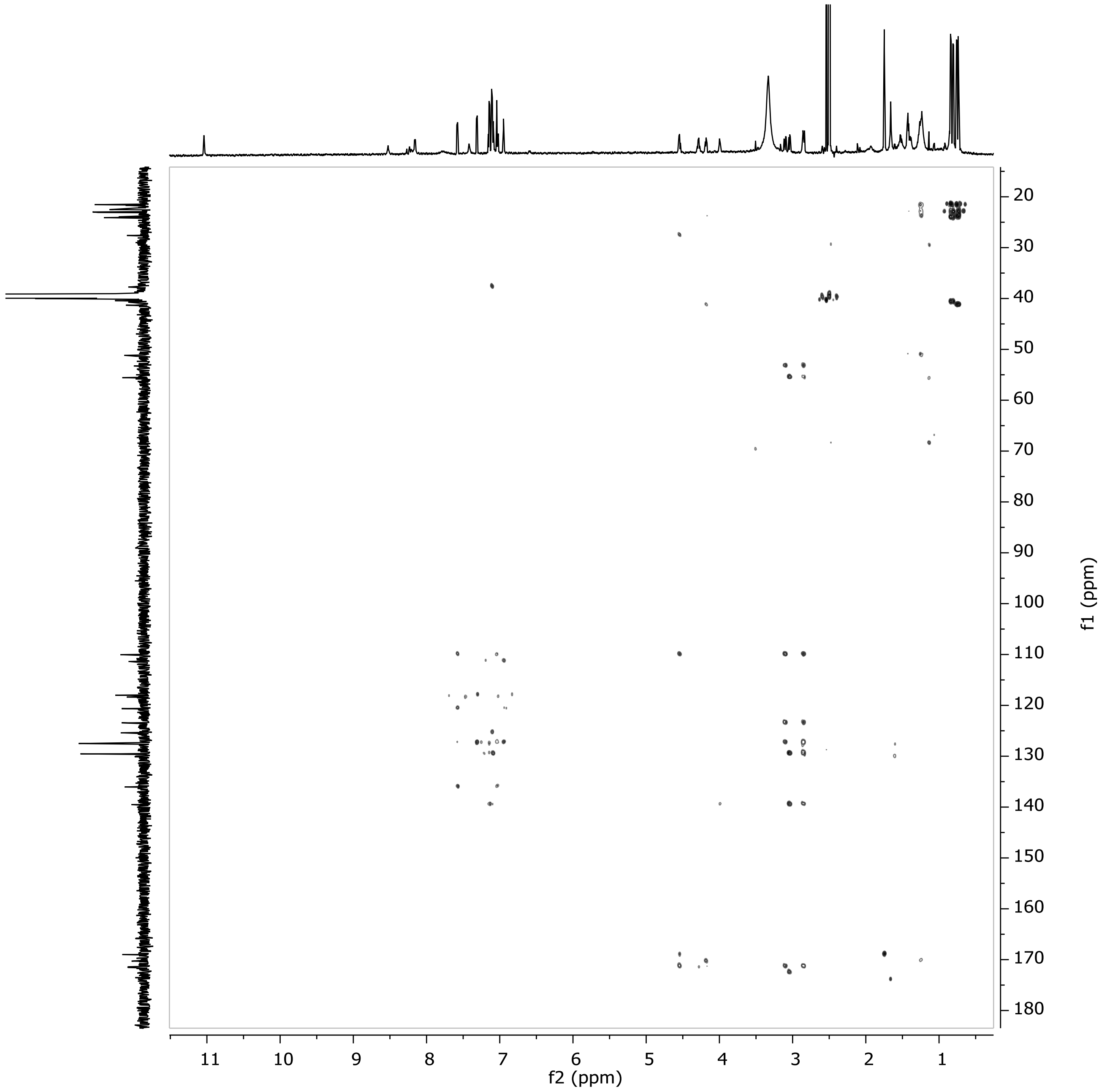

Figure S6. HMBC spectrum of **1** in DMSO-d_6 at 500 MHz.

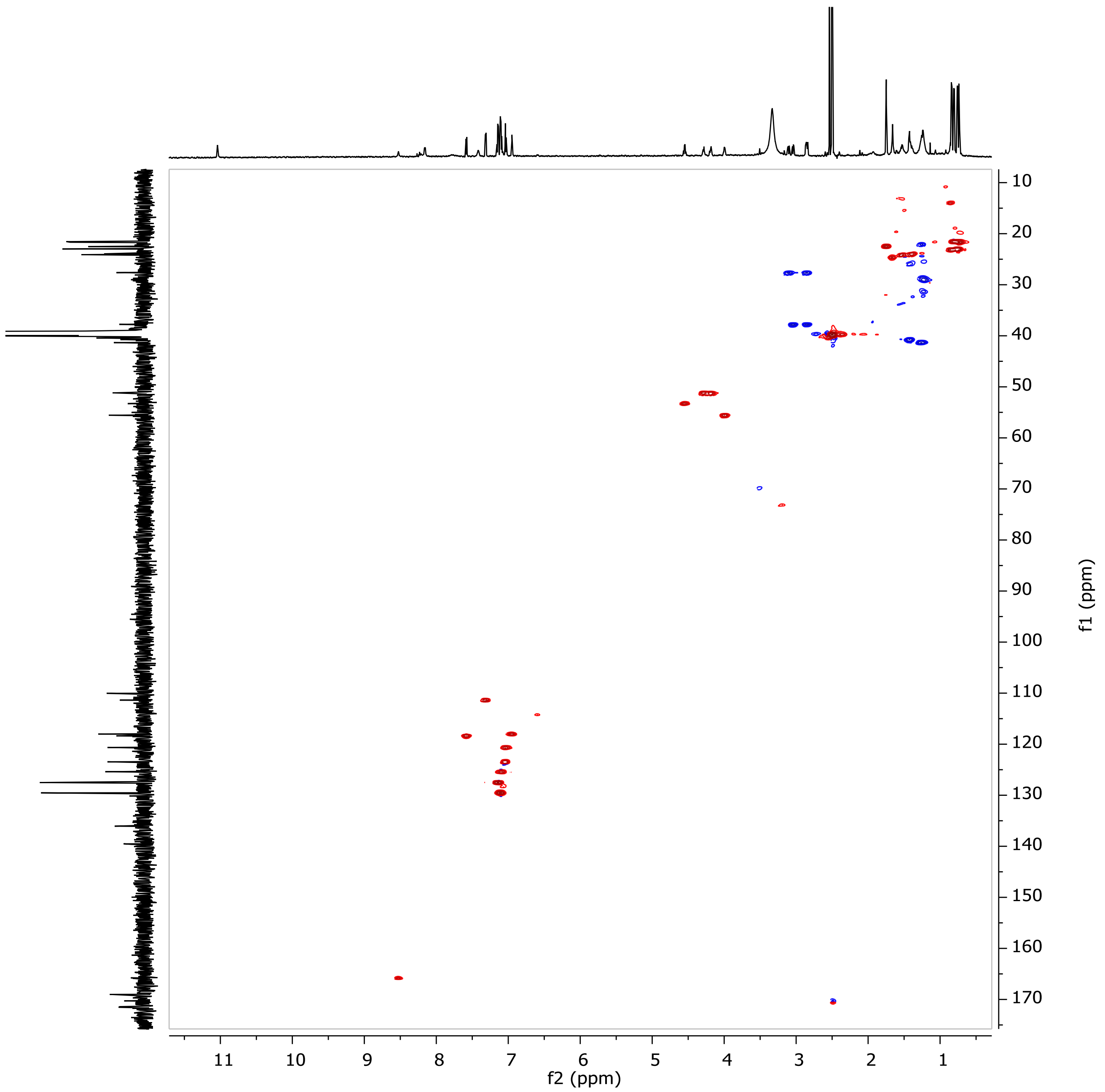

Figure S7. HSQC spectrum of **1** in DMSO-d_6 at 500 MHz.

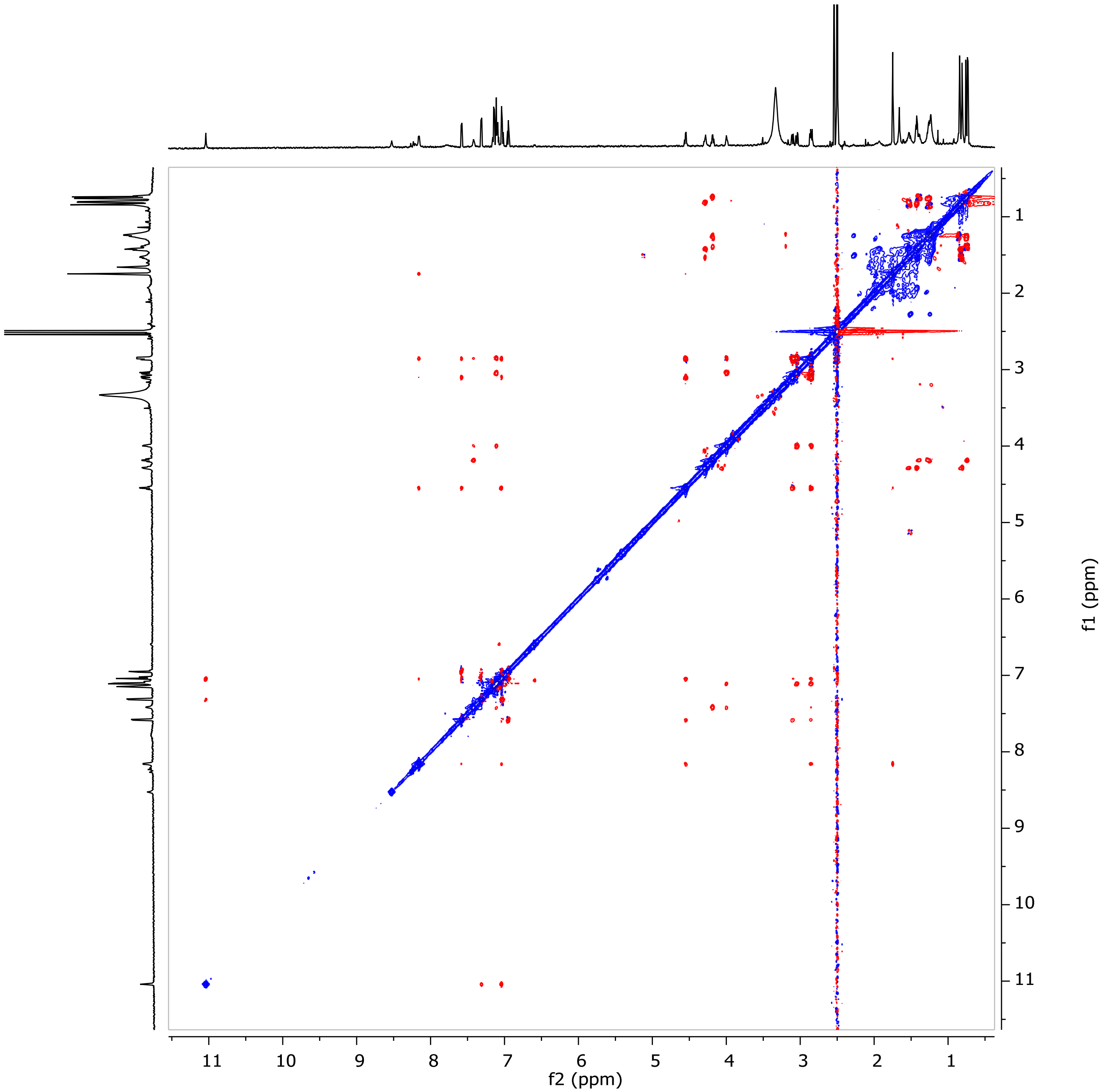

Figure S8. ROESY spectrum of **1** in DMSO-d_6 at 500 MHz.

Display Report

Analysis Info

Analysis Name	S:\PEOPLE\cho23_Caren Holzenkamp\NMR\MS-Data\purified fractions\MeOH-F9-F10-F13-577\MyNe_03_02_06_MeOH_F9+F10_F13_RD5_01_51538.d	Acquisition Date	10.10.2023 00:35:17
Method	51538.m	Operator	tti
Sample Name	MyNe_03_02_06_MeOH_F9+F10_F13	Instrument	amaZon speed
Comment			

Acquisition Parameter

Ion Source Type	ESI	Ion Polarity	Negative	Alternating Ion Polarity	on
Mass Range Mode	UltraScan	Scan Begin	100 m/z	Scan End	2000 m/z
Accumulation Time	3593 µs	RF Level	100 %	Trap Drive	78.0
SPS Target Mass	1000 m/z	Averages	6 Spectra		

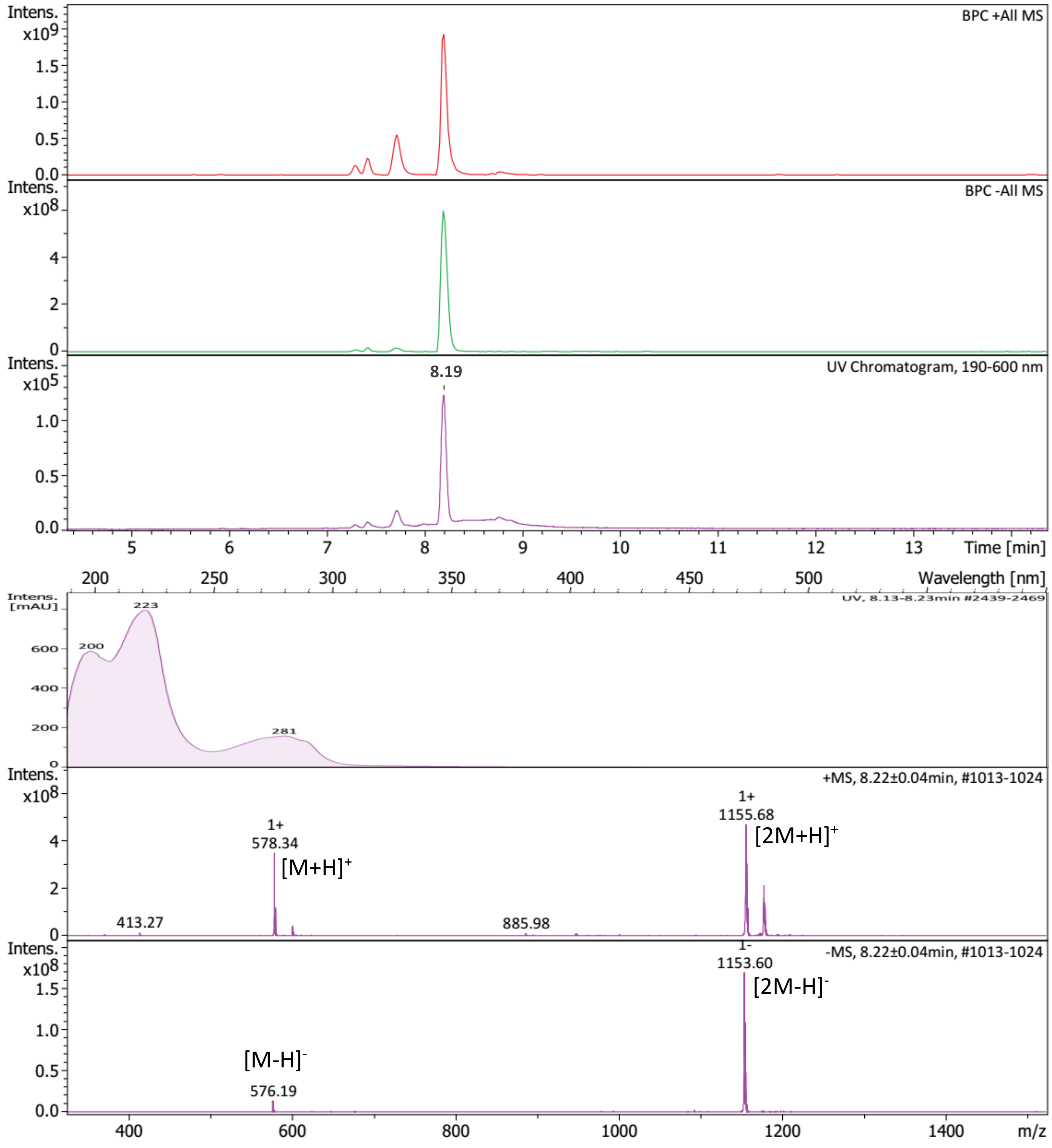

Bruker Compass DataAnalysis 6.1 printed: 27.11.2023 12:43:00 by: sel22 Page 1 of 1

Figure S9. LR-ESI-MS of **2**.

Display Report

Analysis Info

Analysis Name	S:\PEOPLE\cho23_Caren Holzenkamp\NMR\MS-Data\purified fractions\MeOH-F9-F10-F13-577\MyNe-03-02-06-MeOH-F9+F10-F13_P1-A-6_1_897.d	Acquisition Date	03.11.2023 00:43:09
Method	MWIS_BEH50mm_25min_ohnetims.m	Operator	Demo User
Sample Name	MyNe-03-02-06-MeOH-F9+F10-F13	Instrument	timsTOF Pro 2 1875087.10646
Comment			

Acquisition Parameter

Source Type	ESI	Ion Polarity	Positive	Set Nebulizer	1.0 Bar
Focus	Active	Set Capillary	4000 V	Set Dry Heater	200 °C
Scan Begin	150 m/z	Set End Plate Offset	-500 V	Set Dry Gas	5.0 l/min
Scan End	2500 m/z	Set Charging Voltage	2000 V	Set Divert Valve	Waste
		Set Corona	0 nA	Set APCI Heater	0 °C

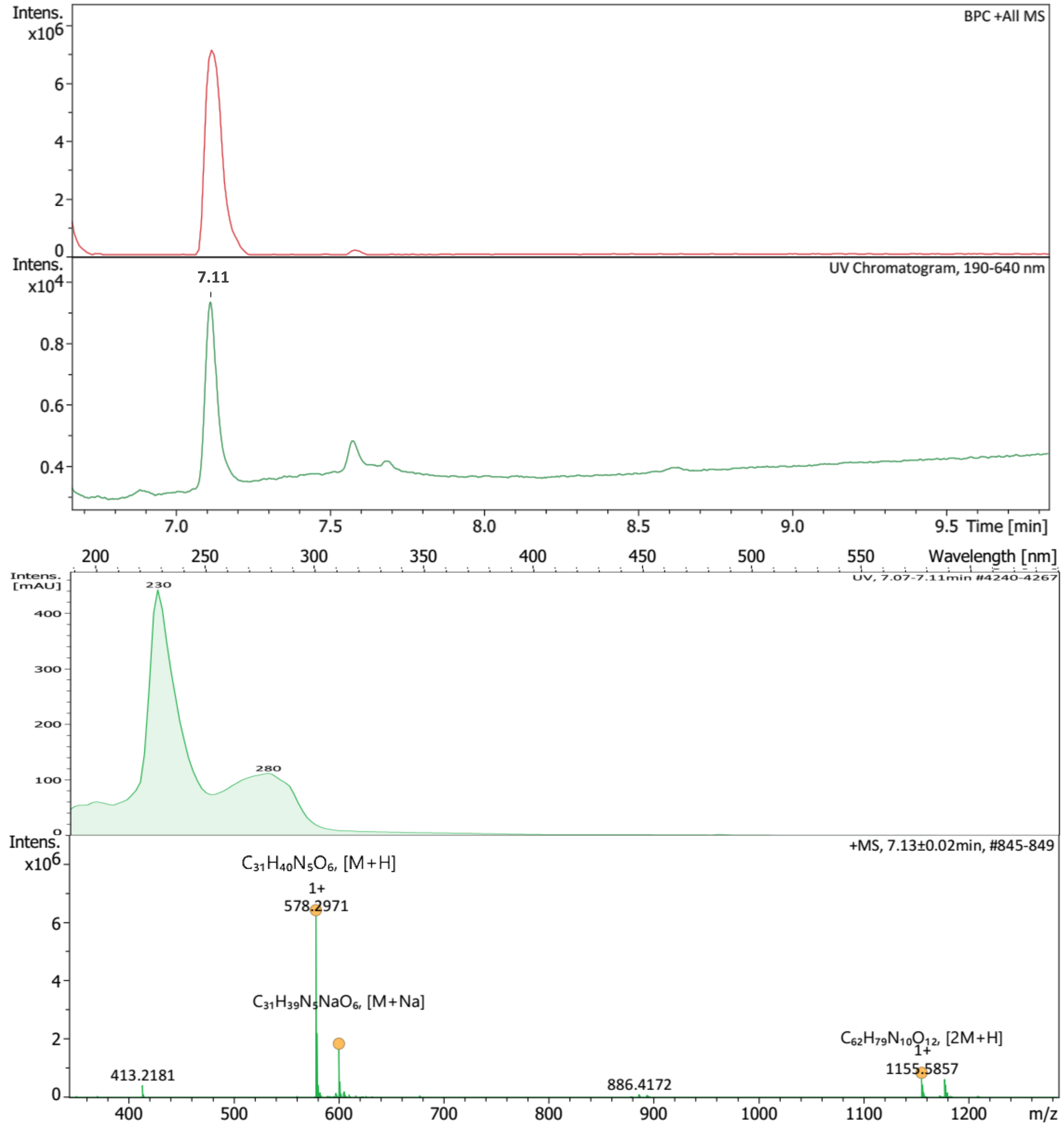

Bruker Compass DataAnalysis 6.1 printed: 27.11.2023 12:46:00 by: sel22 Page 1 of 1

Figure S10. HR-ESI-MS of **2**.

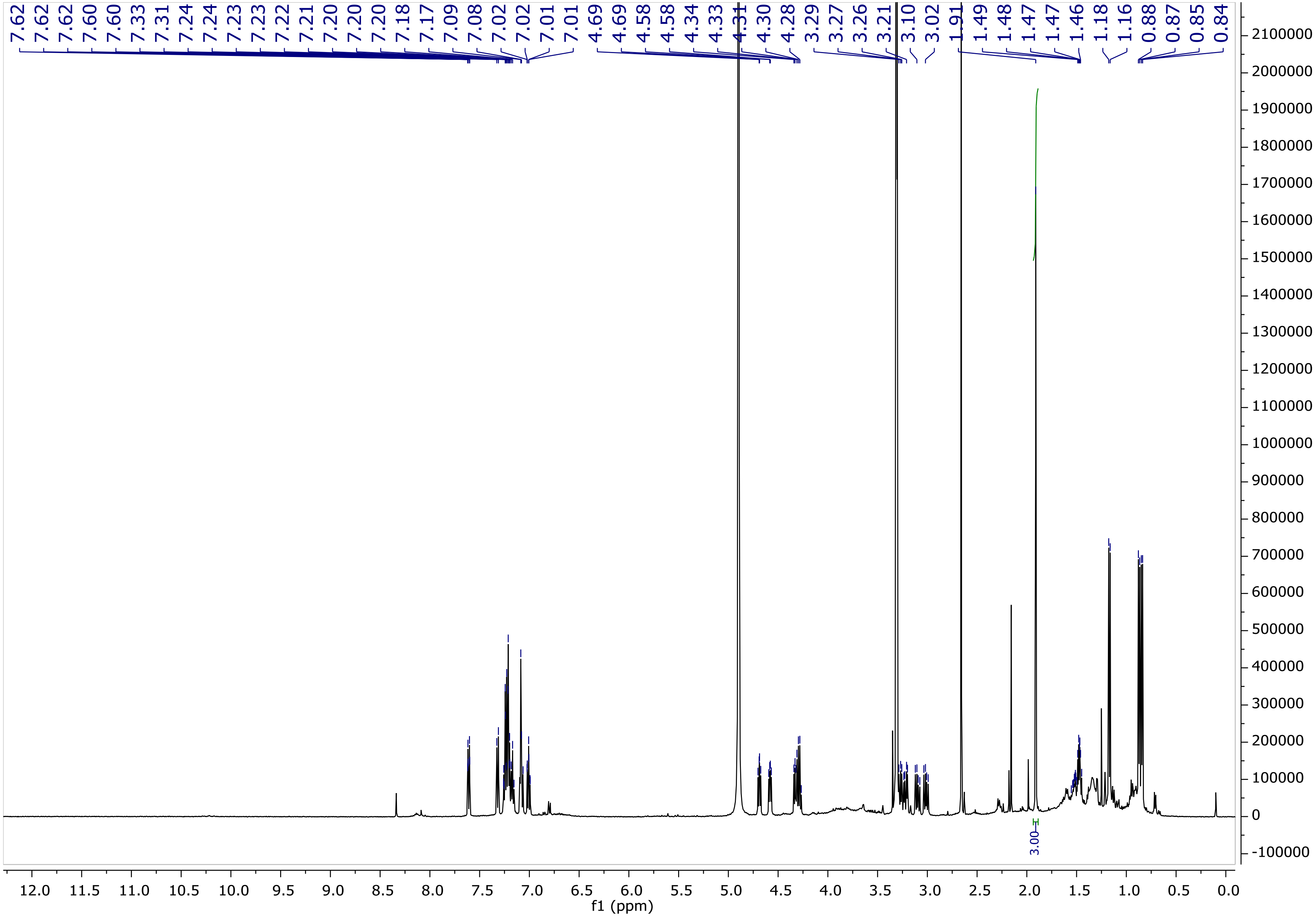

Figure S11. ^{1}H NMR spectrum of **2** in methanol-d_4 at 500 MHz.

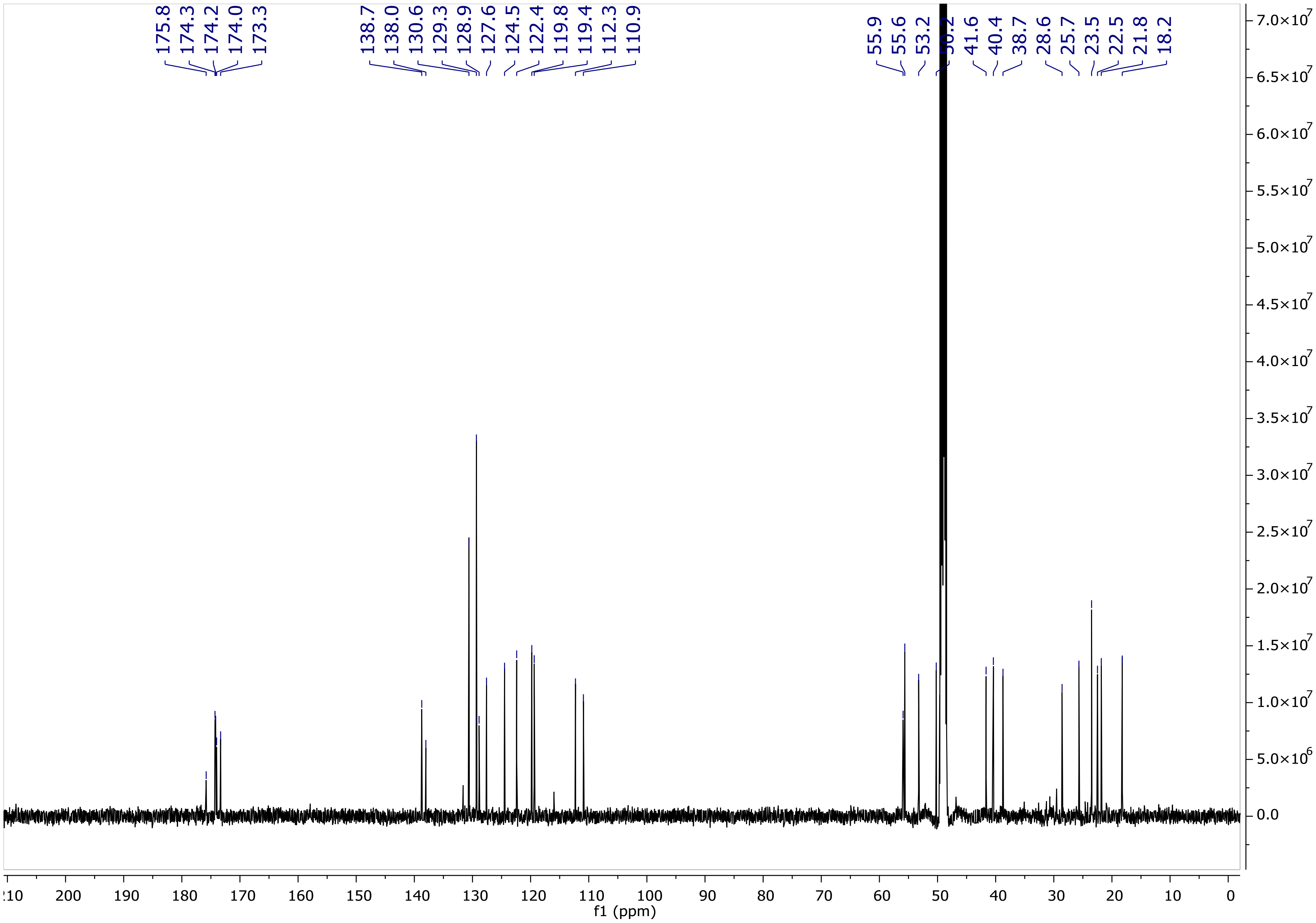

Figure S12. ^{13}C NMR spectrum of **2** in methanol-d_4 at 125 MHz.

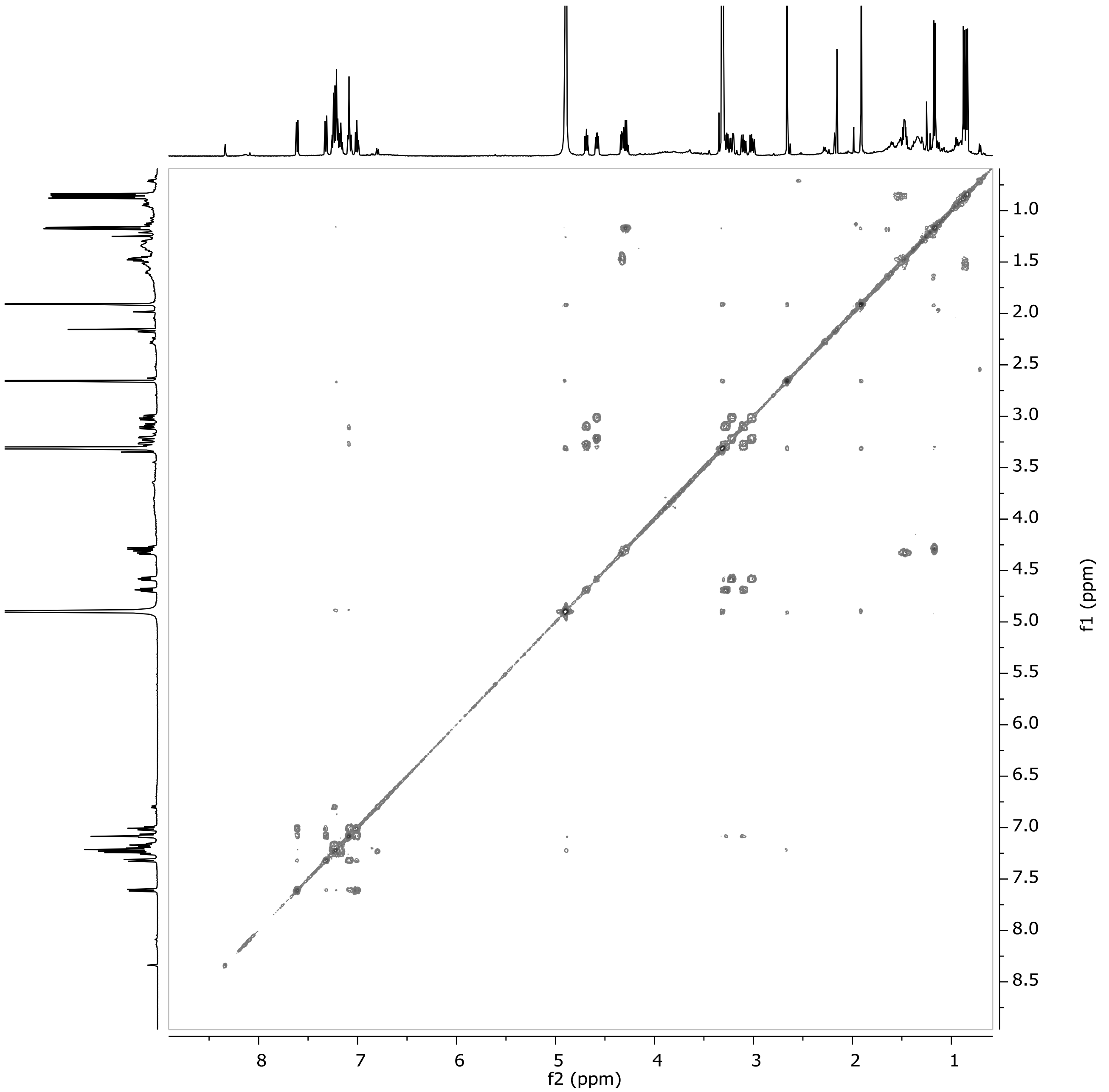

Figure S13. ^1H-^1H COSY spectrum of **2** in methanol-d_4 at 500 MHz.

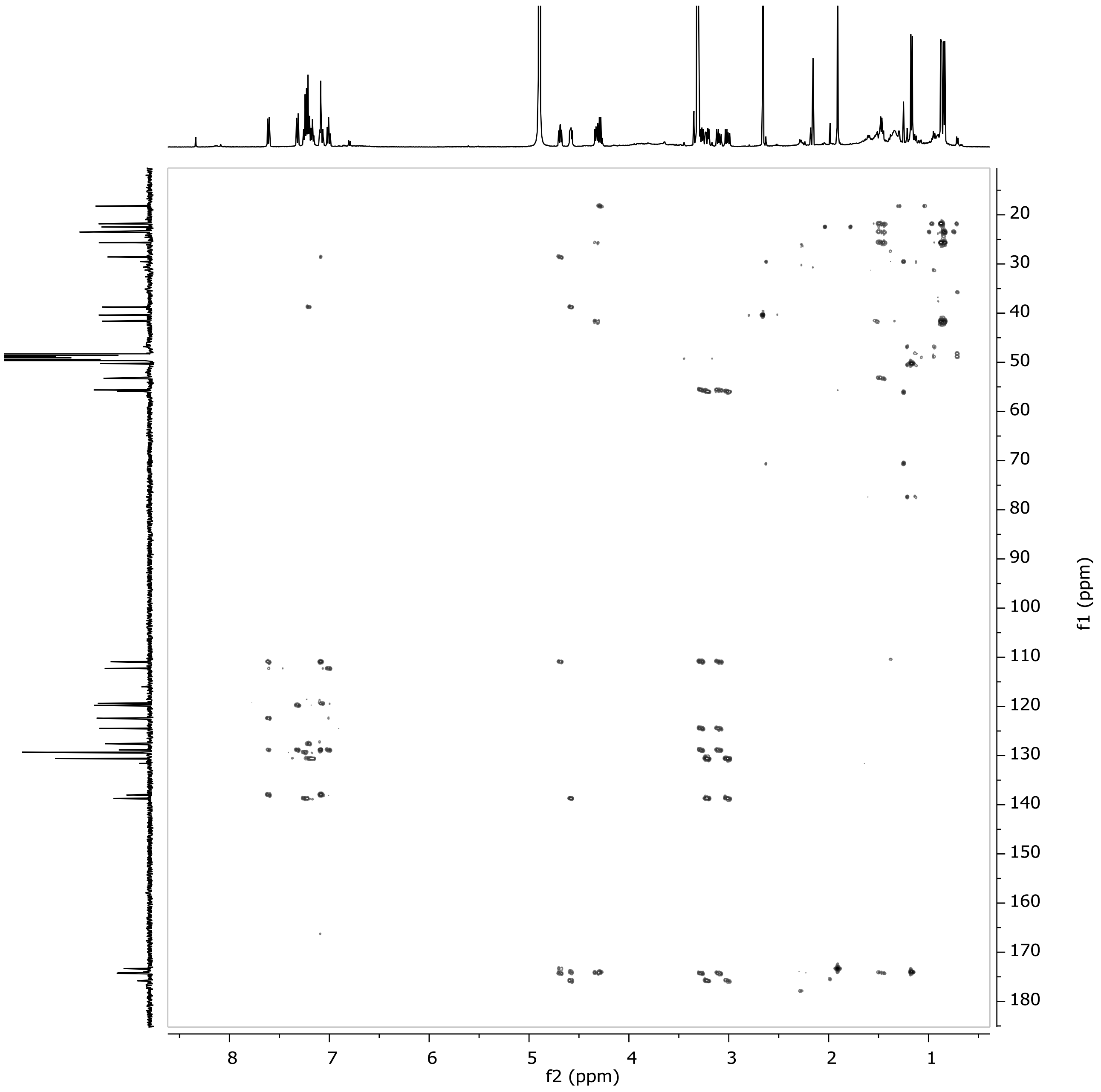

Figure S14. HMBC spectrum of **2** in methanol-d_4 at 500 MHz.

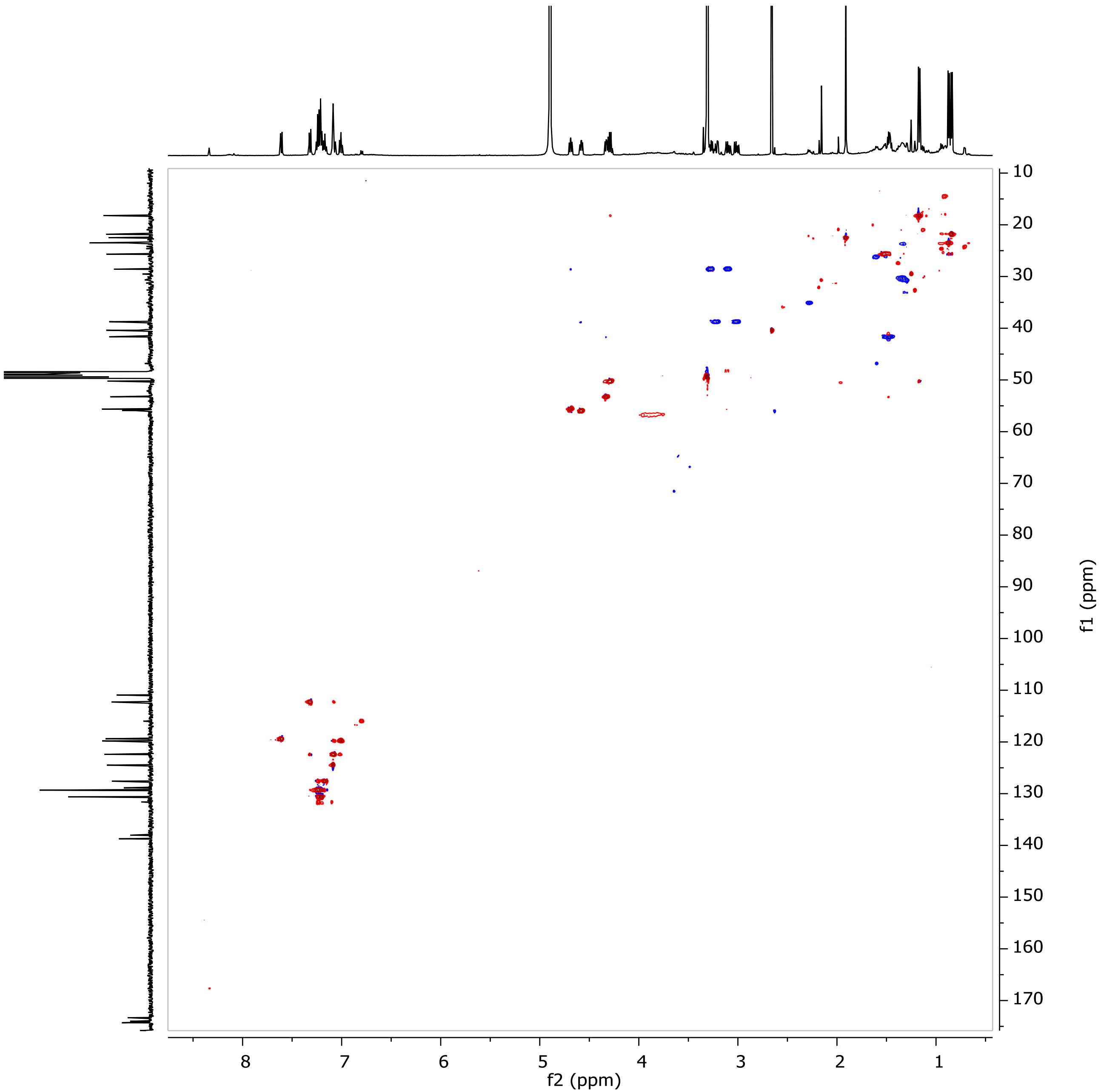

Figure S15. HSQC spectrum of **2** in methanol-d_4 at 500 MHz.

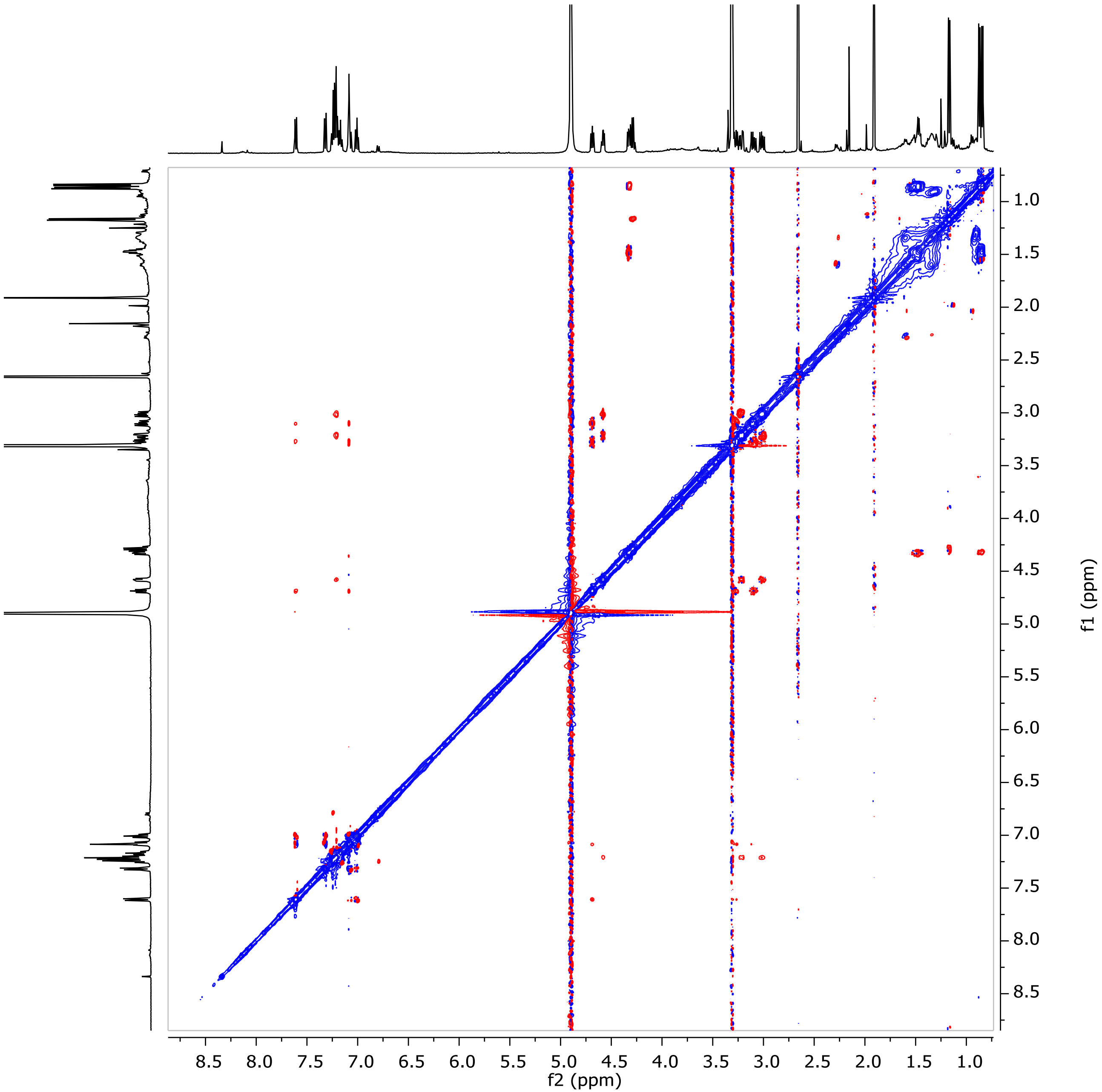

Figure S16. ROESY spectrum of **2** in methanol-d_4 at 500 MHz.

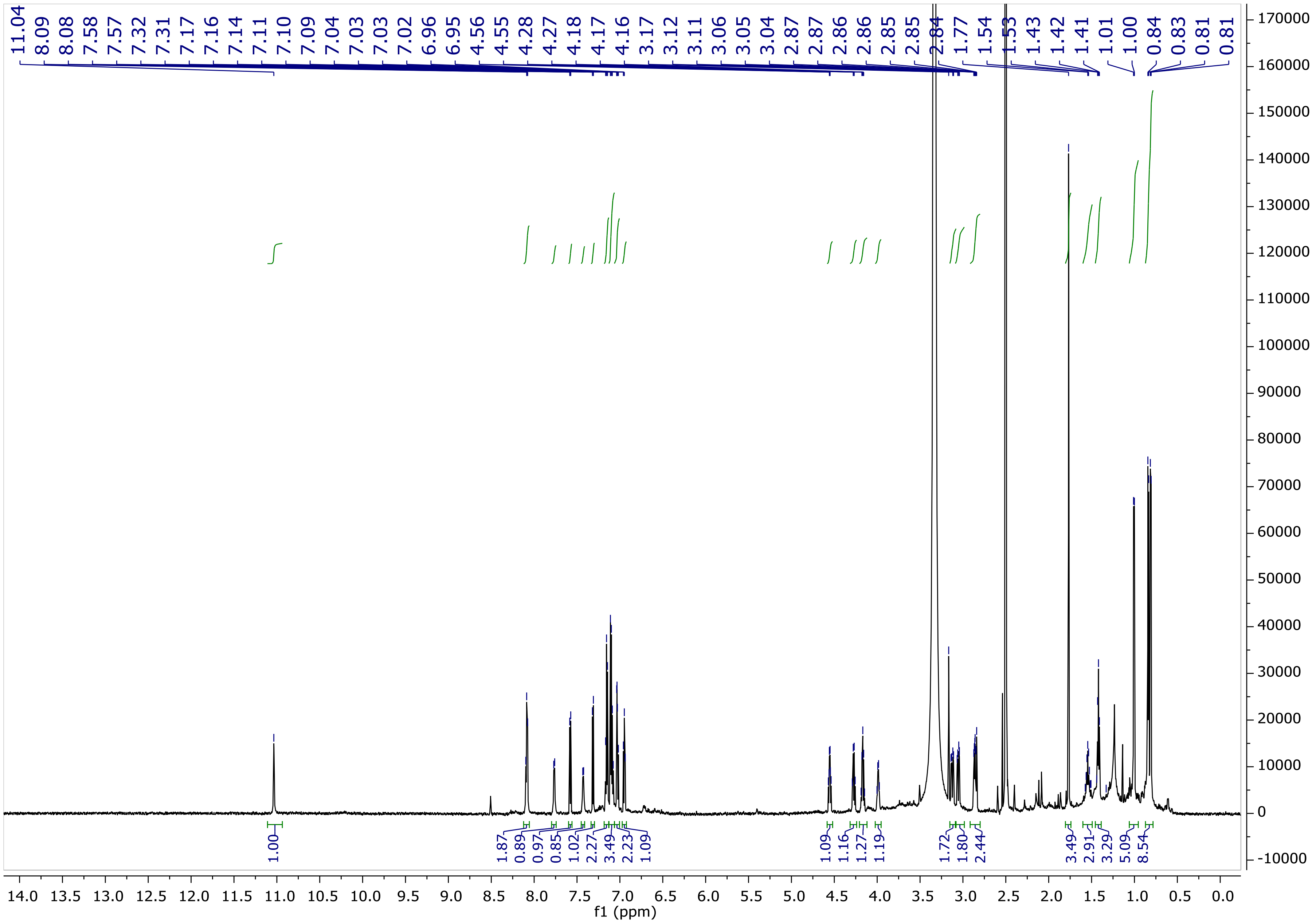

Figure S17. ^{1}H NMR spectrum of **2** in DMSO-d_6 at 700 MHz.

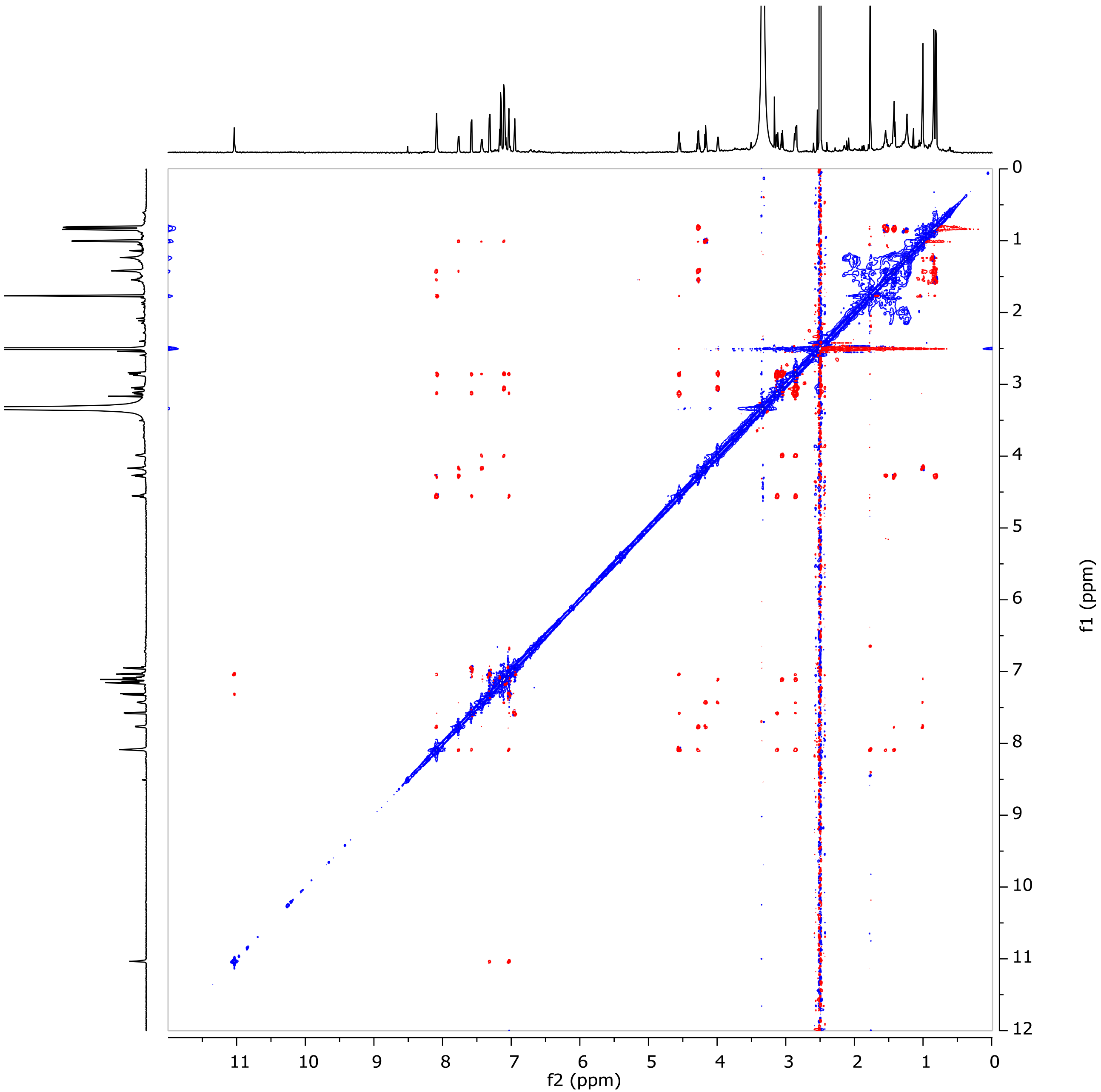

Figure S18. ROESY spectrum of **2** in DMSO-d_6 at 700 MHz.

Display Report

Analysis Info

Analysis Name	S:\PEOPLE\cho23_Caren Holzenkamp\NMR\MS-Data\purified fractions\MeOH-F9+F10-F14-577\MyNe_03_02_06_MeOH_F9+F10_F14_RD6_01_51539.d	Acquisition Date	10.10.2023 01:11:32
Method	51539.m	Operator	tti
Sample Name	MyNe_03_02_06_MeOH_F9+F10_F14	Instrument	amaZon speed 374444.06030
Comment			

Acquisition Parameter

Ion Polarity Negative

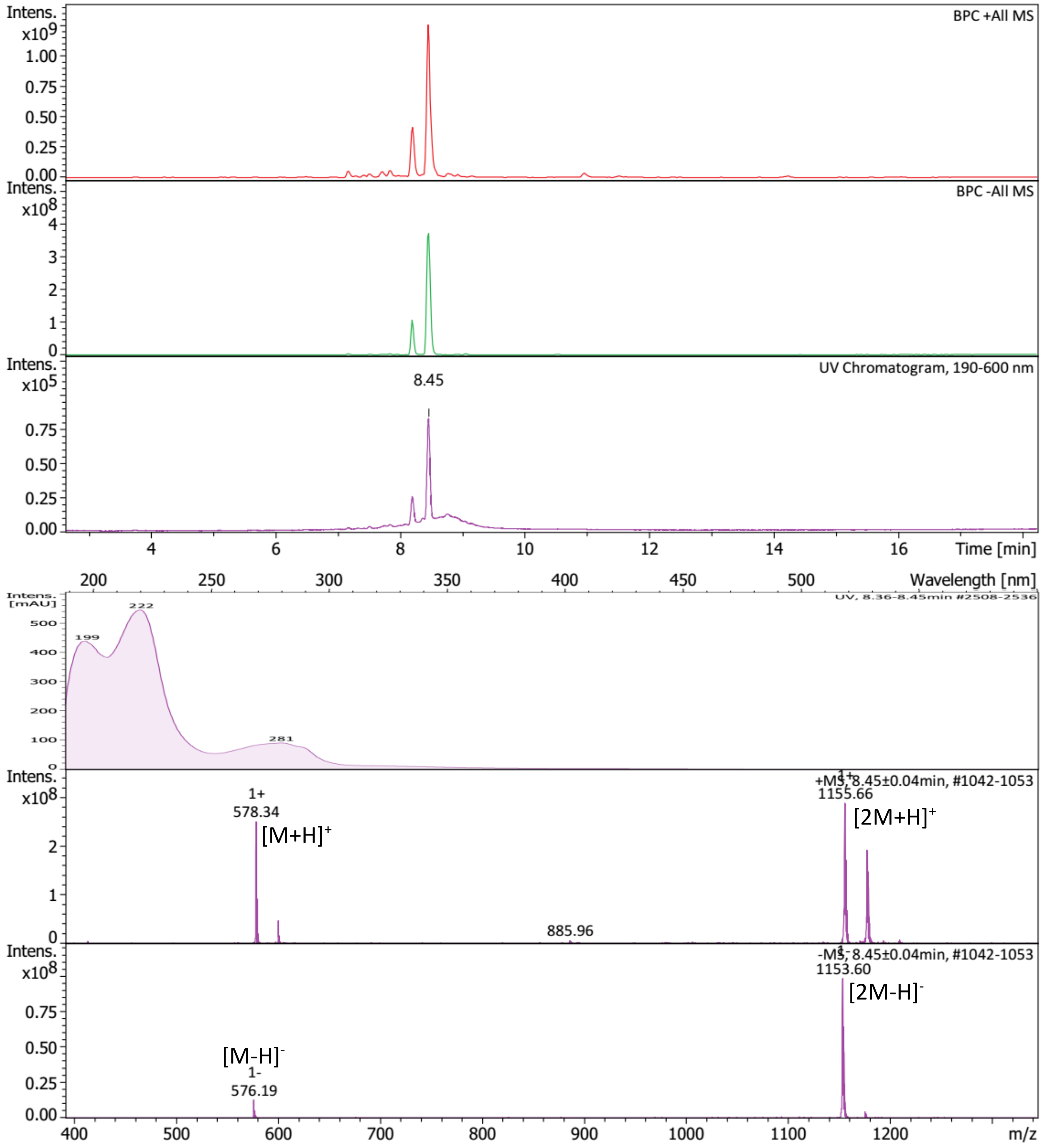

Bruker Compass DataAnalysis 6.1 printed: 06.11.2023 11:11:43 by: sel22 Page 1 of 1

Figure S19. LR-ESI-MS of **3**.

Display Report

Analysis Info

Analysis Name	S:\PEOPLE\cho23_Caren Holzenkamp\NMR\MS-Data\purified fractions\MeOH-F9+F10-F14-577\MyNe-03-02-06-MeOH-F9+F10-F14_P1-A-7_1_898.d	Acquisition Date	03.11.2023 01:14:05
Method	MWIS_BEH50mm_25min_ohne tims.m	Operator	Demo User
Sample Name	MyNe-03-02-06-MeOH-F9+F10-F14	Instrument	timsTOF Pro 2 1875087.10646
Comment			

Acquisition Parameter

Source Type	ESI	Ion Polarity	Positive	Set Nebulizer	1.0 Bar
Focus	Active	Set Capillary	4000 V	Set Dry Heater	200 °C
Scan Begin	150 m/z	Set End Plate Offset	-500 V	Set Dry Gas	5.0 l/min
Scan End	2500 m/z	Set Collision Cell RF	1000.0 Vpp	Set Divert Valve	Waste

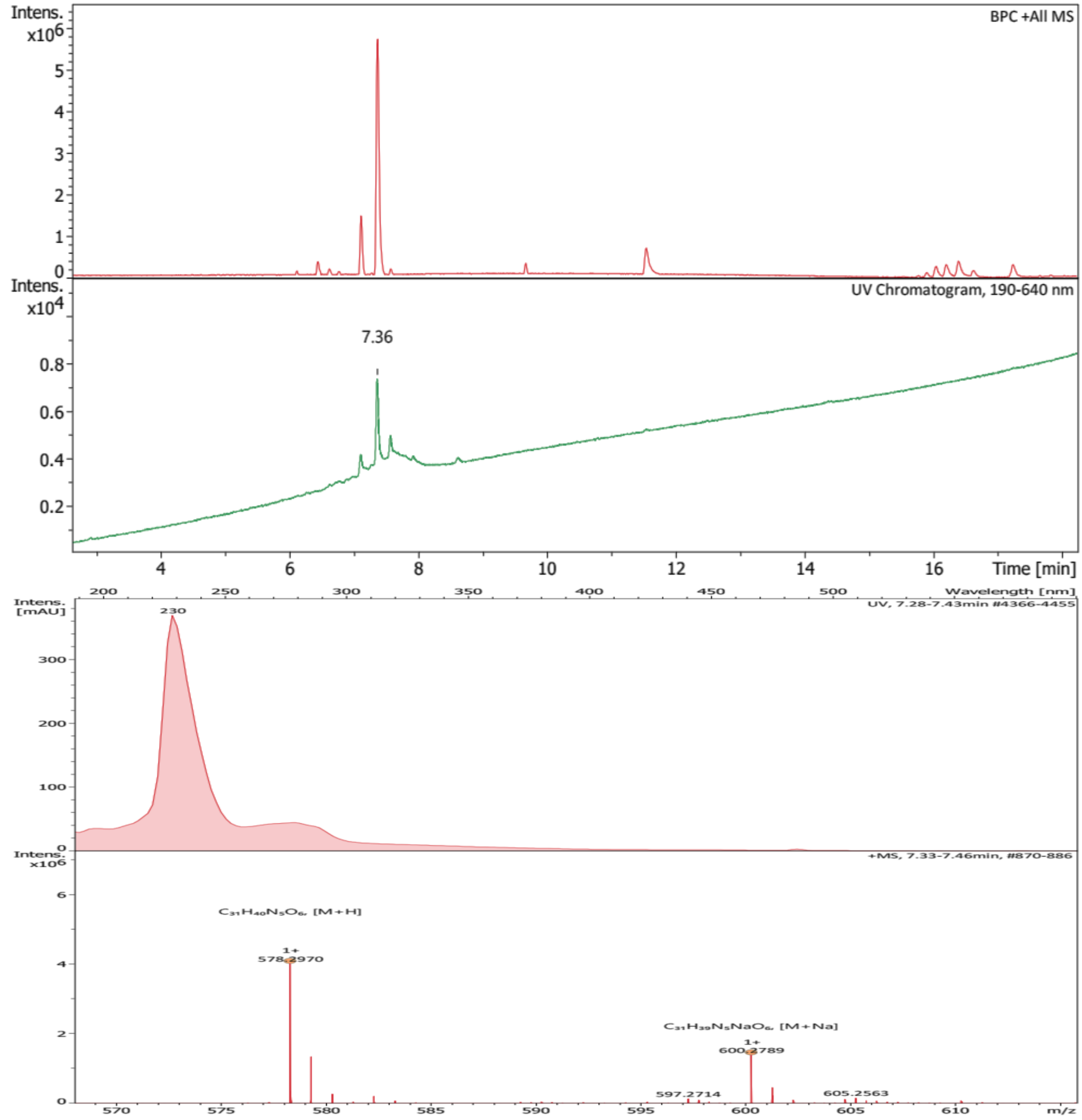

Bruker Compass DataAnalysis 6.1 printed: 06.11.2023 11:18:16 by: sel22 Page 1 of 1

Figure S20. HR-ESI-MS of **3**.

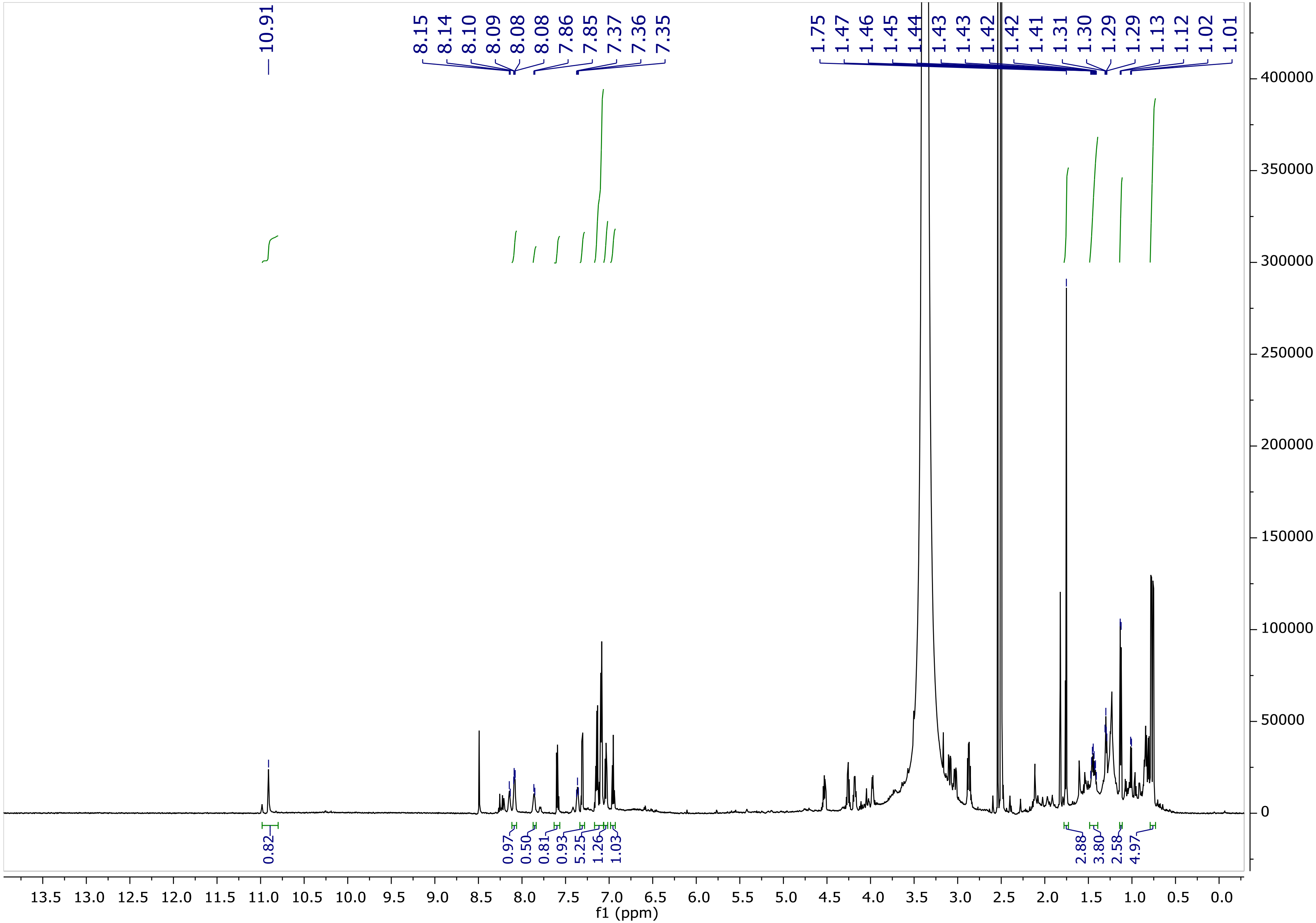

Figure S21. ^{1}H NMR spectrum of **3** in DMSO-d_6 at 700 MHz.

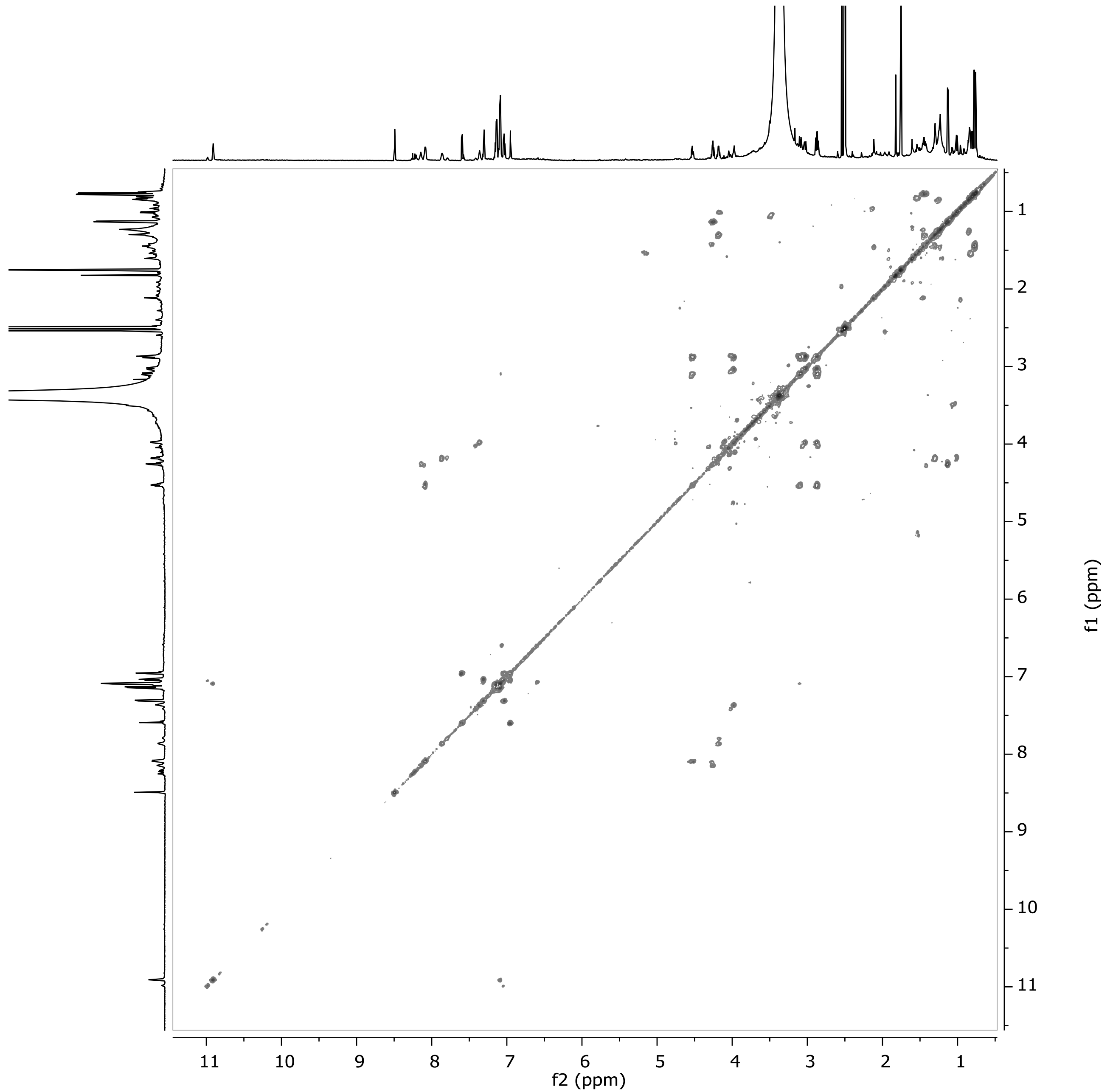

Figure S22. ^{1}H-^{1}H COSY spectrum of **3** in DMSO-d_6 at 700 MHz.

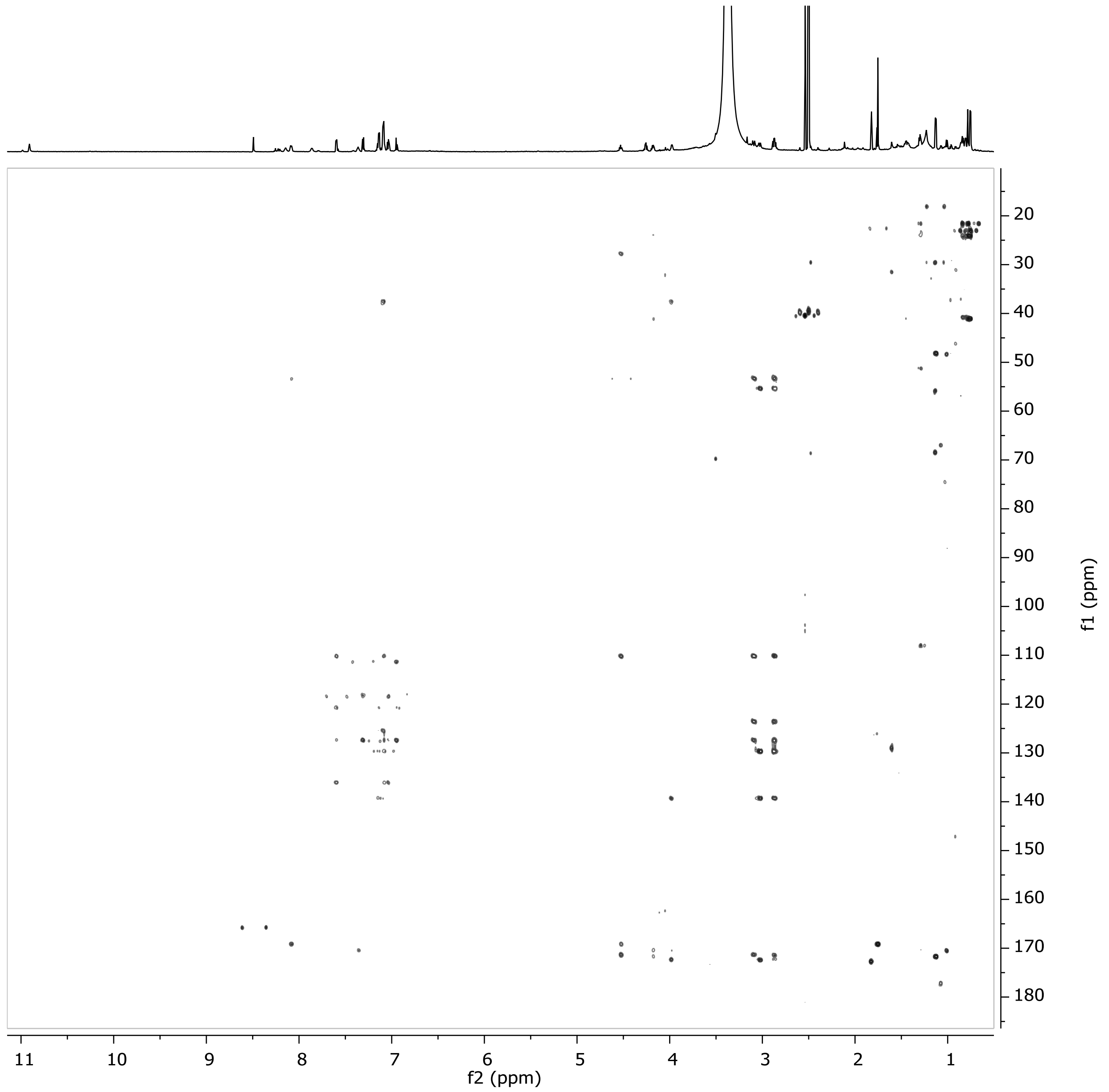

Figure S23. HMBC spectrum of **3** in DMSO-d_6 at 700 MHz.

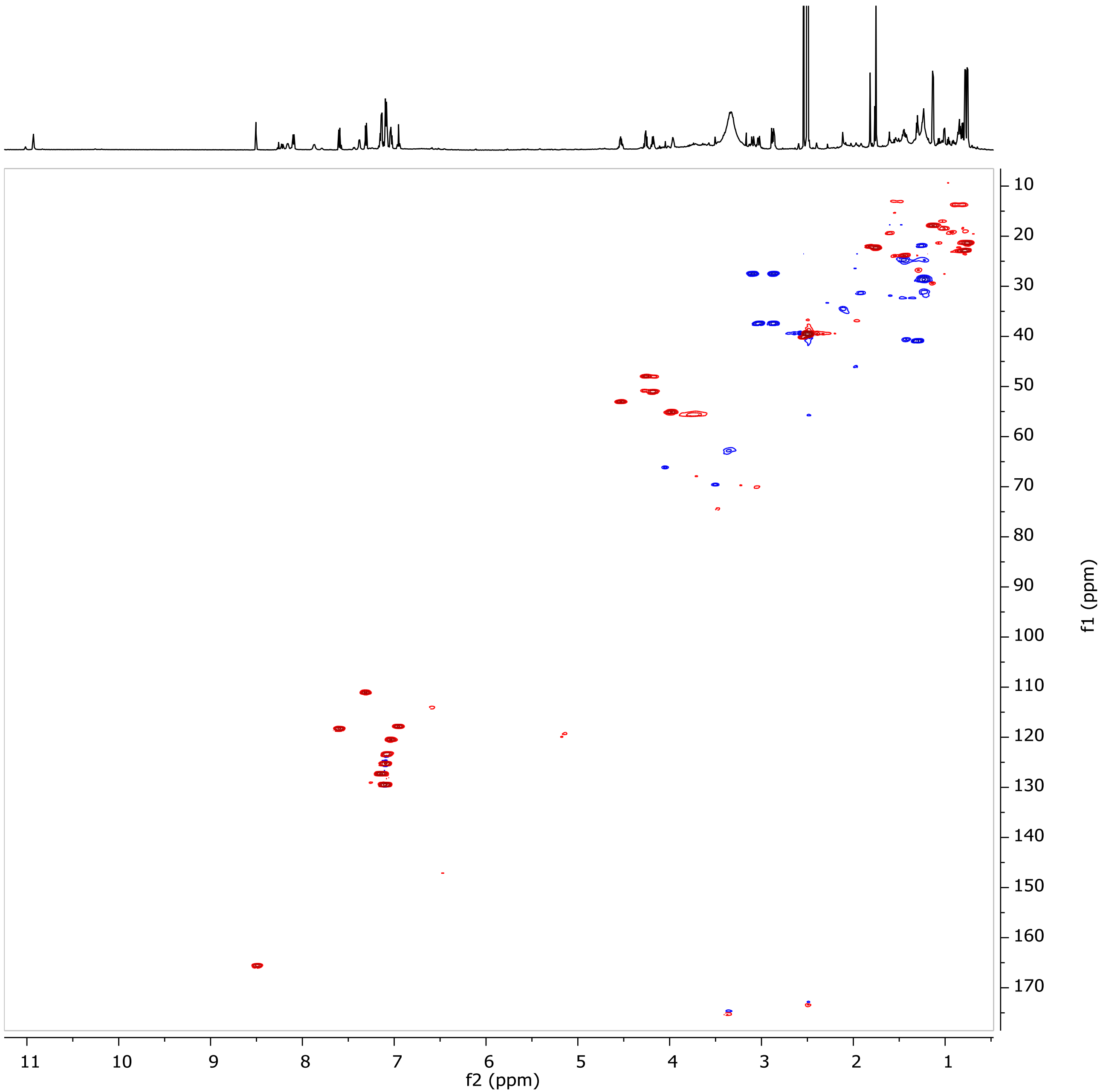

Figure S24. HSQC spectrum of **3** in DMSO-d_6 at 700 MHz.

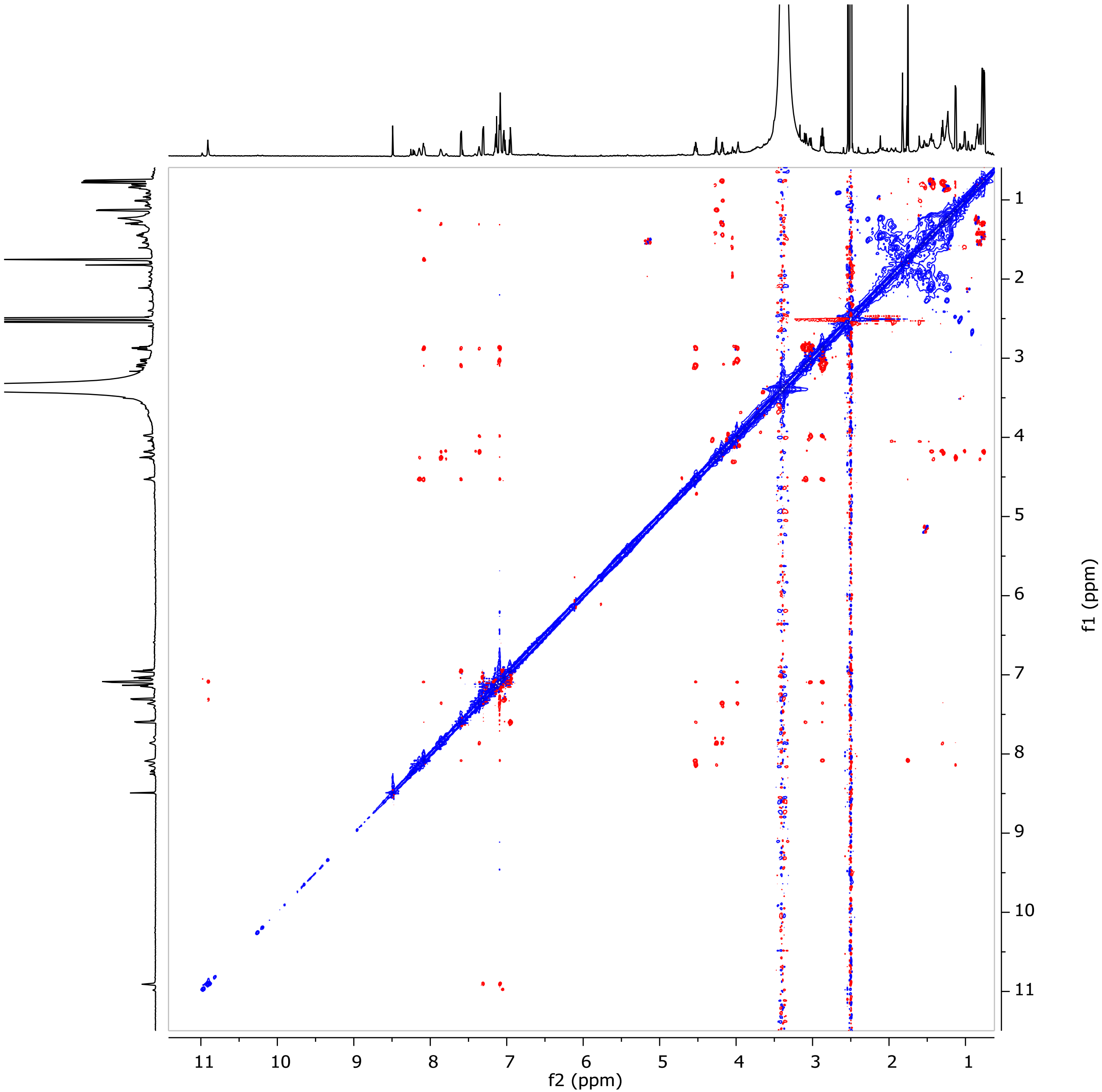

Figure S25. ROESY spectrum of **3** in DMSO-d_6 at 700 MHz.

Generic Display Report (all)

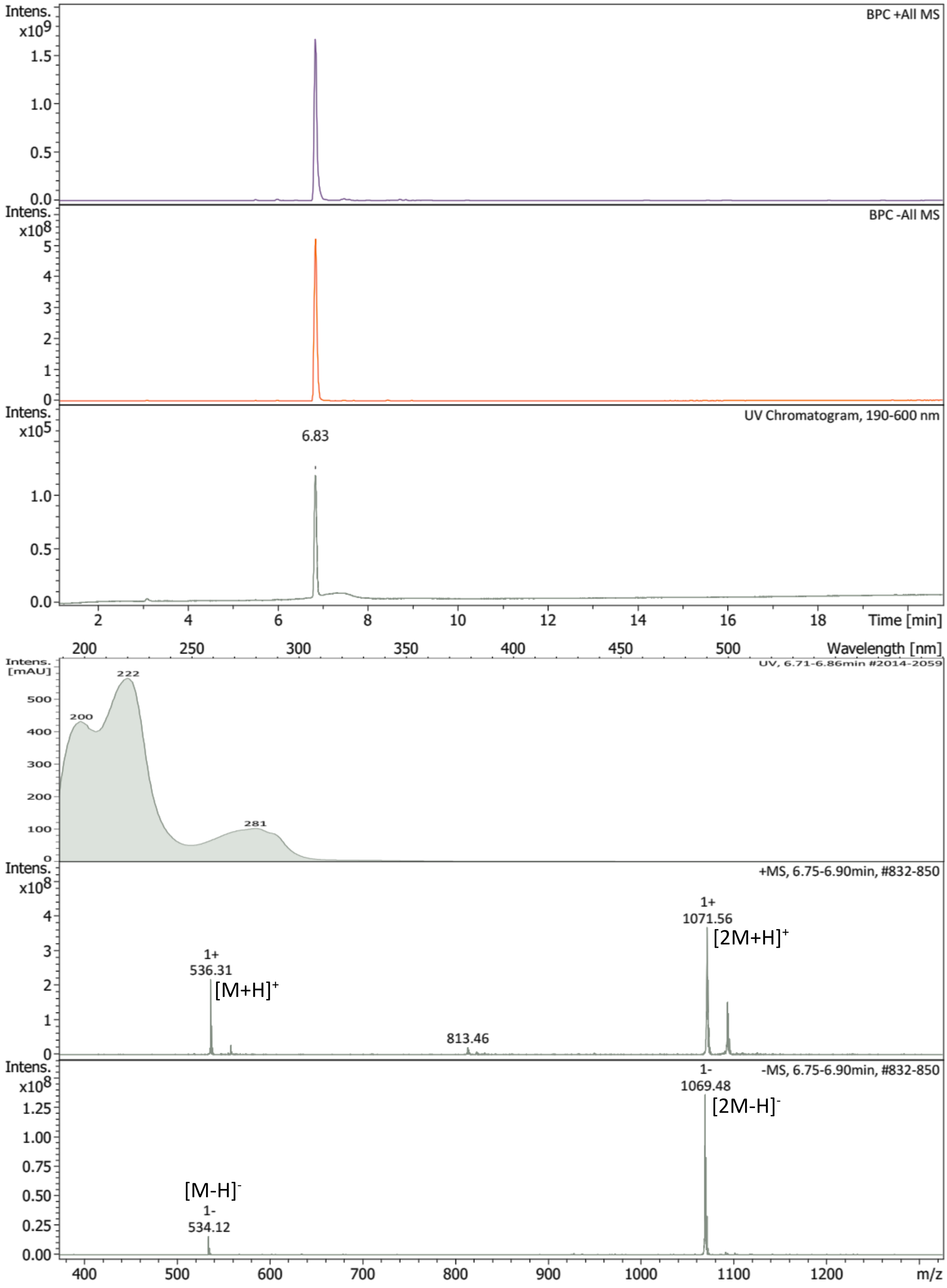

Bruker Compass DataAnalysis 6.1 printed: 06.11.2023 11:02:34 by: sel22 Page 1 of 1

Figure S26. LR-ESI-MS of **4**.

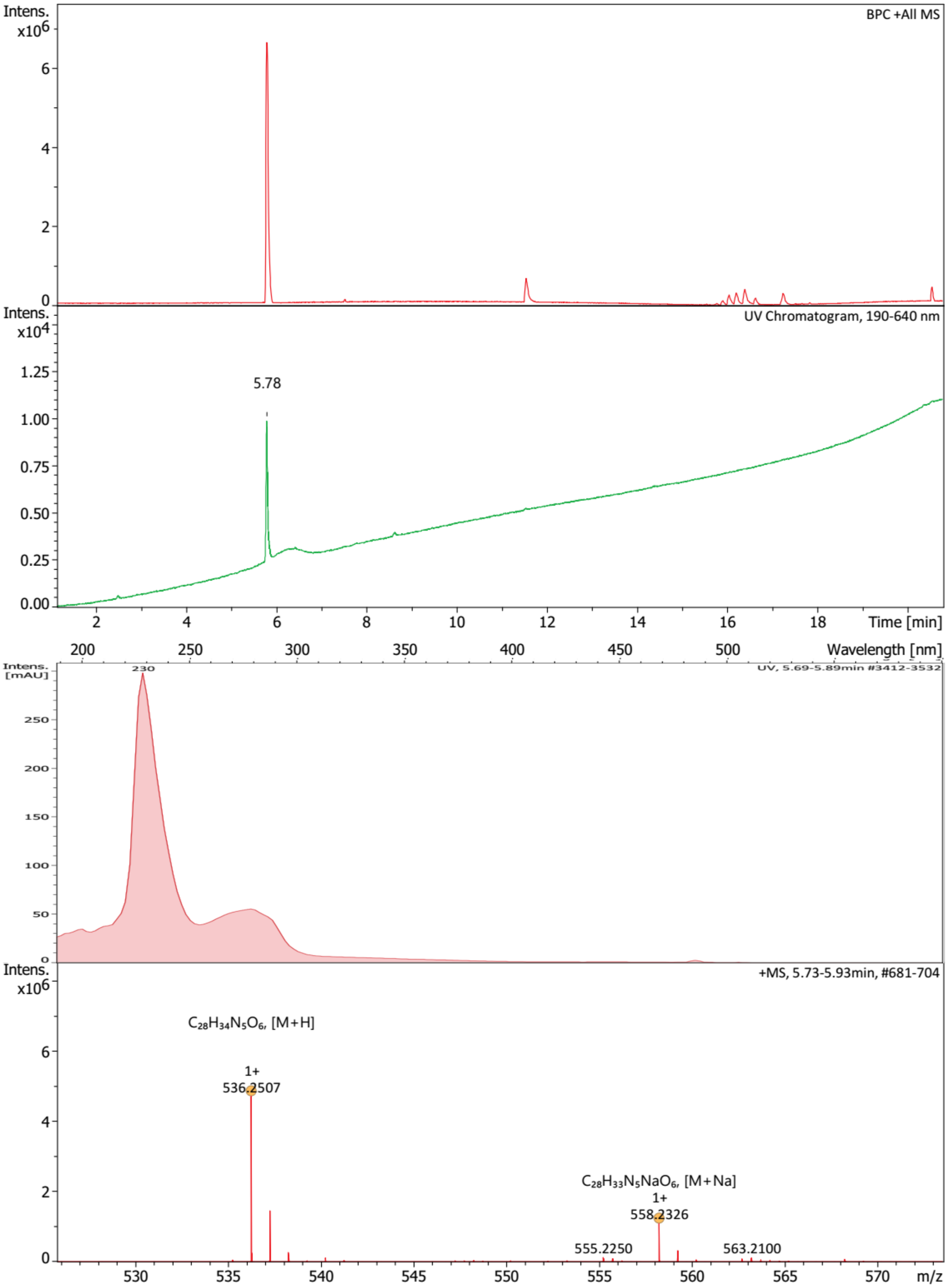

Figure S27. HR-ESI-MS of **4**.

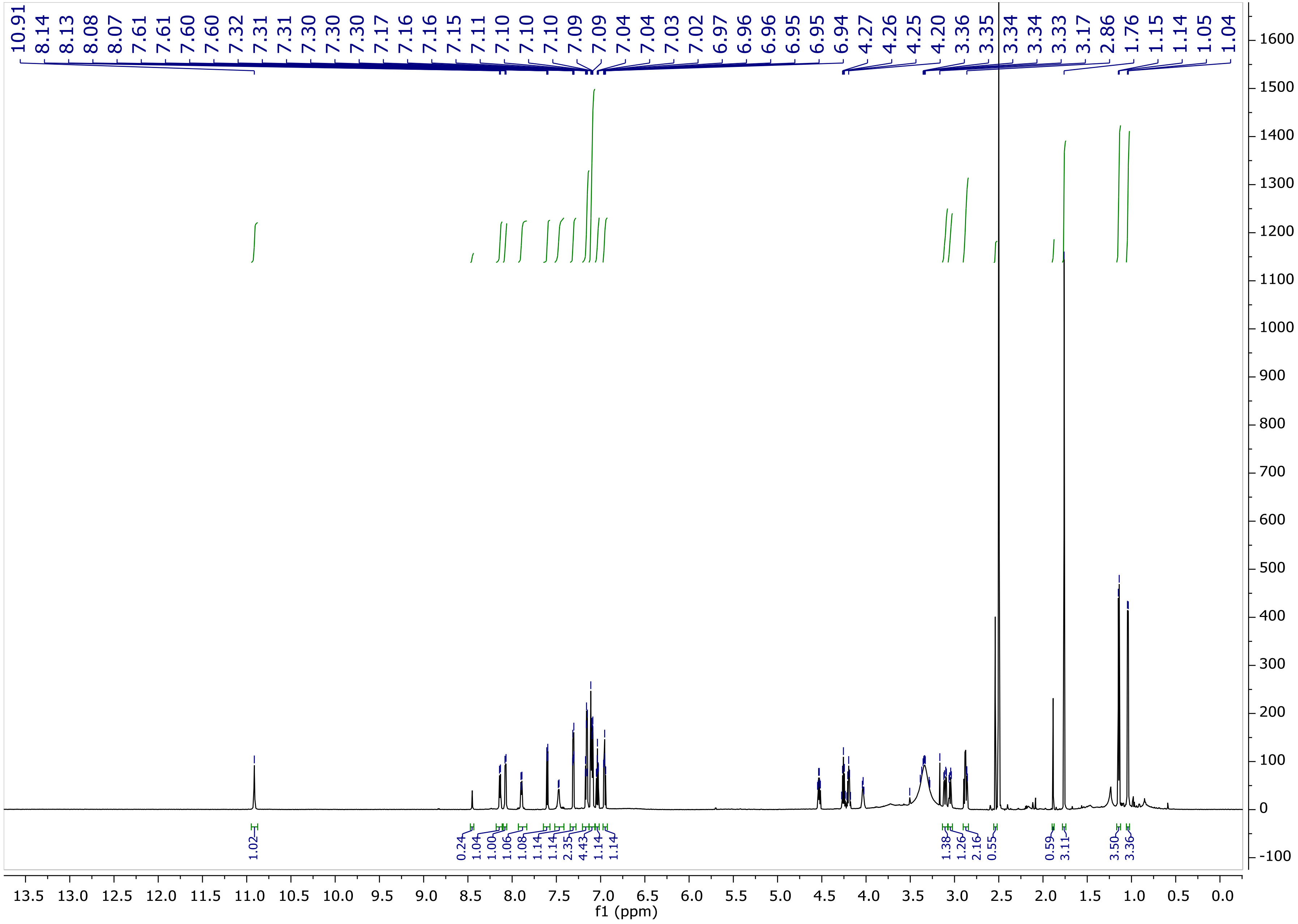

Figure S28. ^{1}H NMR spectrum of **4** in DMSO-d_6 at 700 MHz.

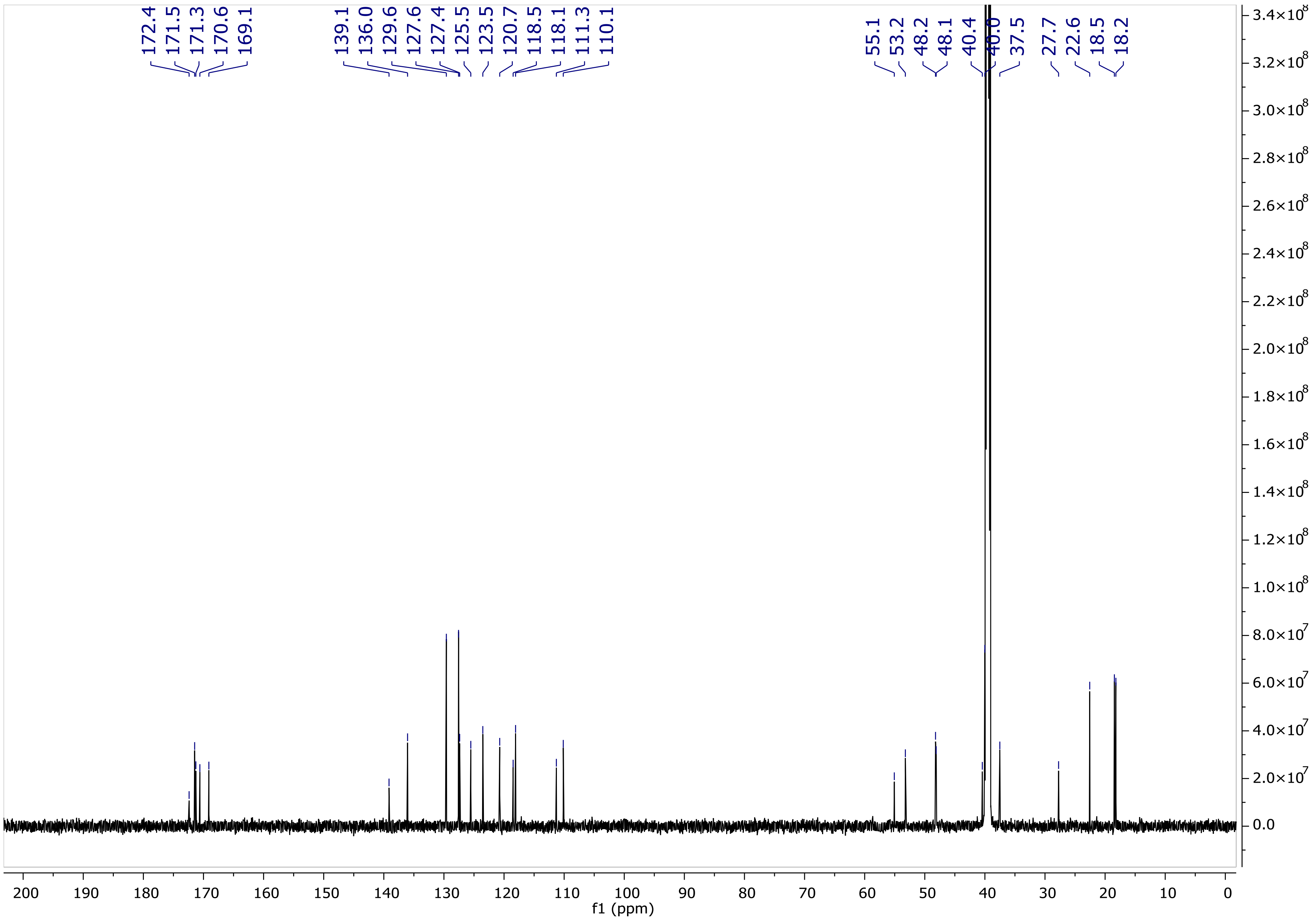

Figure S29. ^{13}C NMR spectrum of **4** in DMSO-d_6 at 175 MHz.

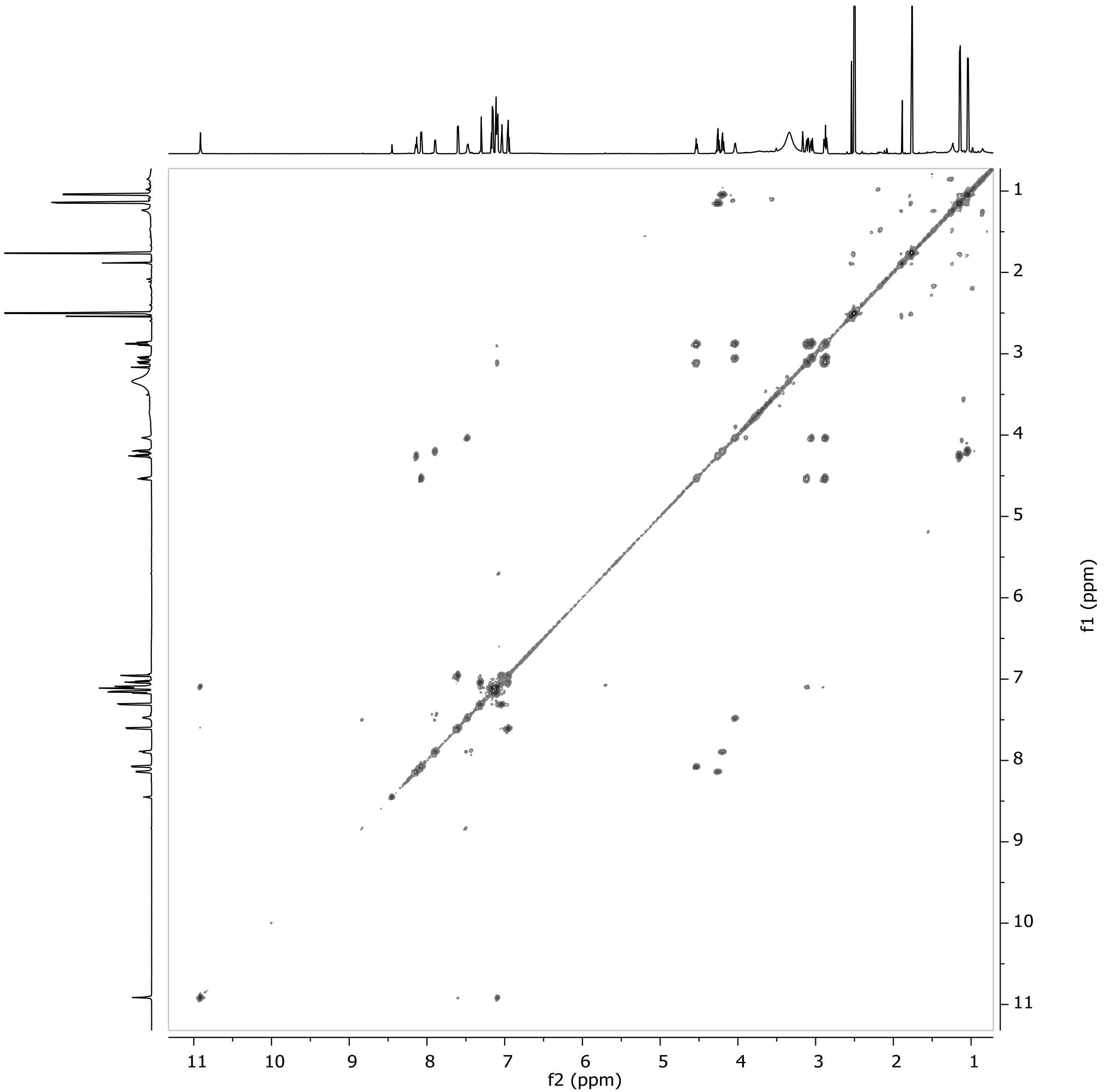

Figure S30. ^1H-^1H COSY spectrum of **4** in DMSO-d_6 at 700 MHz.

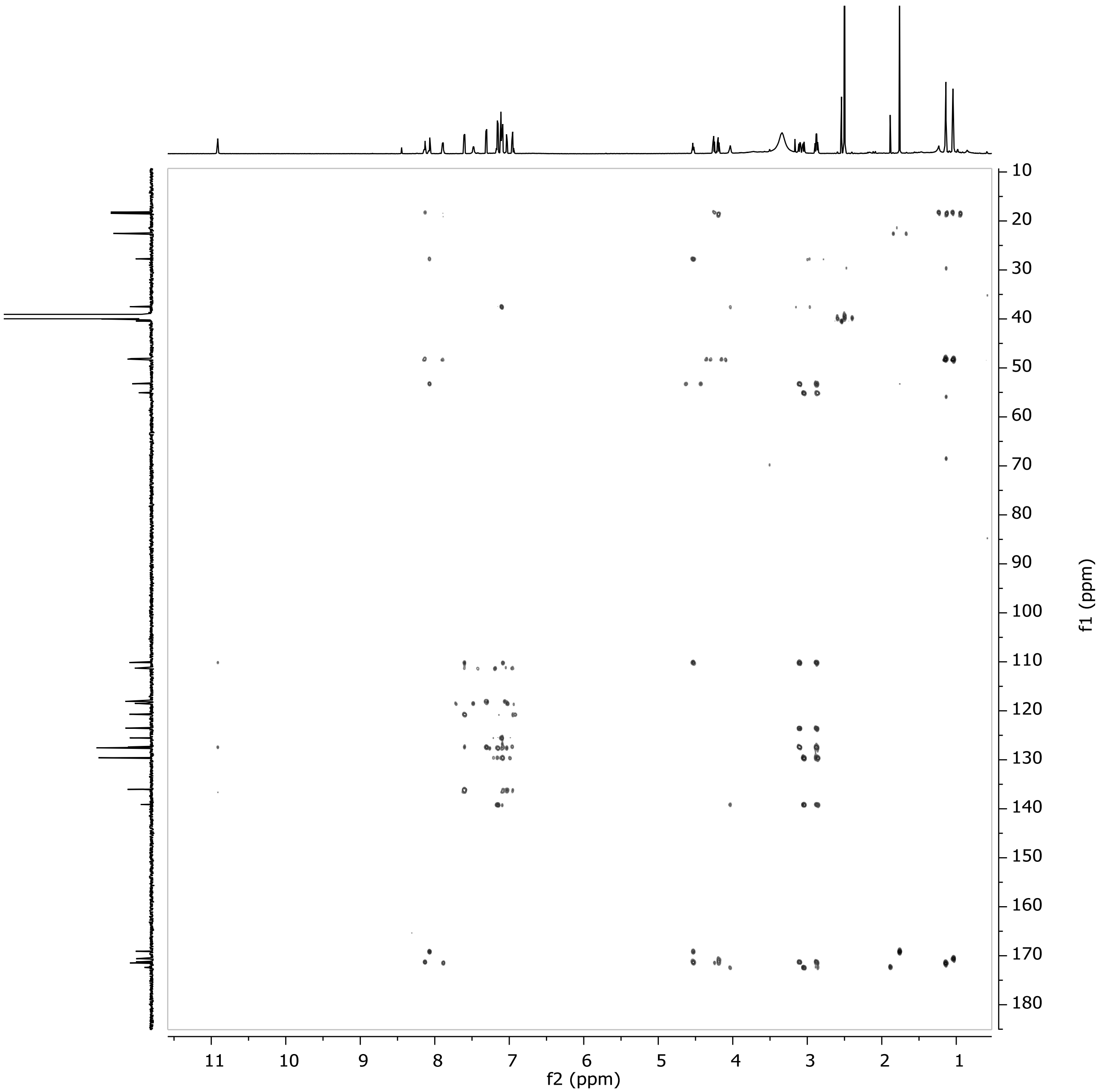

Figure S31. HMBC spectrum of **4** in DMSO-d_6 at 700 MHz.

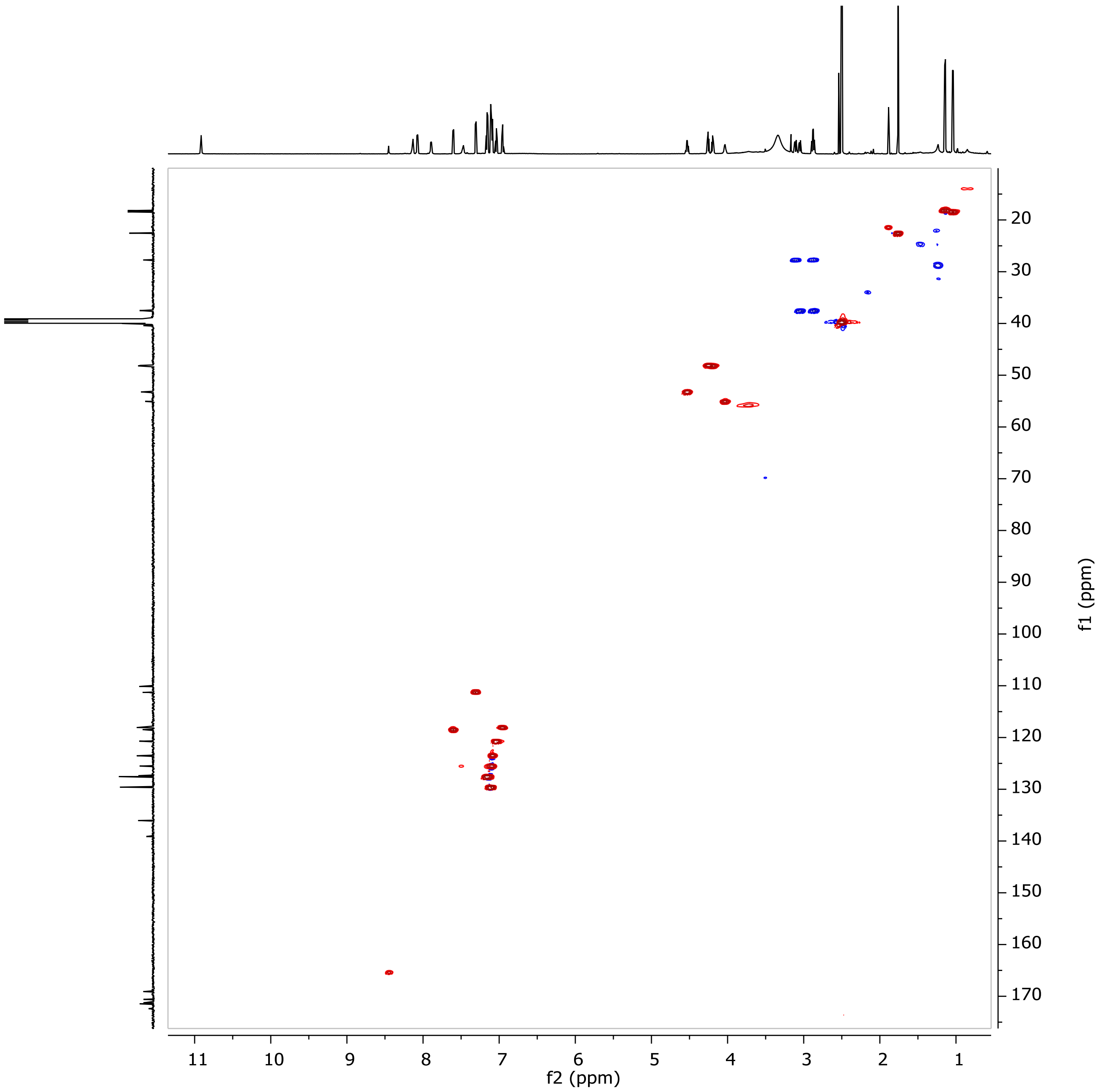

Figure S32. HSQC spectrum of **4** in DMSO-d_6 at 700 MHz.

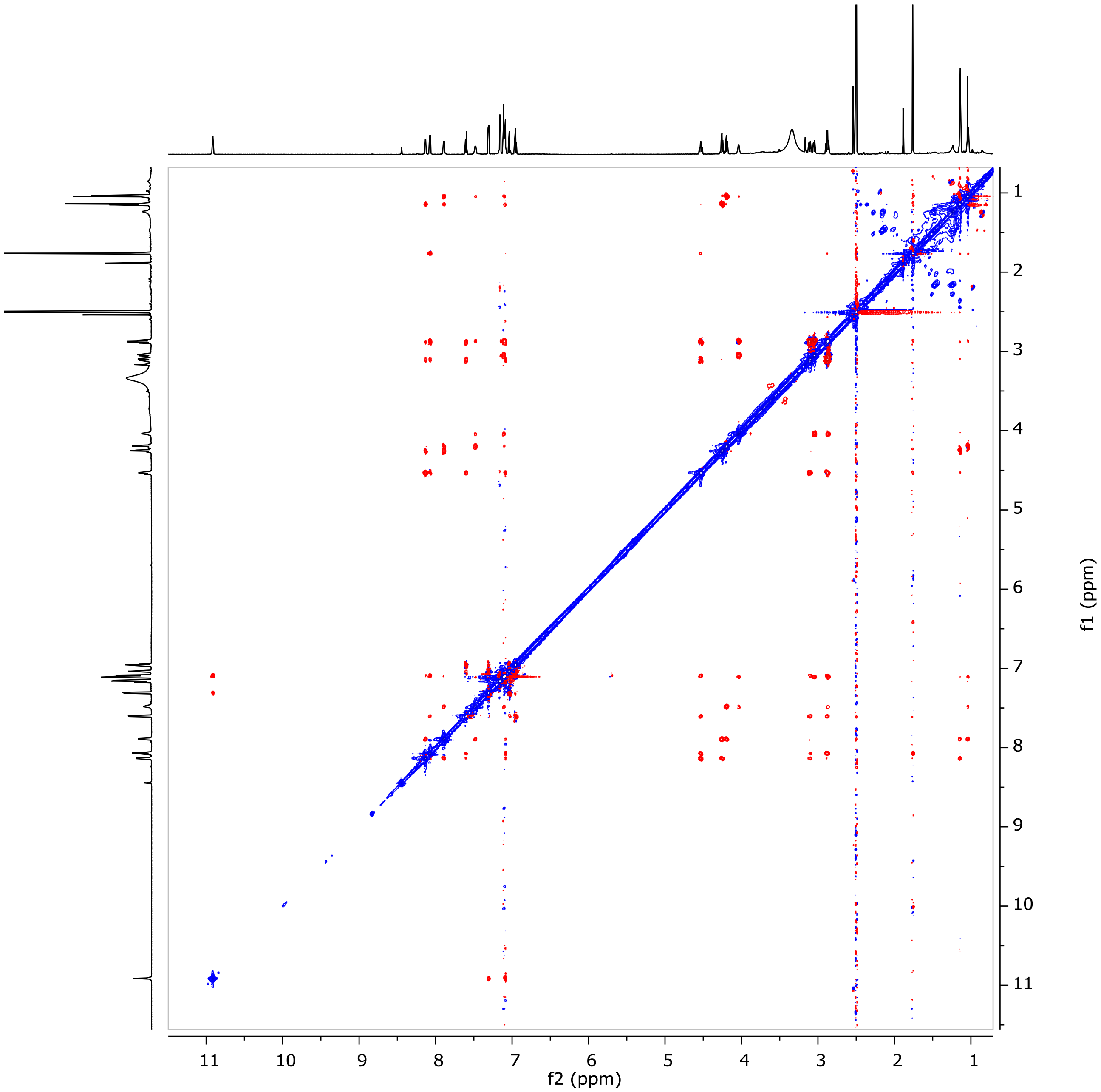

Figure S33. ROESY spectrum of **4** in DMSO-d_6 at 700 MHz.

Display Report

Analysis Info

Analysis Name	S:\PEOPLE\cho23_Caren Holzenkamp\NMR\MS-Data\purified fractions\MeOH-F9+F10-F6-430\MyNe_03_02_06_MeOH_F9+F10_F6_RC6_01_51525.d	Acquisition Date	09.10.2023 16:44:01
Method	51525.m	Operator	tti
Sample Name	MyNe_03_02_06_MeOH_F9+F10_F6	Instrument	amaZon speed 374444.06030
Comment			

Acquisition Parameter

Ion Polarity Positive

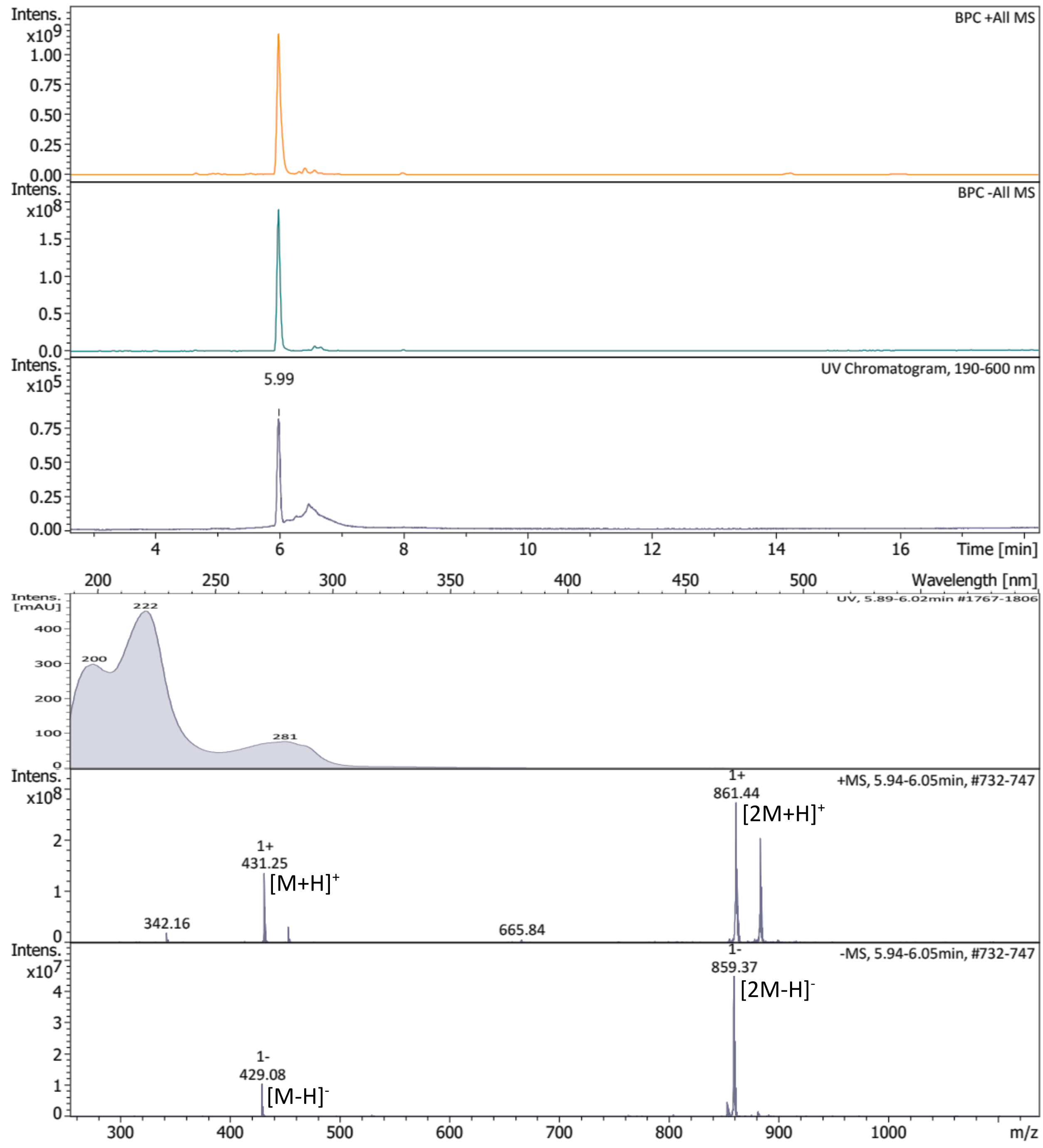

Bruker Compass DataAnalysis 6.1 printed: 06.11.2023 11:37:37 by: sel22 Page 1 of 1

Figure S34. LR-ESI-MS of **5**.

Display Report

**Analysis Info**		Acquisition Date	02.11.2023 23:41:16
Analysis Name	S:\PEOPLE\cho23_Caren Holzenkamp\NMR\MS-Data\purified fractions\MeOH-F9+F10-F6-430\MyNe-03-02-06-MeOH-F9+F10-F6_P1-A-4_1_895.d		
Method	MWIS_BEH50mm_25min_ohnetims.m	Operator	Demo User
Sample Name	MyNe-03-02-06-MeOH-F9+F10-F6	Instrument	timsTOF Pro 2 1875087.10646
Comment			

Acquisition Parameter

Source Type	ESI	Ion Polarity	Positive	Set Nebulizer	1.0 Bar
Focus	Active	Set Capillary	4000 V	Set Dry Heater	200 °C
Scan Begin	150 m/z	Set End Plate Offset	-500 V	Set Dry Gas	5.0 l/min
Scan End	2500 m/z	Set Collision Cell RF	1000.0 Vpp	Set Divert Valve	Waste

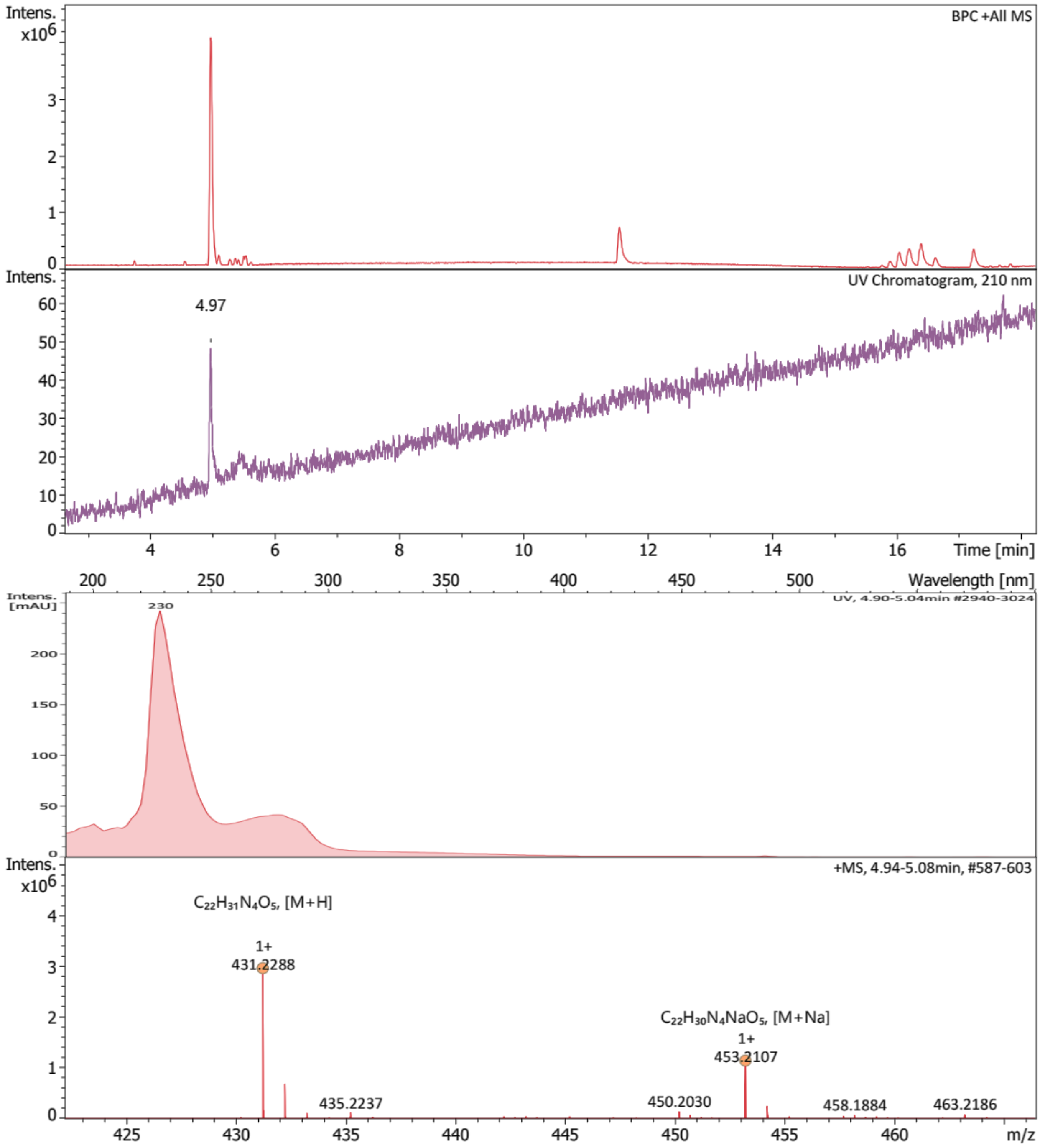

Bruker Compass DataAnalysis 6.1 printed: 06.11.2023 11:39:34 by: sel22 Page 1 of 1

Figure S35. HR-ESI-MS of **5**.

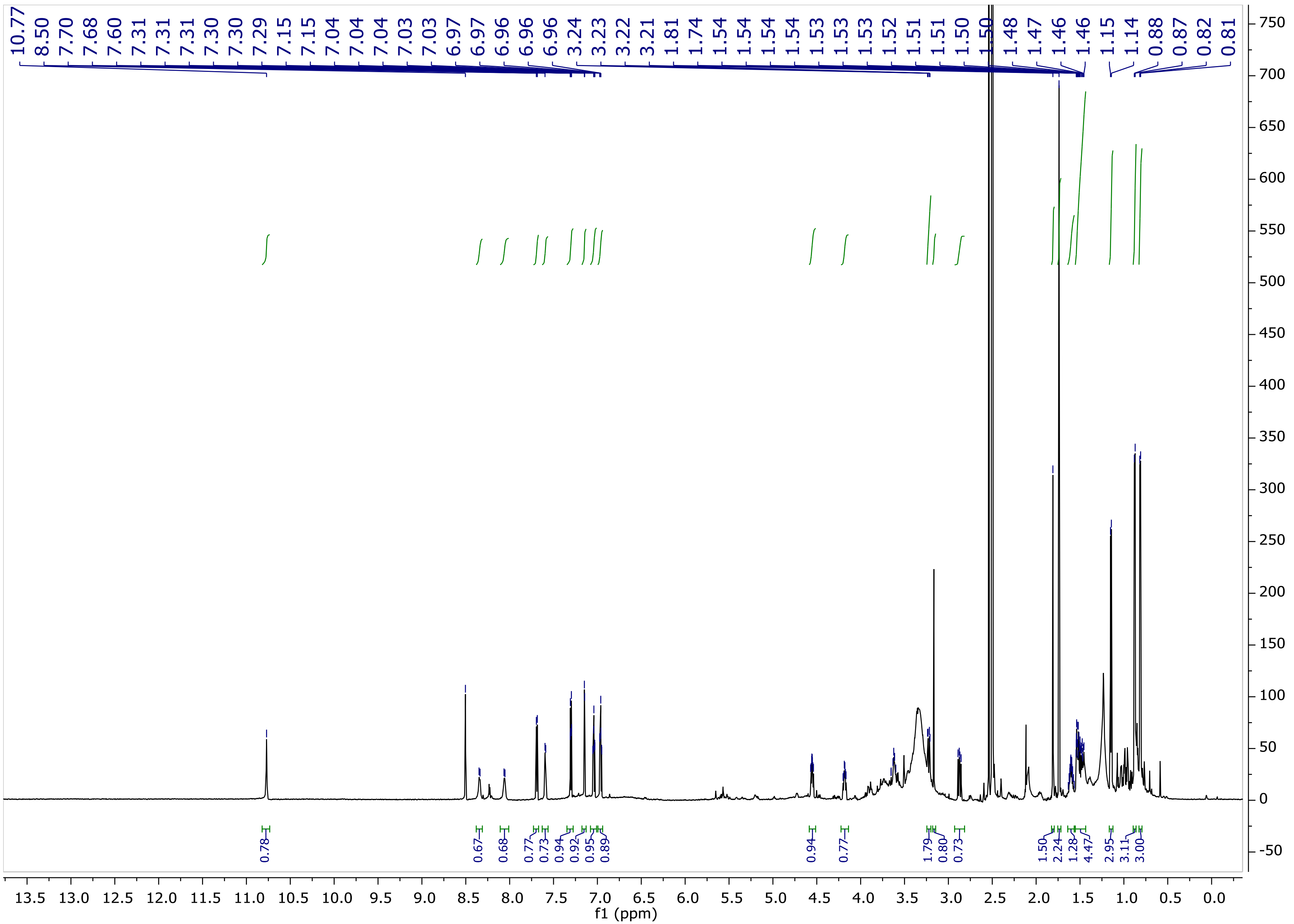

Figure S36. ^{1}H NMR spectrum of **5** in DMSO-d_6 at 700 MHz.

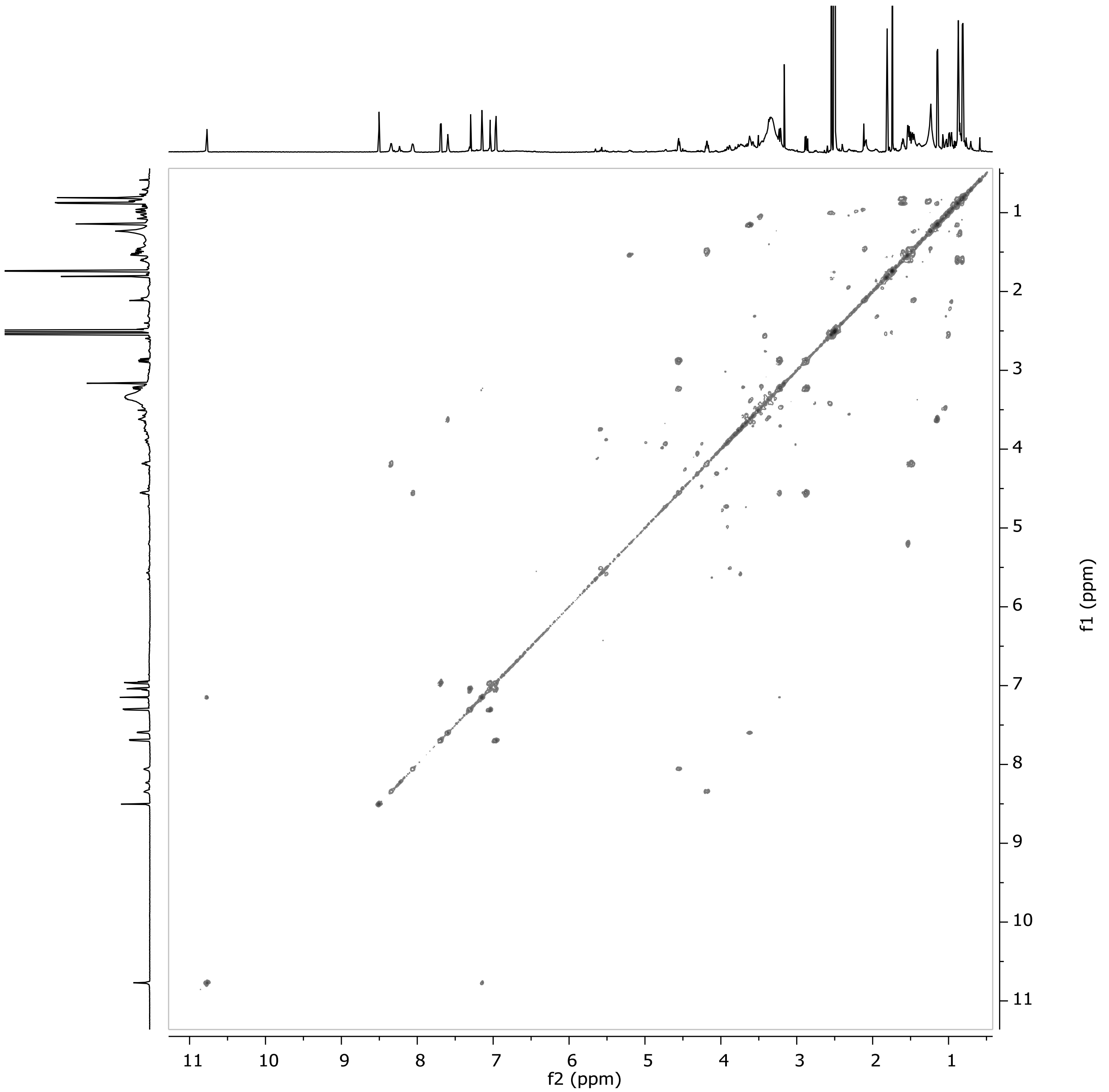

Figure S37. ^{1}H-^{1}H COSY spectrum of **5** in DMSO-d_6 at 700 MHz.

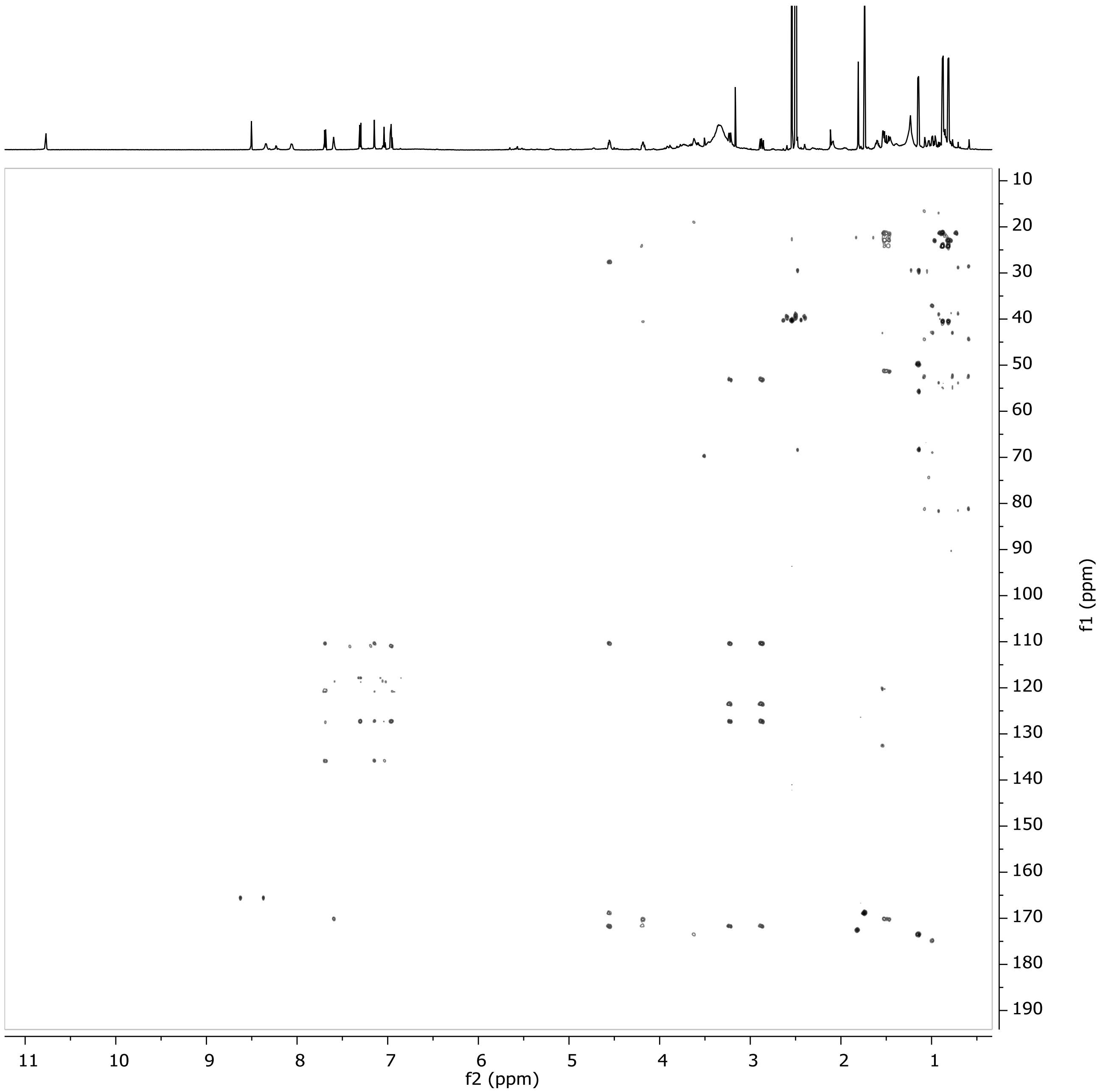

Figure S38. HMBC spectrum of **5** in DMSO-d_6 at 700 MHz.

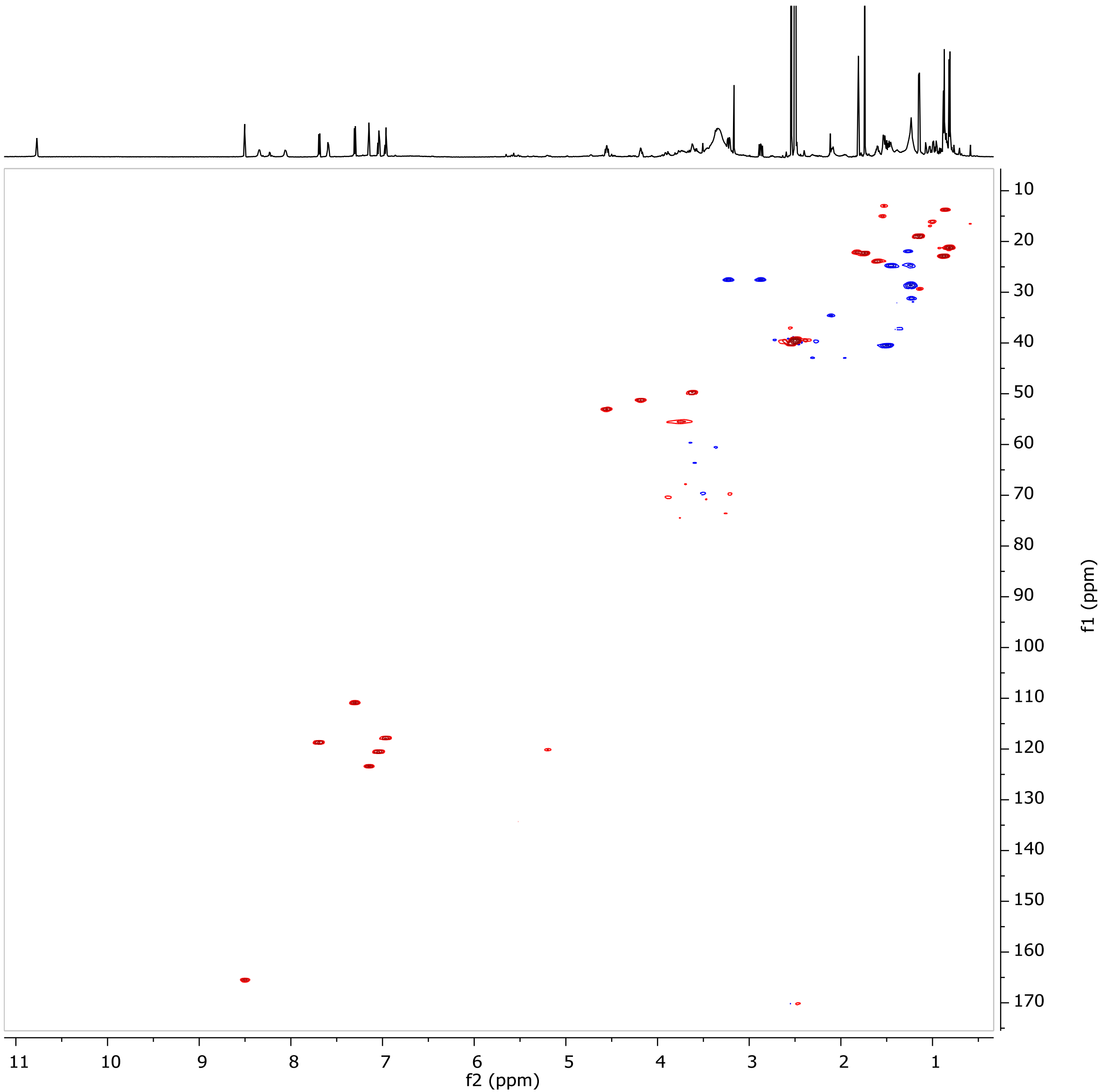

Figure S39. HSQC spectrum of **5** in DMSO-d_6 at 700 MHz.

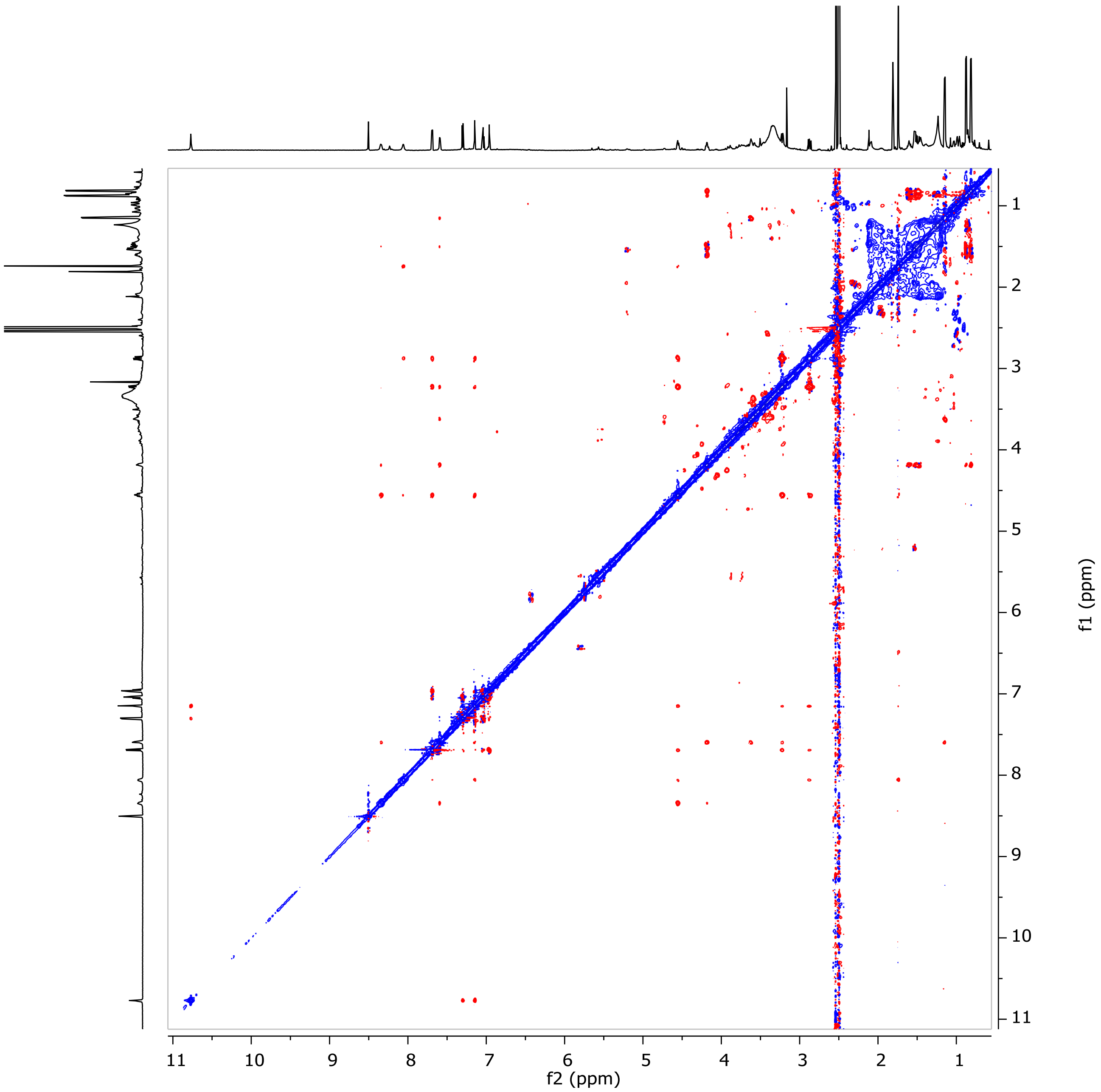

Figure S40. ROESY spectrum of **5** in DMSO-d_6 at 700 MHz.

Display Report

Analysis Info

Analysis Name	S:\PEOPLE\cho23_Caren Holzenkamp\NMR\MS-Data\purified fractions\MeOH-F11-IIF7-397\MyNe-03-02-06-MeOH-F11-II-F7_RE3_01_51707.d	Acquisition Date	14.10.2023 10:28:57
Method	51707.m	Operator	tti
Sample Name	MyNe-03-02-06-MeOH-F11-II-F7	Instrument	amaZon speed
Comment			

Acquisition Parameter

Ion Source Type	ESI	Ion Polarity	Negative	Alternating Ion Polarity	on
Mass Range Mode	UltraScan	Scan Begin	100 m/z	Scan End	2000 m/z
Accumulation Time	3702 µs	RF Level	100 %	Trap Drive	78.0
SPS Target Mass	1000 m/z	Averages	6 Spectra		

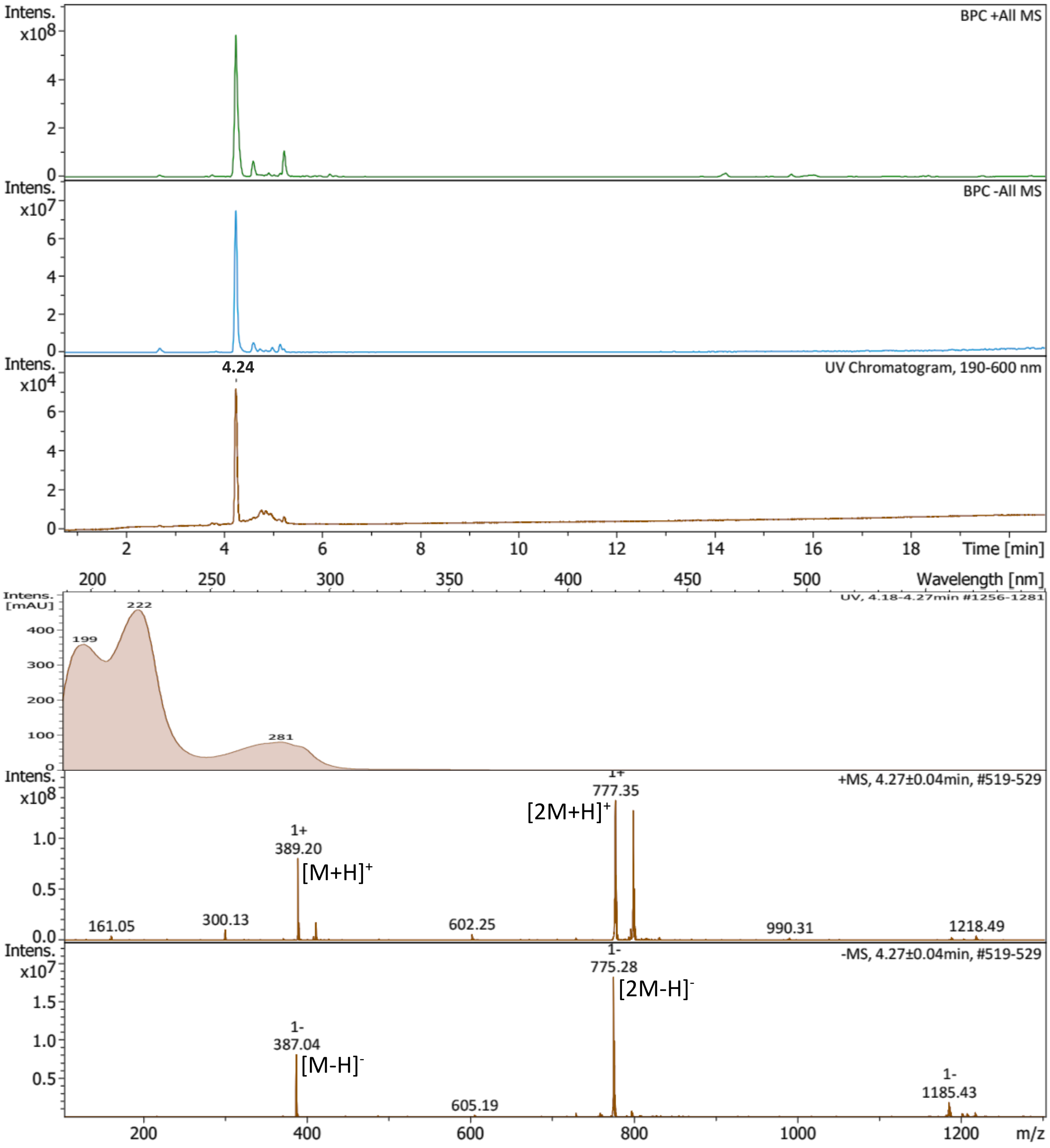

Bruker Compass DataAnalysis 6.1 printed: 13.11.2023 16:16:59 by: sel22 Page 1 of 1

Figure S41. LR-ESI-MS of **6**.

Display Report

Analysis Info

Analysis Name	S:\PEOPLE\cho23_Caren Holzenkamp\NMR\MS-Data\purified fractions\MeOH-F11-IIF7-397\MyNe_03_02_06_MeOH_F11_II_F7_P1-A-9_1_900.d	Acquisition Date	03.11.2023 02:15:59
Method	MWIS_BEH50mm_25min_ohnetims.m	Operator	Demo User
Sample Name	MyNe_03_02_06_MeOH_F11_II_F7	Instrument	timsTOF Pro 2
Comment			

Acquisition Parameter

Ion Polarity Positive

SPS Target Mass

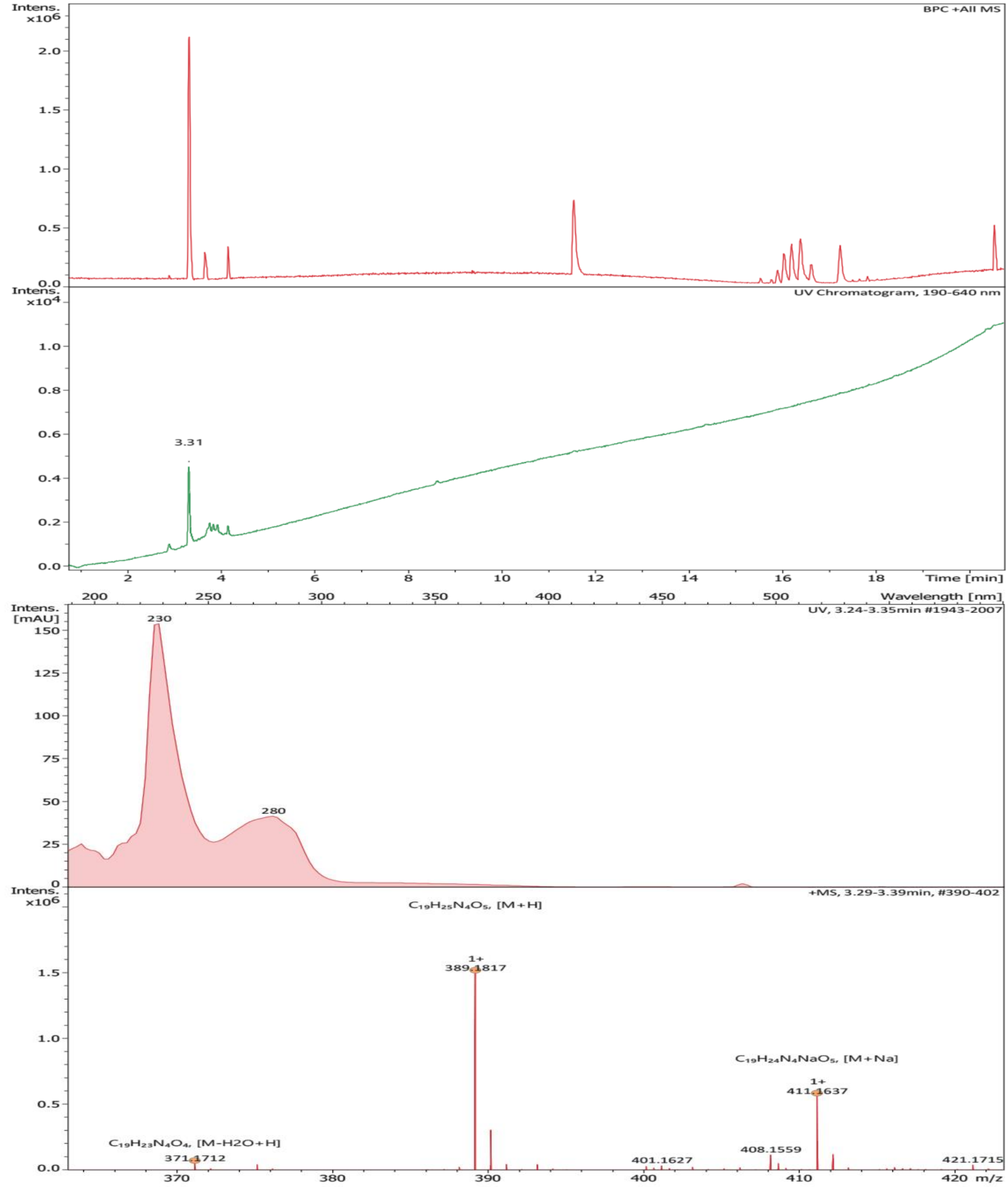

Bruker Compass DataAnalysis 6.1 printed: 13.11.2023 16:20:13 by: sel22 Page 1 of 1

Figure S42. HR-ESI-MS of **6**.

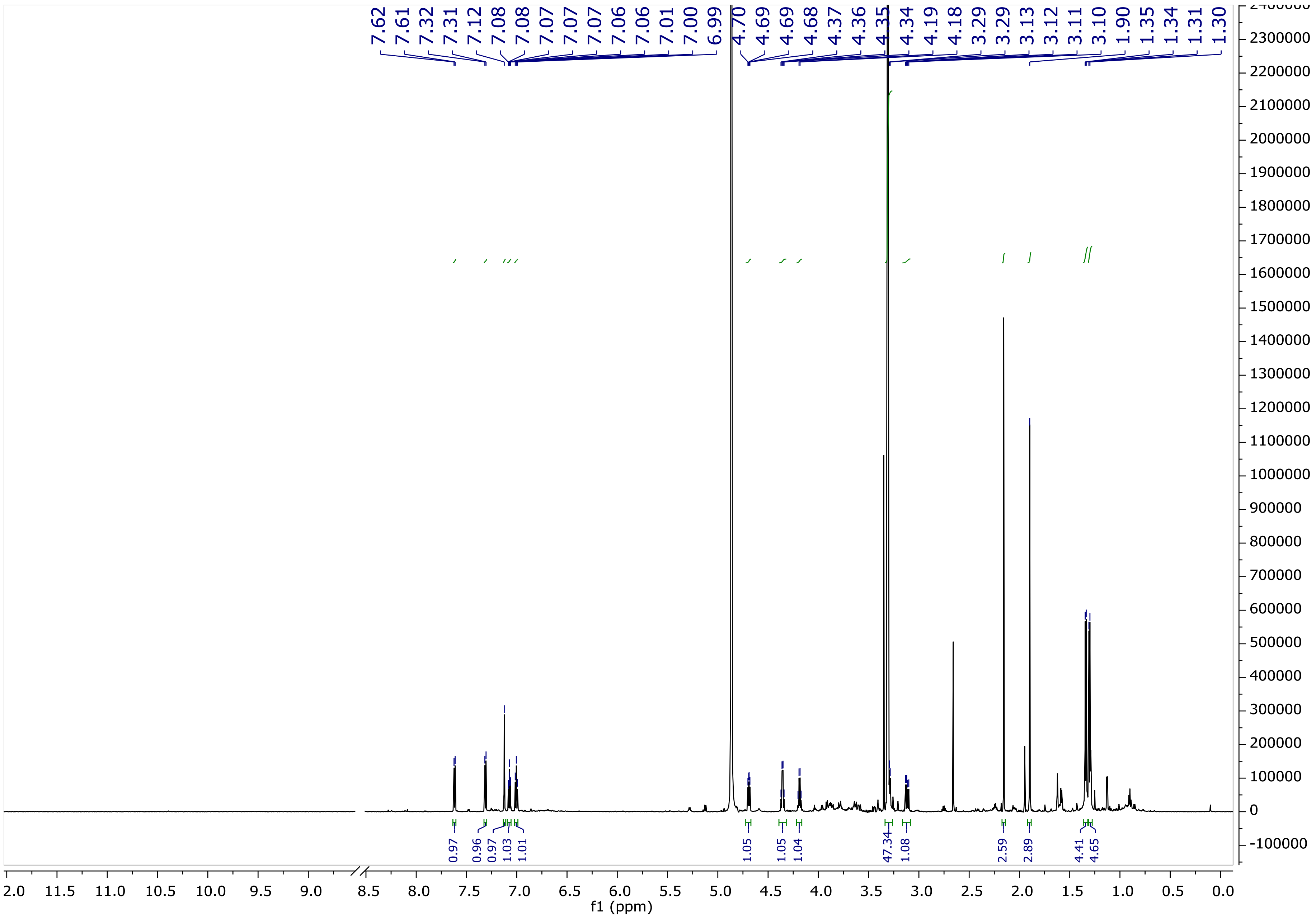

Figure S43. ^{1}H NMR spectrum of **6** in methanol-d_4 at 700 MHz.

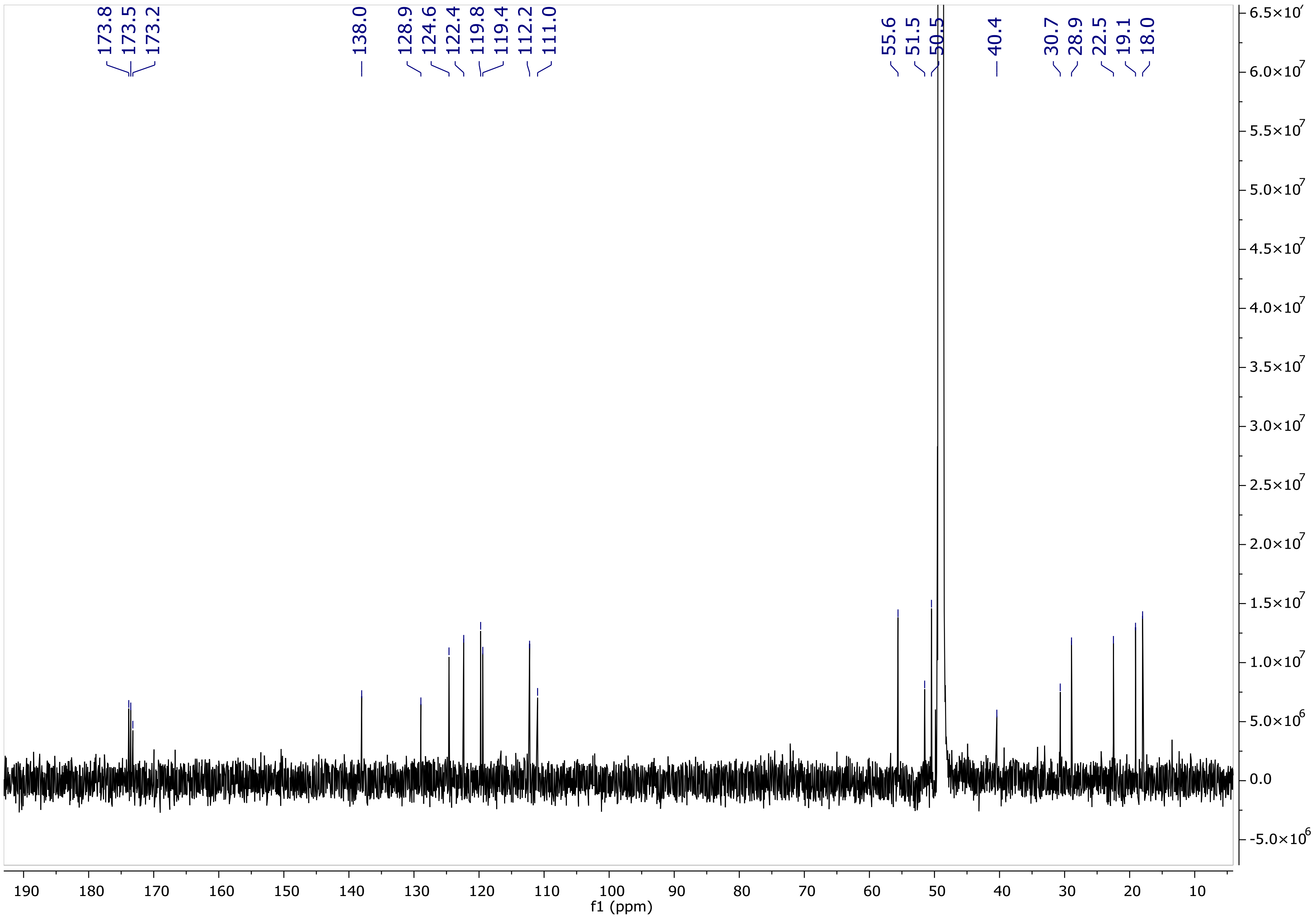

Figure S44. ^{13}C NMR spectrum of **6** in methanol-d_4 at 175 MHz.

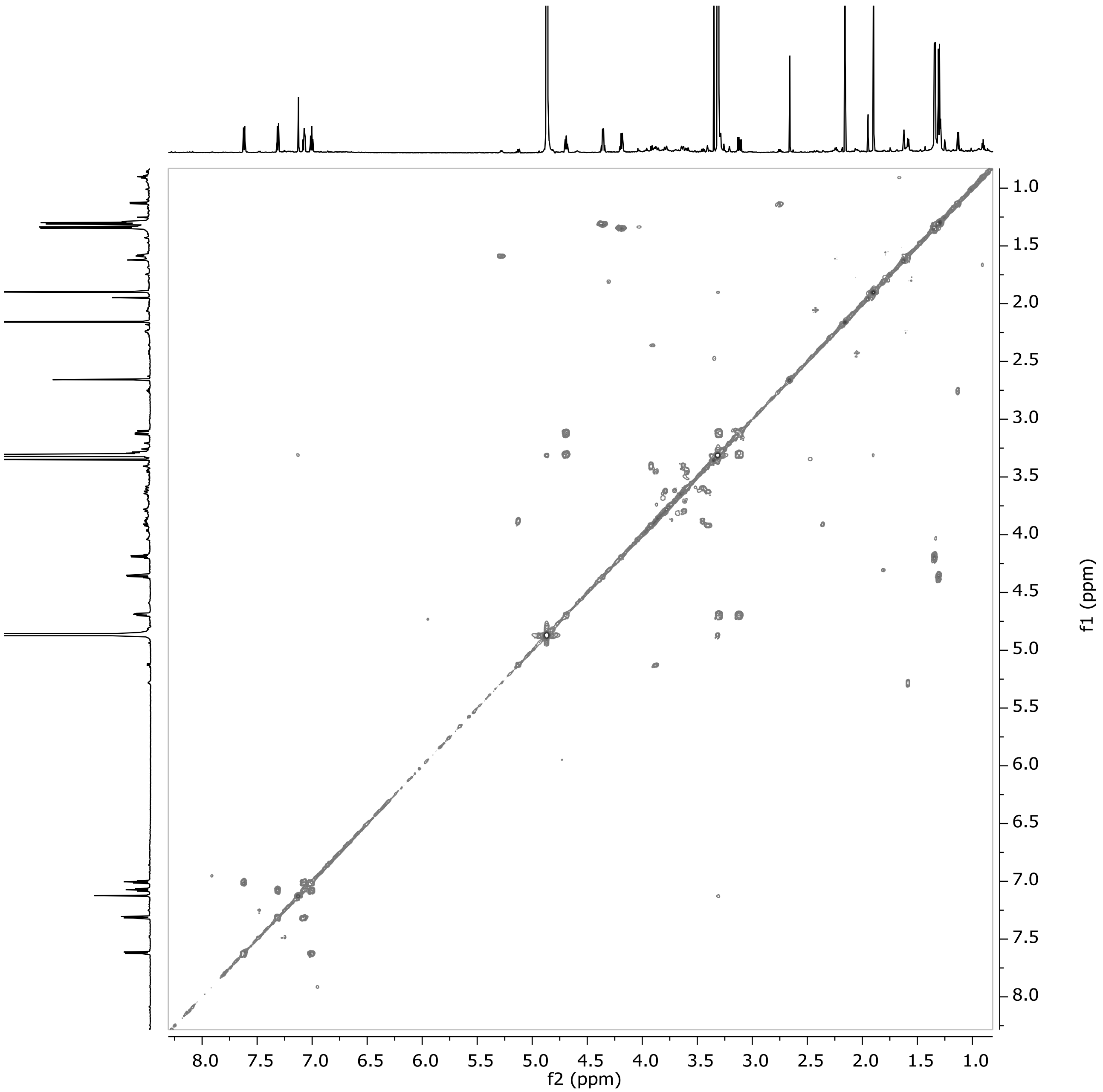

Figure S45. ^{1}H-^{1}H COSY spectrum of **6** in methanol-d_4 at 700 MHz.

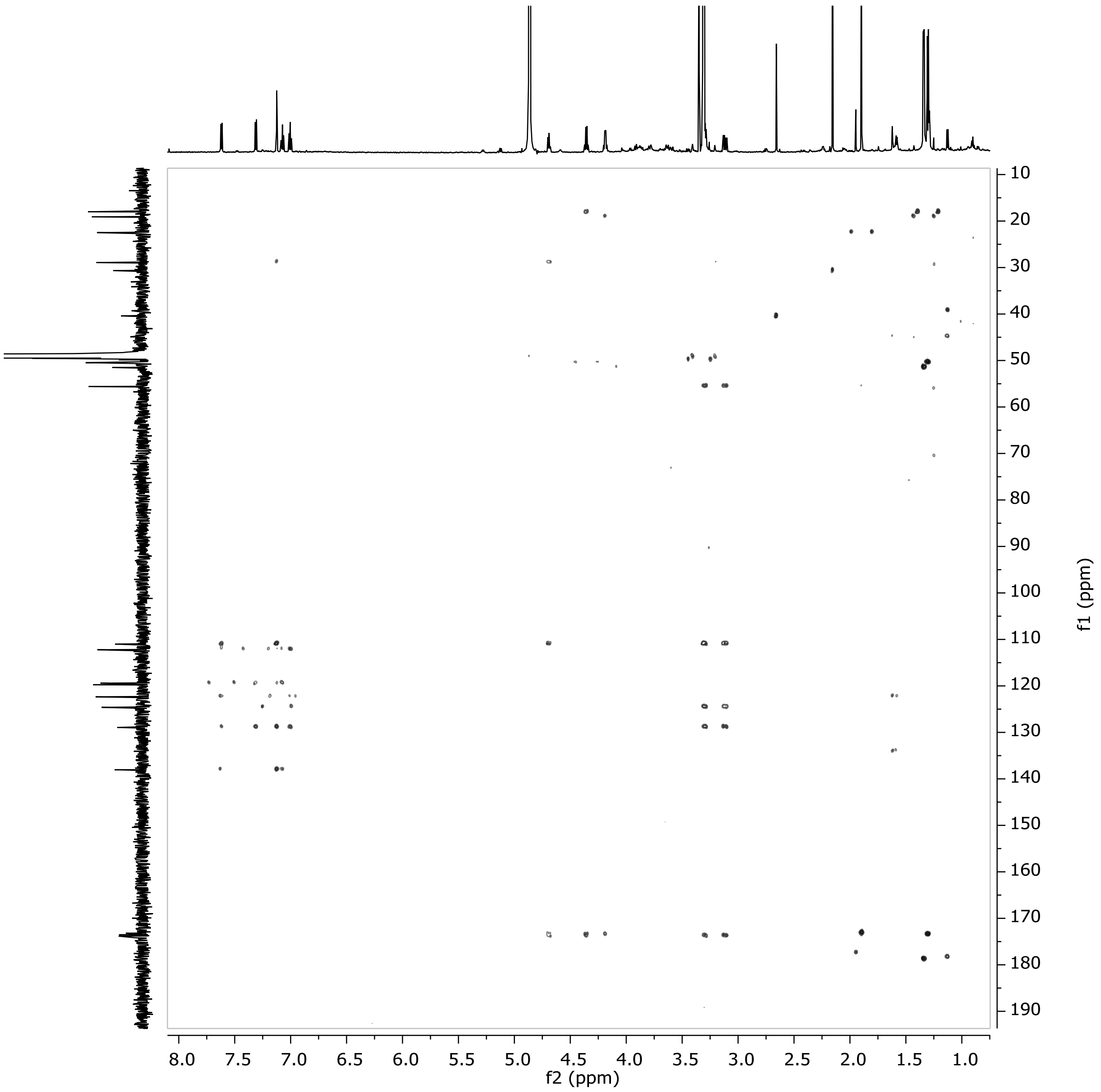

Figure S46. HMBC spectrum of **6** in methanol-d_4 at 700 MHz.

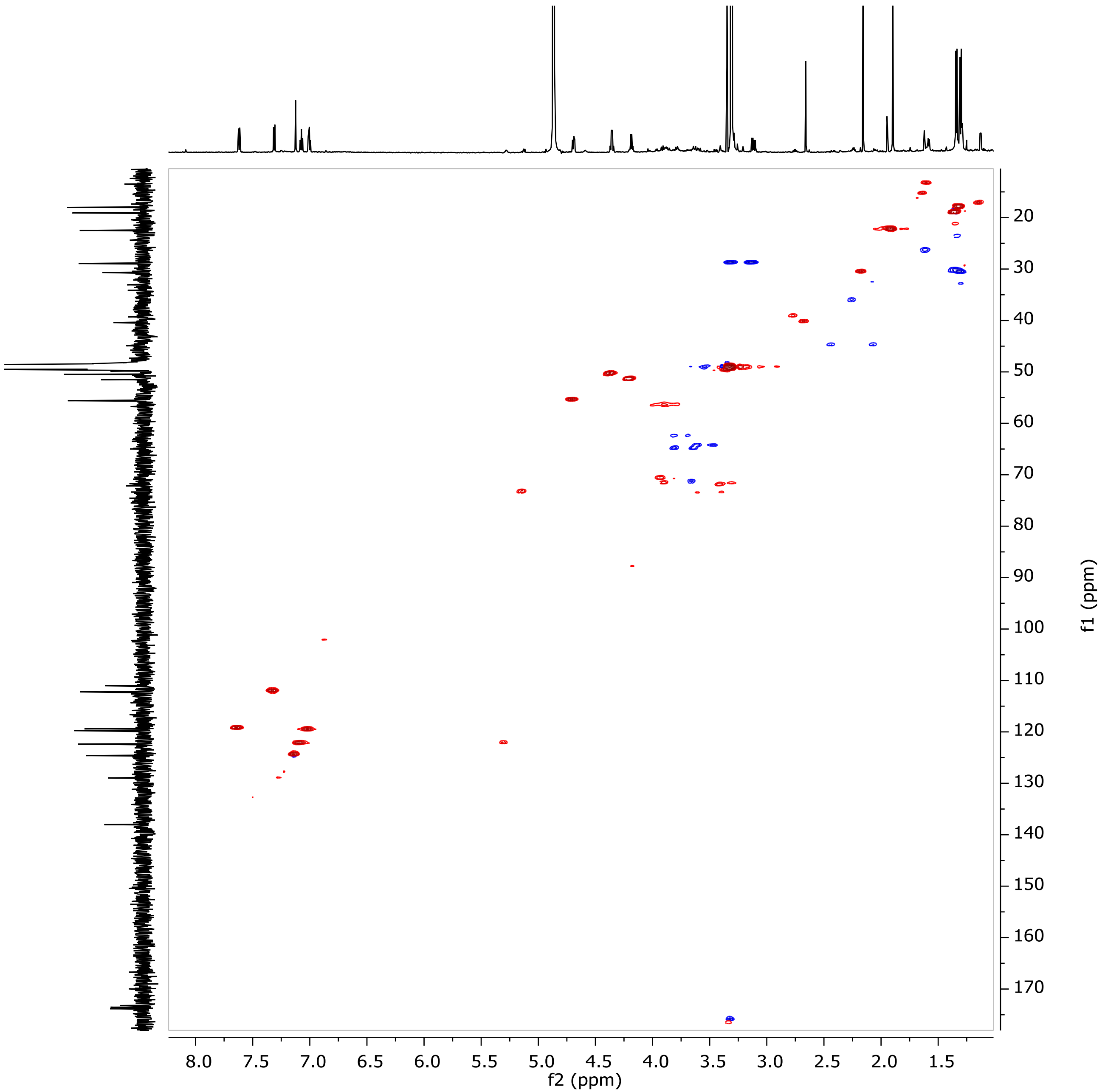

Figure S47. HSQC spectrum of **6** in methanol-d_4 at 700 MHz.

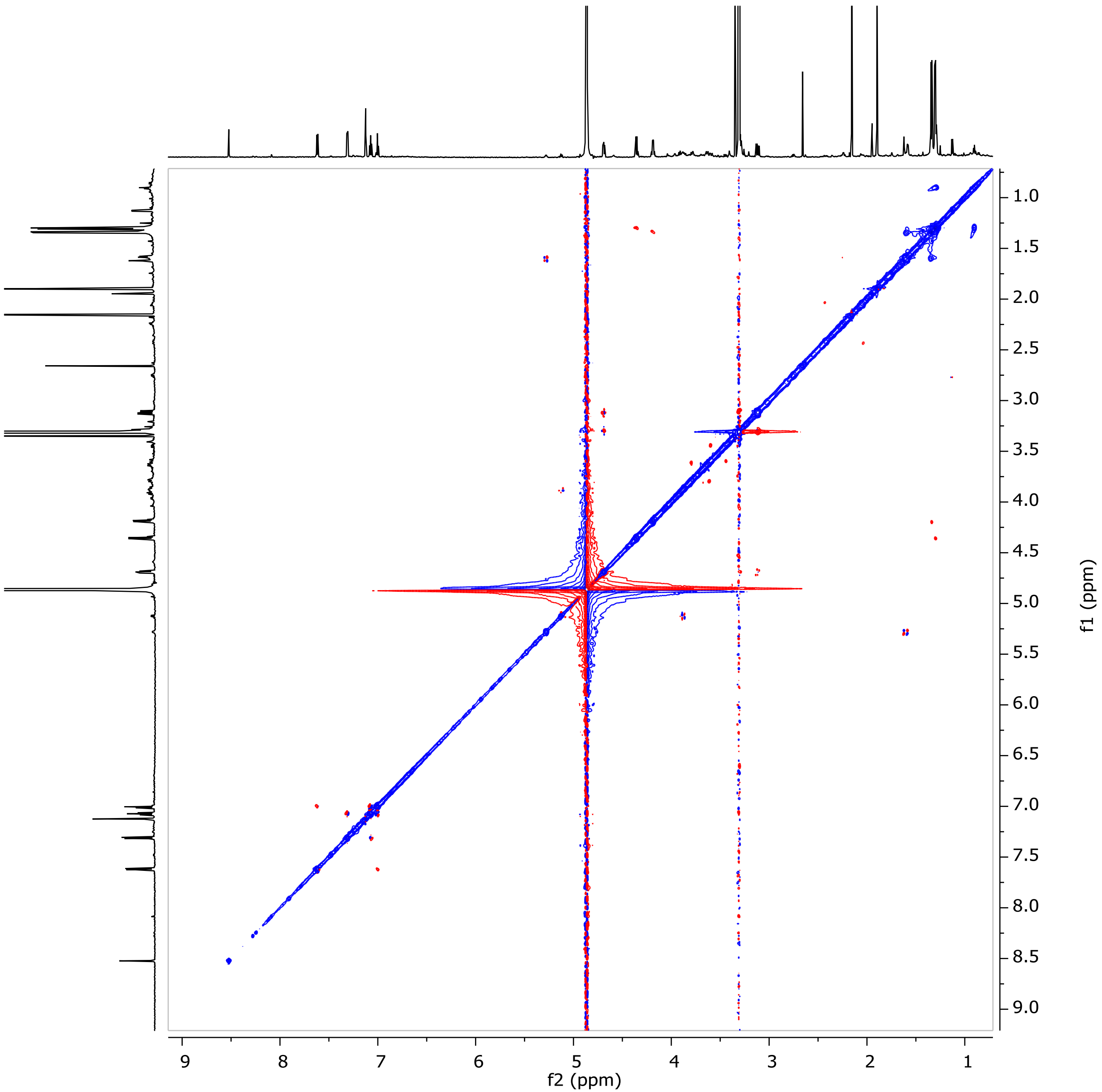

Figure S48. ROESY spectrum of **6** in methanol-d_4 at 700 MHz.

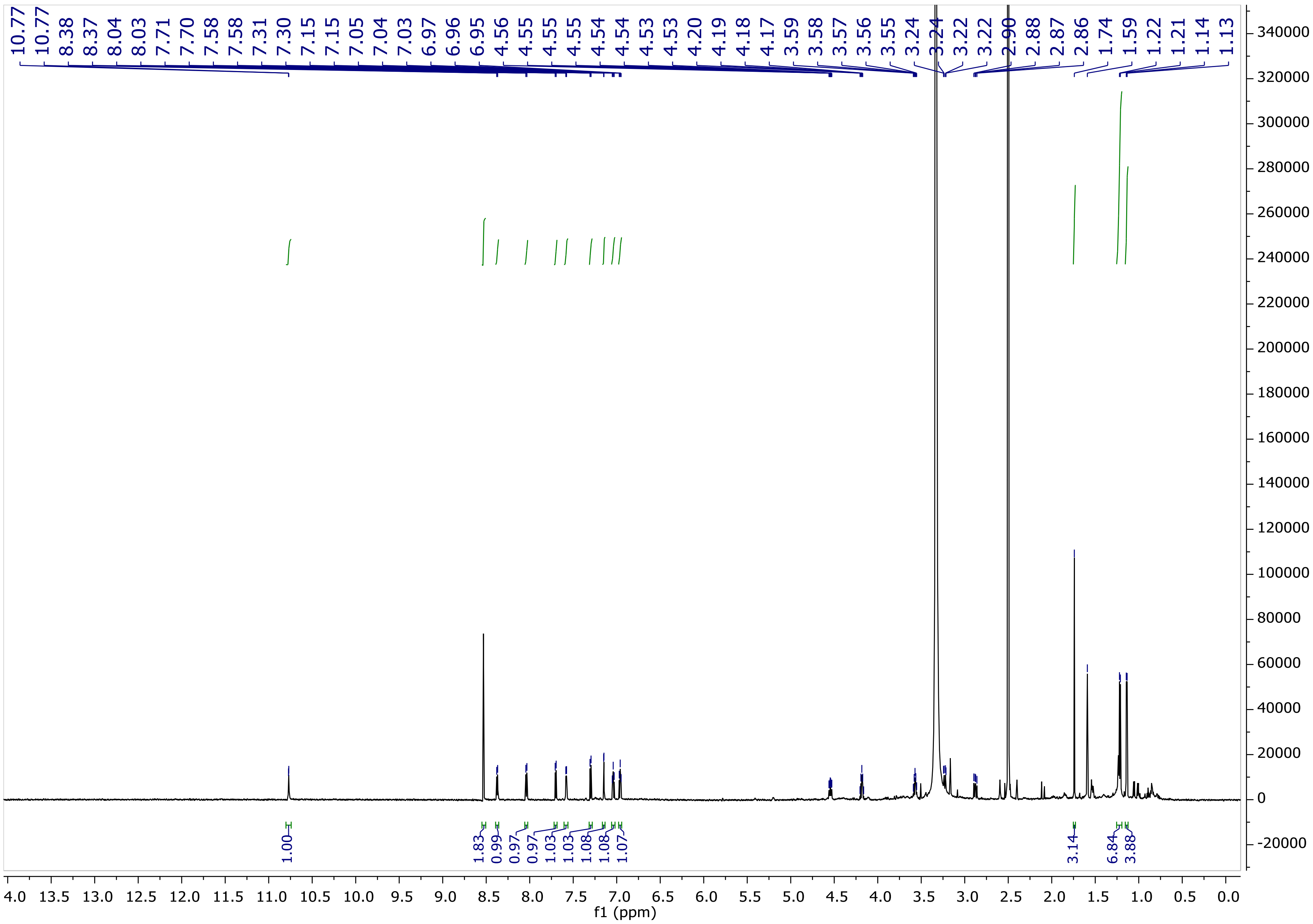

Figure S49. ^{1}H NMR spectrum of **6** in DMSO-d_6 at 700 MHz.

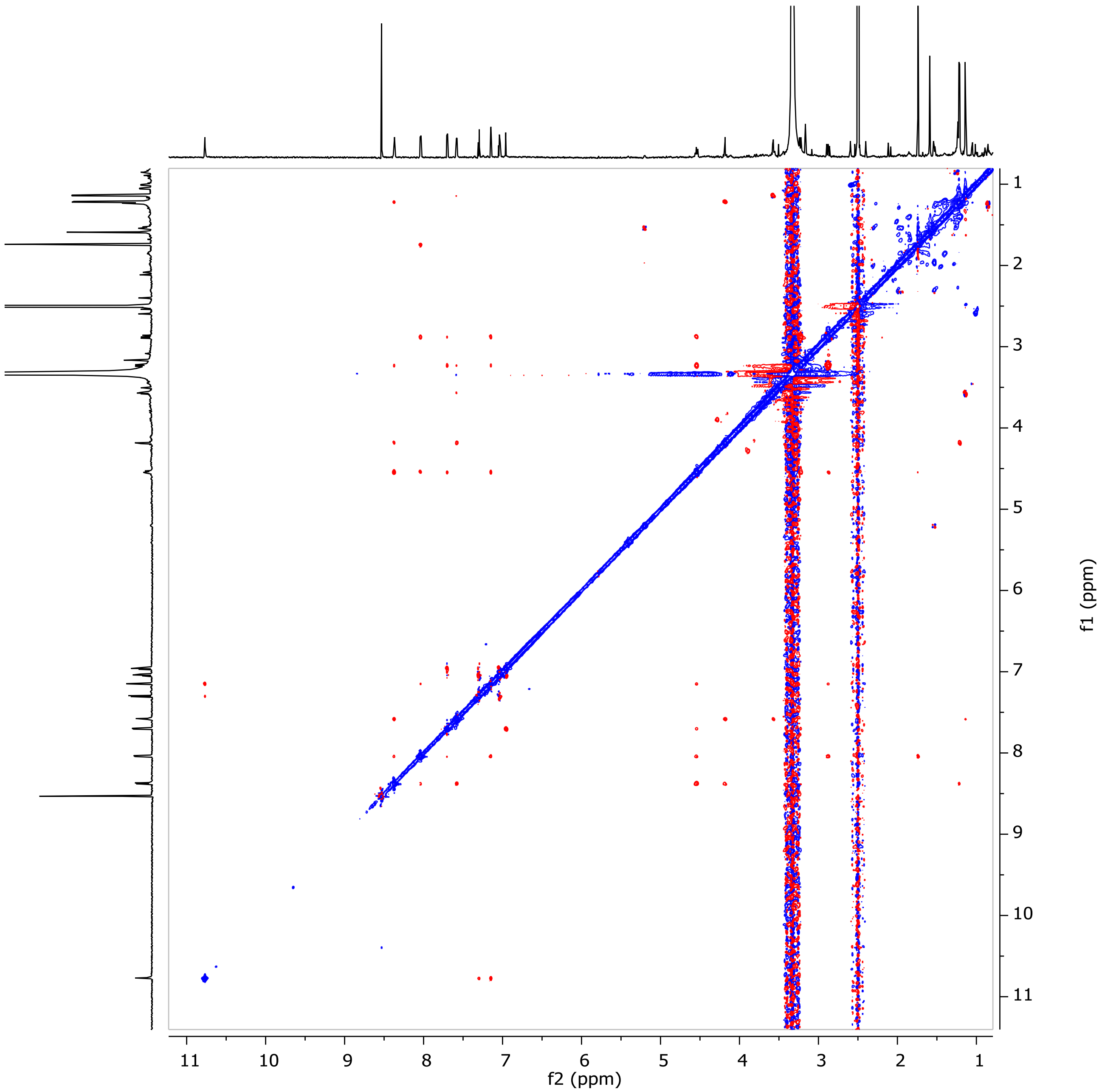

Figure S50. ROESY spectrum of **6** in DMSO-d_6 at 700 MHz.

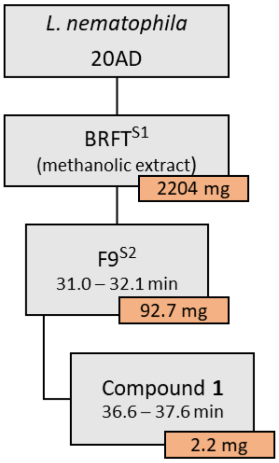

Figure S51. Overview of purification steps to isolate compound **1** from methanol extract of *Laburnicola nematophila* Strain 20AD cultivated on BRFT media.

Table S1. Pre-fractionation with NP-MPLC separation parameters for *Laburnicola nematophila* Strain K01 BRFT methanol extract.

**Parameter**	**Description**
Sample name	K01 BRFT MeOH
System	Grace Reveleris X2 flash system
Column	Büchi FlashPure ID Silica 24 g
Flow rate	32 mL min^{-1}
Sample Amount	2,204 mg
Runs	1
Solvents	A: dichloromethane (DCM) + 0.1 % FA;<br>B: 58% DCM, 40% acetone, 2% methanol + 0.1 % FA;<br>C: 35% DCM, 35% acetone, 30% methanol + 0.1 % FA

**Gradient**	
Accumulated time [min]	% B/C
0	0
5	0
25	100
30	100
30	C: 0
40	C: 100
50	C: 100

Table S2. Preparative RP-HPLC separation parameters used to isolate compound **1**.

**Parameter**	**Description**
Sample name	20AD BRFT MeOH extract-F9 (t_R = 31.0–32.1 min)
System	Gilson PLC 2250
Column	Luna C_{18} (2) column (250 × 21.2 mm, 5 µm)
Solvents	A: H_2O + 0.1 % FA; B: MeCN + 0.1 % FA
Flow rate	20 mL min^{-1}
Sample amount	92.7 mg
Runs	2
Isolated Compound	**1** (t_R = 36.6–37.6 min).

**Gradient**	
Accumulated time [min]	% B
0	25
5	25
55	75
60	100
70	100

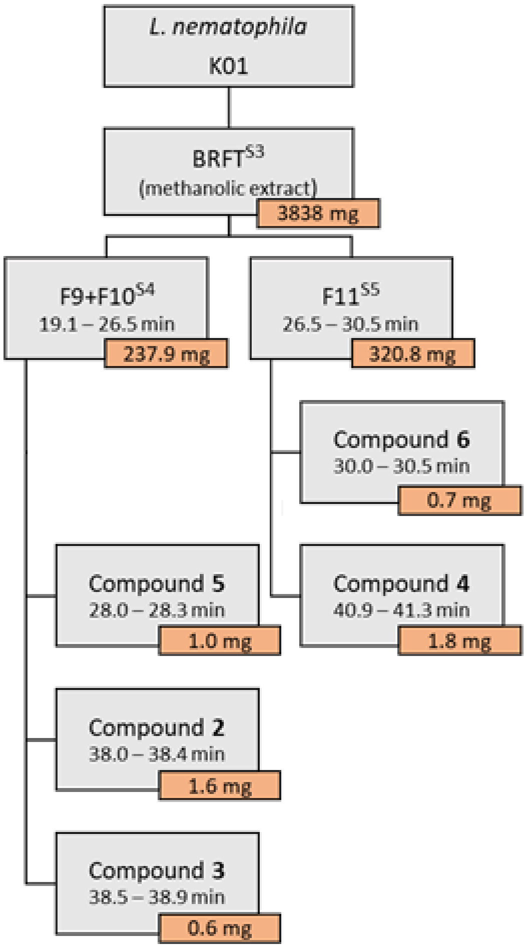

Figure S52. Overview of purification steps to isolate compounds (**2**–**6**) from methanol extract of *Laburnicola nematophila* Strain K01 cultivated on BRFT media.

Table S3. Pre-fractionation with NP-MPLC separation parameters for *Laburnicola nematophila* Strain K01 BRFT methanol extract.

**Parameter**	**Description**
Sample name	K01 BRFT MeOH
System	Grace Reveleris X2 flash system
Column	Büchi FlashPure ID Silica 40 g
Flow rate	40 mL min^{-1}
Sample Amount	3,838 mg
Runs	1
Solvents	A: dichloromethane (DCM) + 0.1 % FA;<br>B: 58% DCM, 40% acetone, 2% methanol + 0.1 % FA;<br>C: 35% DCM, 35% acetone, 30% methanol + 0.1 % FA

**Gradient**	
Accumulated time [min]	% B/C
0	0
5	0
25	100
30	100
30	C: 0
40	C: 100
50	C: 100

Table S4. Preparative RP-HPLC separation parameters used for F9+F10 to isolate compounds (**2**, **3** and **5**).

Parameter	Description	Gradient	
Sample name	K01 BRFT MeOH F9+F10 (t_R = 19.1–26.5 min)	Accumulated time [min]	% B
System	Büchi Pure C-850 FlashPrep	0	5
Column	Luna C_{18} (2) column (250 × 50 mm, 10 µm)	5	5
Flow rate	50 mL min^{-1}	65	50
Sample Amount	237.9 mg	70	50
Runs	2	80	55
Solvents	A: H_2O + 0.1 % FA; B: MeCN + 0.1 % FA		
Isolated Compound	**2** (t_R = 38.0–38.4 min), **3** (t_R = 38.5–38.9 min), **5** (t_R = 28.0–28.3 min).		

Table S5. Preparative RP-HPLC separation parameters used for F11 to isolate **4** and **6**.

Parameter	Description	Gradient	
Sample name	K01 BRFT MeOH F11 (t_R = 26.5–30.5 min)	Accumulated time [min]	% B
System	Büchi Pure C-850 FlashPrep	0	10
Column	Luna C_{18} (2) column (250 × 50 mm, 10 µm)	5	10
Flow rate	50 mL min^{-1}	55	60
Sample Amount	320.8 mg	60	100
Runs	2	70	100
Solvents	A: H_2O + 0.1 % FA; B: MeCN + 0.1 % FA		
Isolated Compound	**4** (t_R = 40.9–41.3 min), **6** (t_R = 30.0–30.5 min).		

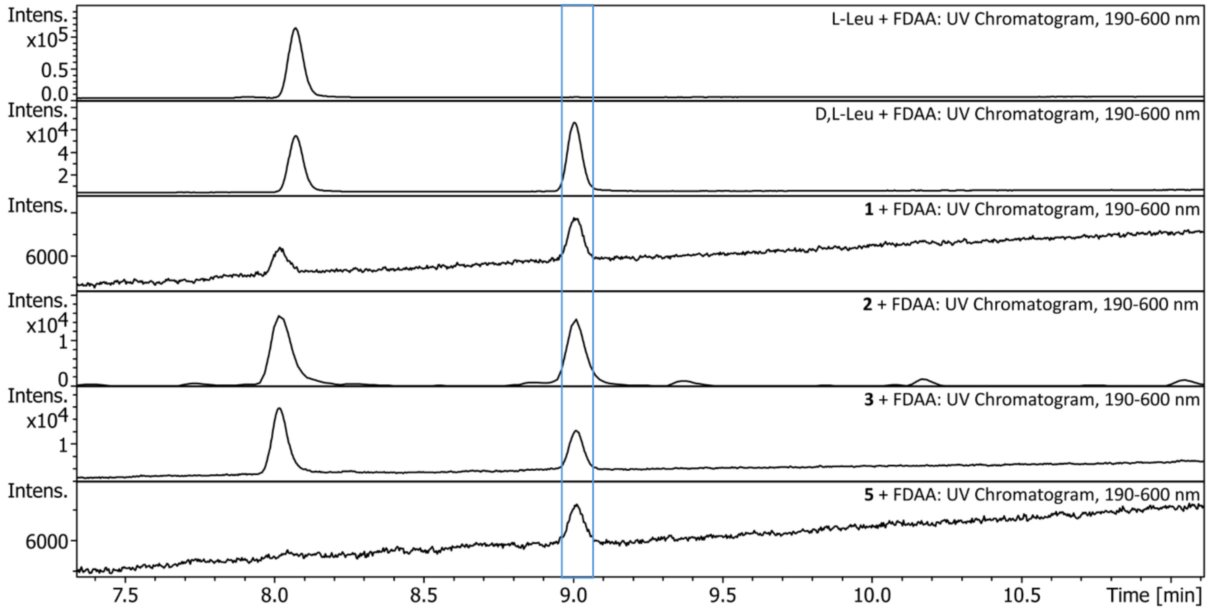

Figure S53. LC-ESI-MS spectra of L-leucine, DL-leucine, compounds (**1**, **2**, **3** and **5**) + FDAA. Top-bottom: L-leucine + FDAA, DL-leucine + FDAA, (**1**, **2**, **3** and **5**) + FDAA. UV-chromatogram at 190–600 nm is shown. Bars are indicating identical MS-Peaks (blue D-leucine).

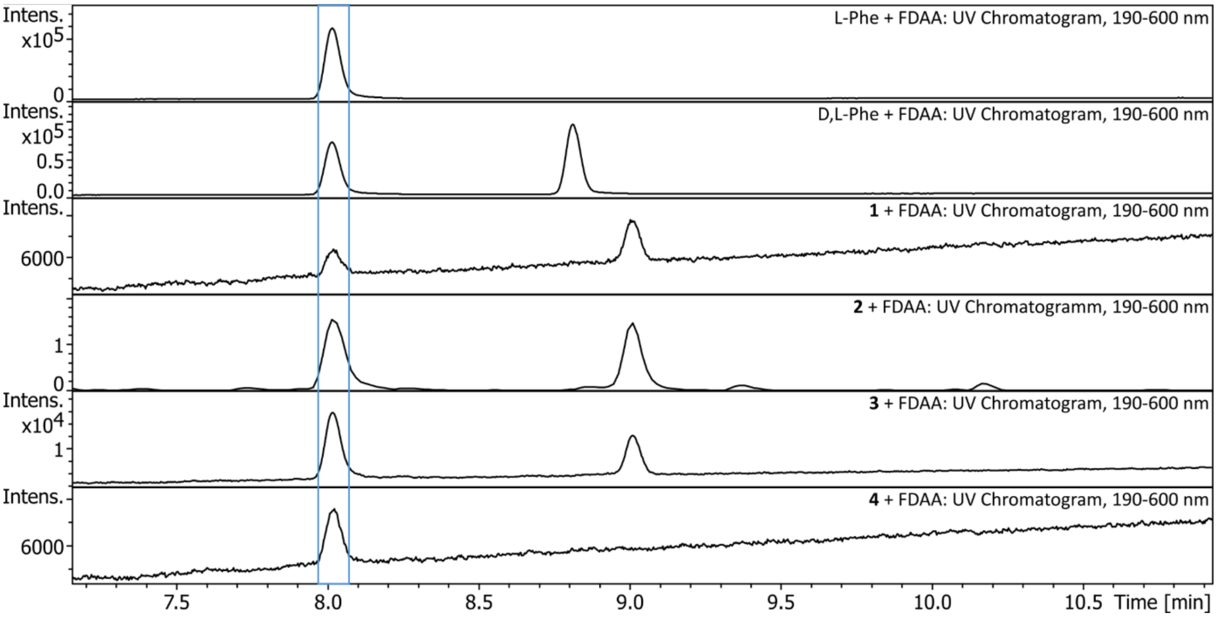

Figure S54. LC-ESI-MS spectra of DL-phenylalanine, L-phenylalanine, compounds (**1**, **2**, **3** and **4**) + FDAA. Top-bottom: DL-phenylalanine + FDAA, L-phenylalanine + FDAA, (**1**, **2**, **3** and **4**) + FDAA. UV-chromatogram at 190–600 nm is shown. Bars are indicating identical MS-Peaks (blue L-phenylalanine).

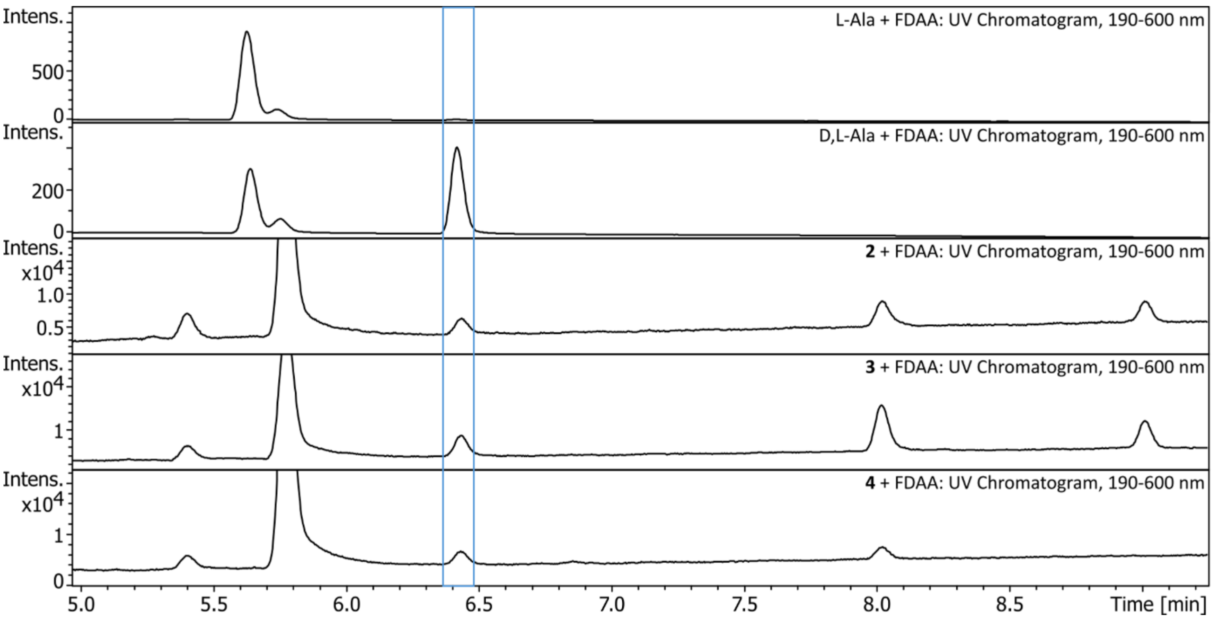

Figure S55. LC-ESI-MS spectra of L-alanine, DL-alanine, compounds (**2**, **3** and **4**) + FDAA. Top-bottom: L-alanine + FDAA, DL-alanine + FDAA, **2**-**4** + FDAA. UV-chromatogram at 190–600 nm is shown. Bars are indicating identical MS-Peaks (blue D-alanine).

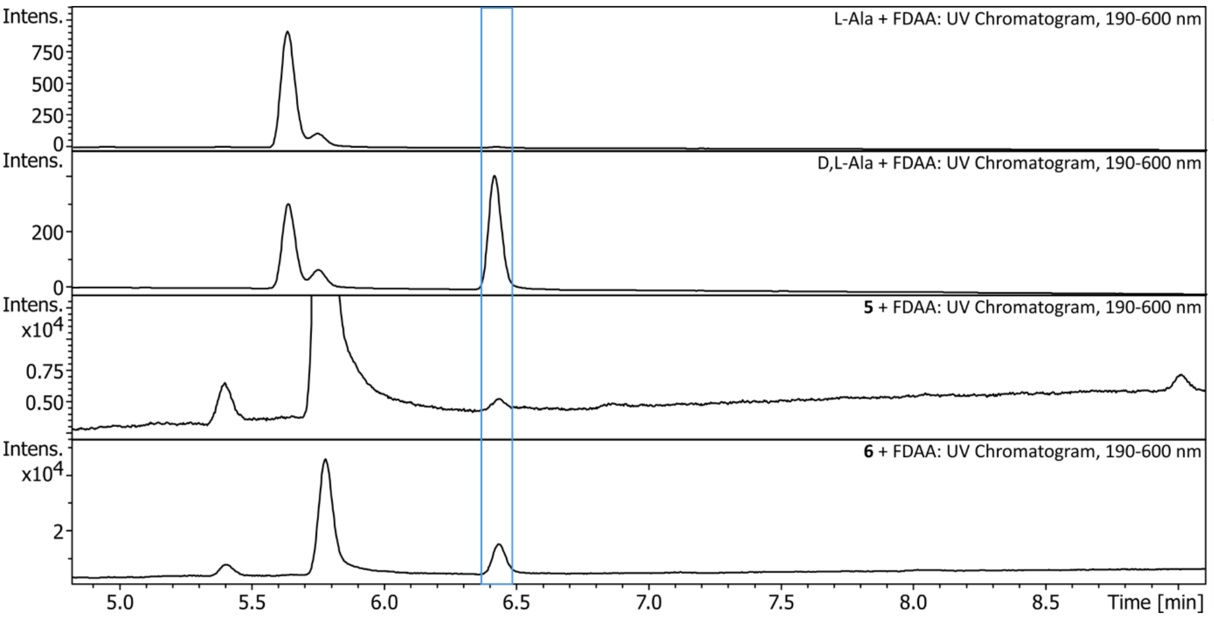

Figure S56. LC-ESI-MS spectra of L-alanine, DL-alanine, compounds (**5** and **6**) + FDAA. Top-bottom: L-alanine + FDAA, DL-alanine + FDAA, **5**/**6** + FDAA. UV-chromatogram at 190–600 nm is shown. Bars are indicating identical MS-Peaks (blue D-alanine).

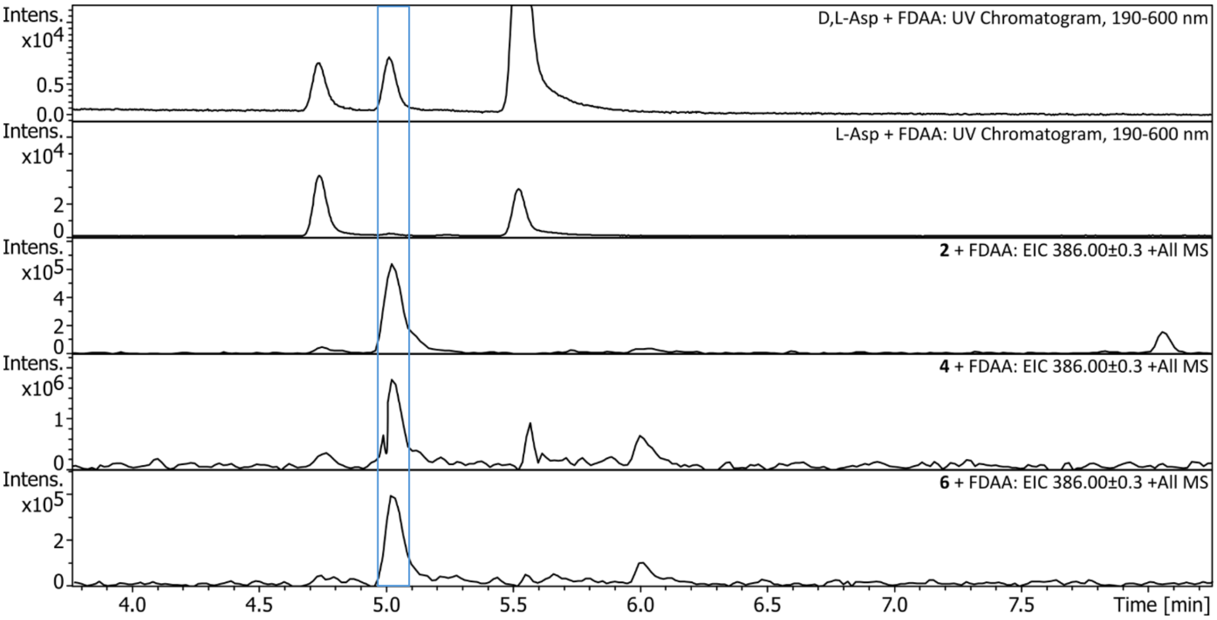

Figure S57. LC-ESI-MS spectra of DL-aspartic acid, L-aspartic acid, compounds (**2**, **4** and **6**) + FDAA. Top-bottom: DL-aspartic acid + FDAA, L-aspartic acid + FDAA, (**2**, **4** and **6**) + FDAA. UV-chromatogram at 190–600 nm and is shown. Bars are indicating identical MS-Peaks (blue L-phenylalanine).

Table S6. Cytotoxicity (IC_{50}) of **1**–**6**.

Test Cell Line	IC_{50} (µM)						Positive Control
	**1**	**2**	**3**	**4**	**5**	**6**	Epothilone B (nM)
Mouse fibroblast (L929)	-	-	n.d.	-	-	n.d.	0.65
Human endocervival adenocarcinoma (KB3.1)	-	-	n.d.	-	-	n.d.	0.17

-: No effect on cell lines. n.d.: Not determined.

Table S7. Antimicrobial activity (MIC) of **1**–**6**.

Test Microorganism	MIC (µg/mL)						Positive Control (µg/mL)
	**1**	**2**	**3**	**4**	**5**	**6**	
Staphylococcus aureus	-	-	n.d.	-	-	n.d.	0.21[G]
Escherichia coli	-	-	n.d.	-	-	n.d.	0.42[G]
Bacillus subtilis	-	-	n.d.	-	-	n.d.	16.6[O]
Pseudomonas aeruginosa	-	-	n.d.	-	-	n.d.	0.21[G]
Pichia anomala	-	-	n.d.	-	-	n.d.	16.6[N]
Candida albicans	-	-	n.d.	-	-	n.d.	8.3[N]
Acinetobacter baumanii	-	-	n.d.	-	-	n.d.	0.52[C]
Chromobacterium violaceum	-	-	n.d.	-	-	n.d.	1.70[G]
Schizosaccharomyces pombe	-	-	n.d.	-	-	n.d.	8.30[N]
Mucor hiemails	-	-	n.d.	-	-	n.d.	8.30[N]
Rhodotorula glutinis	-	-	n.d.	-	-	n.d.	4.20[N]
Mycobacterium smegmatis	-	-	n.d.	-	-	n.d.	1.70[K]

-: No inhibition up to 67 µg/mL. n.d.: Not determined.

G: Gentamycin; O: Oxytetracycline; N: Nystatin; C: Ciprofloxacin; K: Kanamycin.

Table S8. Nematicidal activity of **1**, **2**, **4** and **5**.

Test Organism	Strain number	Conc. (µg mL^{-1})	Corrected Mortality (%)				Positive Control
			**1**	**2**	**4**	**5**	Ivermectin
Caenorhabtidis elegans	N2	100	< 0	< 0	< 0	< 0	n.d.
		50	< 0	< 0	0.1 ± 2.2	0.5 ± 1.1	
		10	< 0	< 0	0.7 ± 2.0	< 0	
		1	n.d.	n.d.	n.d.	n.d.	96.2 ± 2.7

n.d.: not determined. < 0: less active then the corresponding negative control.
